# Calcitriol Suppresses Platelet Activation and Thrombosis, Mitigating Cardiovascular Risks in Metabolic Dysfunction–Associated Steatotic Liver Disease

**DOI:** 10.1016/j.jacbts.2026.101632

**Published:** 2026-07-10

**Authors:** Peng Zhang, Huajie Xu, Shujing Wu, Wenxuan Zhou, Luning Zhou, Liping Han, Wei Zhang, Xiaoyu Lian, Xin Zhao, Haoxuan Zhong, Bing Fan, Hongyi Wu, Zhiyong Qi

**Affiliations:** a Department of Cardiology, Zhongshan Hospital, Fudan University, Shanghai, China; b Institutes of Biomedical Sciences, Fudan University, Shanghai, China; c National Clinical Research Center for Interventional Medicine, Zhongshan Hospital, Fudan University, Shanghai, China; d State Key Laboratory of Cardiovascular Diseases, Zhongshan Hospital, Fudan University, Shanghai, China; e NHC Key Laboratory of Ischemic Heart Diseases, Zhongshan Hospital, Fudan University, Shanghai, China; f Key Laboratory of Viral Heart Diseases, Chinese Academy of Medical Sciences, Zhongshan Hospital, Fudan University, Shanghai, China; g Department of Infectious Disease, Zhongshan Hospital, Fudan University, Shanghai, China; h Department of Cardiology, Zhongshan Hospital (Xiamen), Fudan University, Xiamen, Fujian, China; i Department of Biochemistry and Molecular Biology, NHC Key Laboratory of Glycoconjugates Research, School of Basic Medical Sciences, Fudan University, Shanghai, China

**Keywords:** calcitriol, metabolic dysfunction–associated steatotic liver disease, platelet, thrombosis

## Abstract

Metabolic dysfunction–associated steatotic liver disease is associated with vitamin D deficiency, and platelets exhibit hyperactivity. Zhang et al provide novel insights into the mechanisms underlying platelet hyperreactivity and thrombotic risk in patients with metabolic dysfunction–associated steatotic liver disease, demonstrating that calcitriol activates the vitamin D receptor in megakaryocytes and platelets, leading to the downregulation of P2Y_12_ expression and the modulation of the cyclic adenosine monophosphate/protein kinase A and mitogen-activated protein kinase signaling pathways, thereby attenuating platelet activation.

## Highlights

• Calcitriol exerts a negative regulatory effect on platelet activity by downregulating P2Y_12_ expression and modulating the cAMP/PKA and MAPK signaling pathways.
• Calcitriol mitigates metabolic dysfunction–associated steatotic liver disease–associated platelet hyperreactivity, thereby inhibiting in vivo thrombus formation and protecting vital organs from severe infarction.
• Calcitriol replenishment shows potential to mitigate ischemic risks in MAFLD patients.

## Visual Abstract

**Figure:**
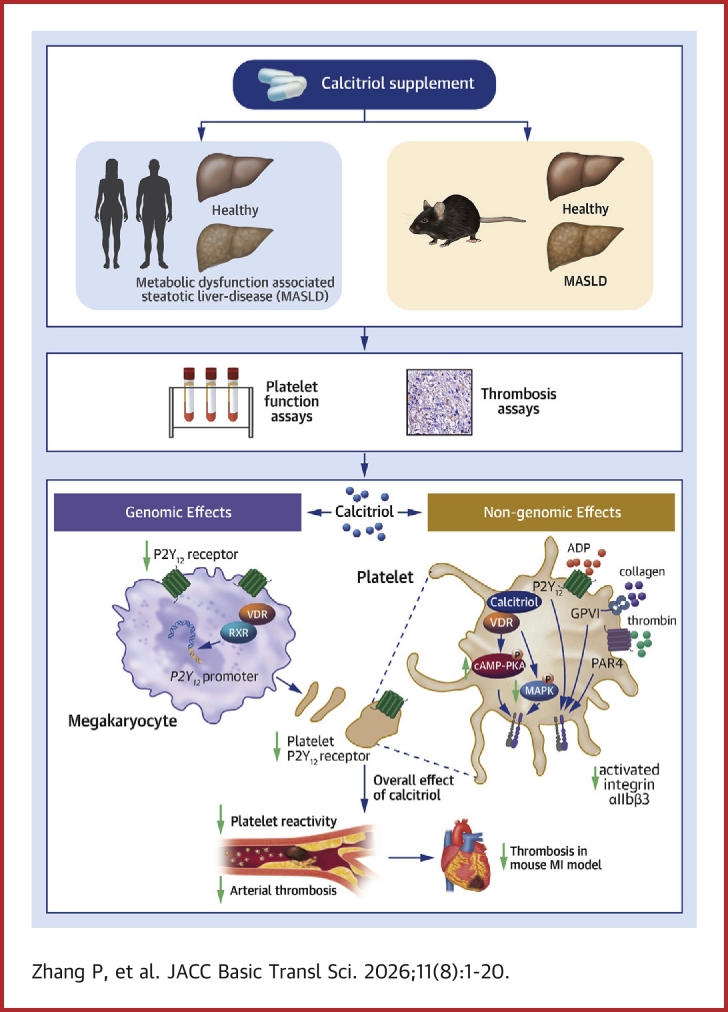

Vitamin D, a fat-soluble secosteroid hormone, primarily circulates in the bloodstream as 25-hydroxyvitamin D [25(OH)D] and exerts its biological functions in its active form, 1,25-dihydroxyvitamin D_3_ [1,25(OH)_2_D_3_], also known as calcitriol.^1^ Although traditionally recognized for its role in bone and mineral metabolism, its potential impact on the cardiovascular system has been a subject of extensive investigation.^2^ Although recent large-scale randomized controlled trials have reported neutral results regarding the benefits of vitamin D supplementation for cardiovascular prevention,^3^, ^4^, ^5^ extensive observational data continue to link low plasma 25(OH)D levels with elevated risks of ischemic heart disease,^6^ myocardial infarction (MI),^7^ ischemic stroke (IS),^8^^,^^9^ and peripheral artery disease.^10^ These conditions share a common pathophysiological mechanism: thrombosis formation in the arterial circulation, a process involving platelet activation.^11^

In recent years, the relationship between plasma vitamin D levels and platelet reactivity has garnered increasing attention. Studies have reported a negative correlation between plasma 25(OH)D concentration and mean platelet volume, a hallmark of platelet hyperreaction,^12^ as well as a reversed correlation between plasma vitamin D and platelet fibrinogen binding in individuals with circulating vitamin D levels below 50 nmol/L.^13^ However, the direct effects of vitamin D on platelet activation and the associated mechanisms remain incompletely characterized.

Metabolic dysfunction–associated steatotic liver disease (MASLD) is a prevalent global health concern and a significant risk factor for cardiovascular disease (CVD).^14^, ^15^, ^16^, ^17^, ^18^ In fact, CVD is the primary cause of mortality among patients with MASLD, outpacing liver-related complications.^14^^,^^18^^,^^19^ Extensive research demonstrates a robust association between MASLD and vitamin D deficiency,^20^, ^21^, ^22^ and platelets in patients with MASLD exhibit hyperactivity.^23^^,^^24^ However, the specific interplay between diminished plasma vitamin D levels and heightened platelet activity, and its potential contribution to thrombogenesis, requires further investigation.

In this study, we found that calcitriol exhibited a direct suppressive effect on platelet activation and in vivo thrombus formation. Mechanistic studies elucidated that calcitriol, on binding to the vitamin D receptor (VDR), downregulates P2Y_12_ receptor expression in megakaryocytes and concurrently modulates platelet signaling by activating the cyclic adenosine monophosphate (cAMP)/protein kinase A (PKA) pathway and inhibiting the mitogen-activated protein kinase (MAPK) pathway. Moreover, a negative correlation between plasma calcitriol concentration and platelet aggregation was observed in patients with MASLD. Consequently, our study indicates a potential role for calcitriol interventions in mitigating thrombotic risk in this population.

## Methods

Detailed materials and methods are provided in the Supplemental Methods.

### Human studies

All human studies and experimental procedures were conducted in compliance with the Declaration of Helsinki and approved by the institutional review board of Fudan University Zhongshan Hospital. Informed consent was obtained from all participants before enrollment.

To investigate the potential association between plasma calcitriol levels and platelet reactivity in healthy participants, we enrolled 143 healthy participants at Fudan University Zhongshan Hospital. We excluded participants with CVD, MASLD, renal dysfunction, diabetes mellitus (DM), hypertension, hyperlipidemia, malignancy, pregnancy, infection, hepatic surgery, antiplatelet medication use, or psychiatric disorders. After applying these exclusion criteria, 25 participants were excluded, leaving 118 participants for analysis.

Our initial intervention trial evaluated the impact of calcitriol on platelet activation in 10 healthy volunteers aged 18 years or older. Participants were recruited from Fudan University and excluded if they had recent antiplatelet drug use, hypercalcemia, hyperparathyroidism, osteomalacia, kidney stones, sarcoidosis, DM, significant chronic illnesses, or were pregnant. All participants were in good general health with no self-reported CVD.

To examine the link between plasma calcitriol levels and platelet reactivity in MASLD, we recruited 276 consecutive patients with MASLD from Fudan University Zhongshan Hospital. All participants were first-time visitors without prior MASLD-related or antiplatelet treatments. We excluded individuals with CVD, renal impairment, DM, cancer, pregnancy, infections, hepatic surgery, or psychiatric conditions. After applying these criteria, 187 patients were ineligible, leaving 89 patients with MASLD without antiplatelet drug use before blood sample collection.

We also conducted an intervention trial evaluated the impact of calcitriol on platelet activation in 10 patients with MASLD. Participants were recruited from Fudan University Zhongshan Hospital and excluded if they had CVD, hypercalcemia, hyperparathyroidism, osteomalacia, sarcoidosis, kidney stones, renal dysfunction, malignancy, pregnancy, active infection, a history of hepatic surgery, or psychiatric disorders. All participants were first-visit patients with MASLD and had no prior history of MASLD-related or antiplatelet treatment.

### Animal studies

All animal procedures followed the National Institutes of Health Guidelines for Laboratory Animal Care (NIH Publication No. 85-23, revised 1996) and were approved by the Zhongshan Hospital, Fudan University Animal Care and Use Committee. Wild-type (WT) C57BL/6J and VDR-deficient (*Vdr*^*−/−*^) mice were obtained from Shanghai JieSiJie Laboratory Animals and SaiYe Laboratory, respectively. Mice had unrestricted access to water and standard chow and were housed in specific pathogen-free facilities with a 12-hour light-dark cycle. Pentobarbital sodium was administered intraperitoneally for both euthanasia (200 mg/kg) and anesthesia (50 mg/kg). Euthanasia was performed in a CO_2_ chamber followed by cervical dislocation. Death was confirmed by the absence of heartbeats and reflexes.

### Statistical analysis

Categorical variables are presented using counts and percentages and continuous variables as the mean ± SD or median (25th and 75th percentiles [(Q1, Q3]) based on data distribution. Normality was assessed using the Shapiro-Wilk test. For normally distributed data with a single variable, differences between 2 groups were compared using an unpaired Student's *t*-test. For non-normally distributed data, the Mann-Whitney *U* test was used. Comparisons among more than 2 groups with 1 variable were performed using 1-way analysis of variance with Dunnett's (comparing the mean of each column with the mean of a control column) or Sidak's (comparing the means of preselected pairs of columns) multiple comparison tests for independent data. For normally distributed data with 2 variables, differences were evaluated using 2-way analysis of variance with Tukey's multiple comparisons test. For non-normally distributed data, the Kruskal-Wallis test was used. The paired-samples *t*-test was used to compare change from baseline for continuous variables. The correlation between the platelet aggregation ratio and plasma calcitriol levels was investigated using the nonparametric Spearman's rank correlation test (*r*). For all statistical analyses, *P* < 0.05 was considered statistically significant. Statistical analyses were conducted using GraphPad Prism (version 8.0; GraphPad Inc).

## Results

### Ingestion of calcitriol inhibits the activity of human platelets

**Figure 1:**
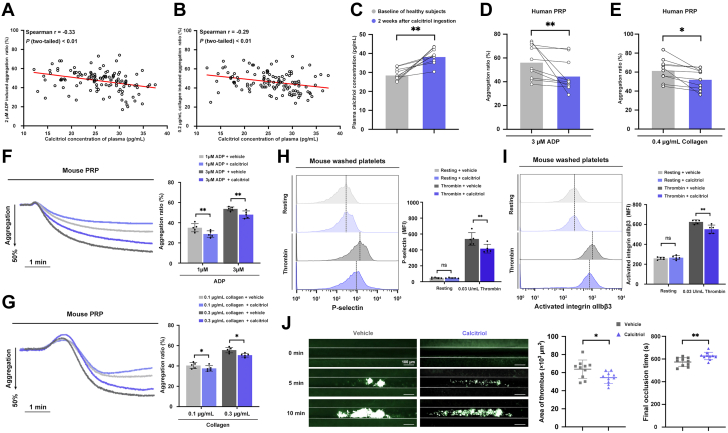
Ingestion of Calcitriol Attenuates Platelet Activation and In Vivo Thrombosis (A and B) Platelet aggregation in healthy participants (n = 118), induced by ADP (A) or collagen (B) and measured by aggregometry, exhibited a negative correlation with plasma calcitriol levels (Spearman *r* = −0.33, *P* < 0.01 for ADP; Spearman *r* = −0.29, *P* < 0.01 for collagen). Each data point represents a single individual. (C) Plasma calcitriol levels in healthy volunteers before and after 2 weeks of calcitriol supplementation (n = 10, 4 males). (D and E) Platelet aggregation in PRP from healthy volunteers before and after calcitriol ingestion in response to 3 μM ADP (D) or 0.4 μg/mL collagen (E) (n = 10, 4 males). Each data point represents the average value calculated from 5 separate measurements of platelet aggregation rate. (F and G) Platelet aggregation induced by 2 concentrations of ADP (F) or collagen (G) in PRP from calcitriol-treated and control WT mice (n = 5). (H) Evaluation of P-selectin surface expression on resuspended mouse platelets isolated from calcitriol-treated and control WT mice (n = 5), following stimulation with 0.03 U/mL thrombin. (I) Assessment of JON/A binding to resuspended mouse platelets isolated from calcitriol-treated and control WT mice (n = 5), after stimulation with 0.03 U/mL thrombin. (J) Representative images and quantitative analysis of FeCl_3_-induced thrombus formation in recipient mice (n = 10). Platelet-deprived recipient mice were infused with calcein-labeled platelets from calcitriol-treated or control WT mice before FeCl_3_-induced mesenteric artery injury. The area of thrombus and the total occlusion time were analyzed. Data were analyzed by the paired-samples *t*-test in C to E, 1-way analysis of variance with Sidak's multiple comparisons in F to I, and unpaired Student's *t*-test in (J). Data are presented as mean ± SD. ∗*P* < 0.05; ∗∗*P* < 0.01; ns, not significant. ADP = adenosine diphosphate; FeCl_3_ = ferric trichloride; MFI = mean fluorescence intensity; PRP = platelet-rich plasma; WT = wild-type.

To explore the in vivo effects of calcitriol on human platelet activation, we correlated platelet reactivity in platelet-rich plasma (PRP) with plasma calcitriol levels from 118 healthy participants (baseline characteristics in Supplemental Table 1). A negative correlation was observed between plasma calcitriol levels and platelet aggregation in response to 2 μM adenosine diphosphate (ADP) (Figure 1A) and 0.2 μg/mL collagen (Figure 1B) in PRP. Then, we conducted an intervention study involving 10 healthy participants. Baseline characteristics are detailed in Supplemental Table 2. Participants were instructed to take calcitriol (0.25 μg, twice daily) for 2 weeks. Plasma calcitriol concentrations and platelet activation markers were evaluated both before and subsequent to the administration of calcitriol. An increase in plasma calcitriol levels was observed after 2 weeks of calcitriol administration (Figure 1C). Calcitriol ingestion was shown to attenuate platelet aggregation triggered by ADP and collagen in PRP (Figures 1D and 1E). Furthermore, calcitriol ingestion was found to attenuate thrombin-induced P-selectin expression and integrin αIIbβ3 activation (Supplemental Figures 1A and 1B). In addition, we found a positive correlation between the increase in plasma calcitriol levels and the corresponding decrease in platelet activation in healthy participants (Supplemental Figures 2A to 2D). Collectively, these findings indicated that calcitriol supplementation reduced human platelet activation.

### Calcitriol administration inhibits the activity of mouse platelets

Next, we sought to investigate the effects of calcitriol ingestion on mouse platelet activation. WT mice were administered intragastrically with calcitriol (50 ng/kg per day) or the same volume of saline for 2 weeks. Calcitriol ingestion was observed to suppress platelet aggregation in PRP induced by both low and high concentrations of ADP or collagen (Figures 1F and 1G). In addition, washed platelets (WPs) from calcitriol-treated mice exhibited decreased platelet aggregation rates in response to 2 concentrations of thrombin, collagen, or ADP, and reduced adenosine triphosphate (ATP) release triggered by 2 concentrations of thrombin or collagen (Supplemental Figures 3A to 3C). Furthermore, thrombin-induced P-selectin expression and αIIbβ3 integrin activation on mouse platelets were consistently reduced in calcitriol-treated mice (Figures 1H and 1I). Calcitriol administration also attenuated mouse platelet spreading and clot retraction (Supplemental Figures 3D and 3E). To further validate the in vivo prothrombotic potential of calcitriol, we investigated thrombus formation in mesenteric arterioles of WT mice injured with FeCl_3_. Consequently, calcitriol supplementation reduced thrombus formation within mesenteric arterioles compared with those receiving saline ingestion (Figure 1J). Notably, tail bleeding time was similar between calcitriol-treated and vehicle-treated WT mice (Supplemental Figure 4), indicating minimal effects on baseline hemostasis.

### Calcitriol directly inhibits human platelet activation in vitro

**Figure 2:**
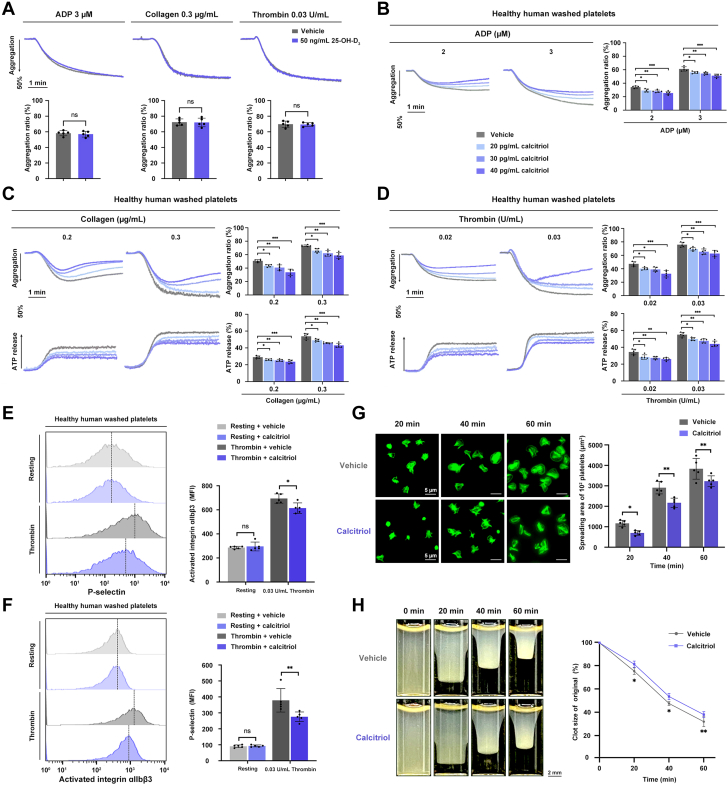
Calcitriol Exerts a Direct Inhibitory Effect on Human Platelet Function (A) Representative recordings and quantitative analysis of platelet aggregation in resuspended human platelets pretreated with 50 ng/mL 25-OH-D_3_ or vehicle, induced by ADP, collagen, or thrombin (n = 5). (B) Representative recordings and summary data of platelet aggregation in resuspended human platelets subjected to pretreatment with varying concentrations of calcitriol or vehicle, followed by stimulation with 2 different concentrations of ADP (n = 5). (C and D) Representative traces and summary data illustrating platelet aggregation and ATP release in resuspended human platelets treated with different concentrations of calcitriol or vehicle before stimulation with 2 distinct concentrations of collagen (C) or thrombin (D) (n = 5). (E) Evaluation of P-selectin surface expression on resuspended human platelets isolated from healthy volunteers, pretreated with 40 pg/mL calcitriol or vehicle, and subsequently stimulated with 0.03 U/mL thrombin (n = 5). (F) Assessment of PAC1 binding to resuspended human platelets isolated from healthy volunteers, pretreated with 40 pg/mL calcitriol or a vehicle control, and subsequently stimulated with 0.03 U/mL thrombin (n = 5). (G) Representative images and quantitative analysis of human platelet spreading on immobilized fibrinogen at different time points (n = 5), comparing platelets pretreated with calcitriol or a vehicle control. One hundred randomly selected platelets were measured for each condition. (H) Representative images and quantitative analysis of clot retraction in resuspended human platelets pretreated with calcitriol or a vehicle control at different time points (n = 5). Data were analyzed by unpaired Student's *t*-test in (A), 1-way ANOVA with Dunnett's multiple comparisons in B to D, 1-way ANOVA with Sidak's multiple comparisons in (E and F), and 2-way ANOVA with Tukey's multiple comparisons test in G and H. Data are presented as mean ± SD. ∗*P* < 0.05; ∗∗*P* < 0.01; ∗∗∗*P* < 0.001; ns, not significant. ADP = adenosine diphosphate; ANOVA = analysis of variance; ATP = adenosine triphosphate; MFI = mean fluorescence intensity.

Circulating vitamin D primarily exists in its inactive form 25(OH)D with normal levels between 20 and 50 ng/mL.^25^ Our findings indicated that 25(OH)D_3_ did not regulate washed human platelet aggregation triggered by collagen, thrombin, or ADP, even at a concentration of 50 ng/mL (Figure 2A).

Next, we sought to explore the direct effects of calcitriol on human platelet activation. A previous study calculated tertiles of 1,25(OH)_2_D_3_ based on the population distribution, with mean 1,25(OH)_2_D_3_ concentrations of 22.6, 33.2, and 46.6 pg/mL for the low, middle, and high tertiles, respectively.^26^ To reflect these physiological variations, we used 3 calcitriol concentrations (20, 30, and 40 pg/mL) representing low, middle, and high plasma 1,25(OH)_2_D_3_ levels in humans.

Calcitriol concentration-dependently inhibited platelet aggregation induced by collagen, thrombin, or ADP, as well as ATP release from dense granules stimulated by thrombin or collagen in human WPs. These effects were observed across 2 concentrations of each agonist (Figures 2B to 2D). In agonist-activated platelets, calcitriol reduced P-selectin release and platelet integrin αIIbβ3 activation (Figures 2E and 2F, Supplemental Figure 5A to 5D). In line with these inhibitory effects on αIIbβ3 activation, calcitriol also inhibited platelet spreading on fibrinogen and clot retraction in human platelet suspensions (Figures 2G and 2H). To further investigate the optimal calcitriol concentration for modulating platelet function, we conducted a titration study using concentrations ranging from 20 to 100 pg/mL. Increasing calcitriol concentrations progressively reduced human platelet aggregation, with a plateau observed above 60 pg/mL (Supplemental Figures 6A to 6C). The data collectively suggest that calcitriol directly inhibits platelet activation, and 60 pg/mL appears to be an optimal concentration for modulating human platelet function.

### Calcitriol binds to VDR in platelets to modulate platelet activation and in vivo thrombosis

We aimed to clarify the mechanisms of calcitriol-mediated platelet inhibition. Given that vitamin D exerts its effects via VDR binding,^27^ and VDR is present in platelet soluble and mitochondrial compartments,^28^ we examined whether calcitriol inhibited platelet activation through VDR binding. To this end, we used *Vdr*^*−/−*^ mice, as confirmed by both polymerase chain reaction and Western blotting (Supplemental Figures 7A and 7B). *Vdr* deficiency did not alter the number of α-granules or dense granules (Supplemental Figure 7C). Furthermore, there were no significant differences in platelet count and mean platelet volume between WT and *Vdr*^*−/−*^ mice (Supplemental Figure 7D). In addition, the VDR antagonist MeTC7 was also used to investigate the role of VDR in platelet activation.^29^

**Figure 3:**
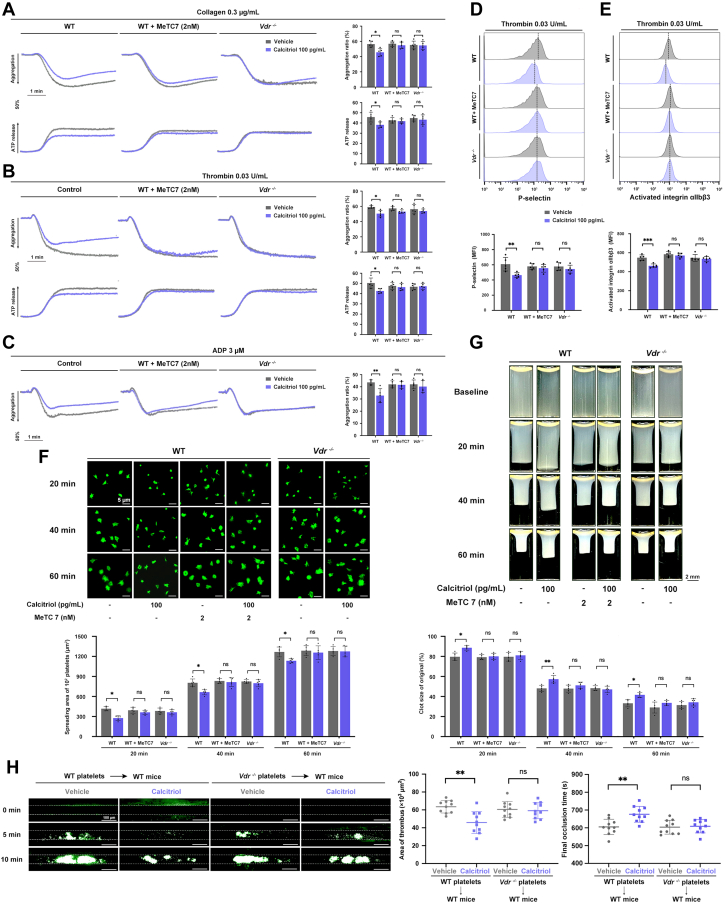
Calcitriol Engages With the VDR in Platelets to Inhibit Platelet Activation (A and B) Representative traces and quantitative analysis of platelet aggregation and ATP release in resuspended WT and *Vdr*^*−/−*^ mouse platelets subjected to different treatment conditions induced by collagen (A) or thrombin (B) (n = 5). (C) Representative recordings and quantitative analysis of platelet aggregation in resuspended WT and *Vdr*^*−/−*^ mouse platelets, comparing the effects of various treatment conditions induced by ADP (n = 5). (D) Evaluation of P-selectin surface expression on resuspended mouse platelets isolated from WT and *Vdr*^*−/−*^ mice that underwent different treatment conditions (n = 5), after stimulation with 0.03 U/mL thrombin. (E) JON/A binding to resuspended WT and *Vdr*^*−/−*^ mouse platelets stimulated with 0.03 U/mL thrombin after different treatments (n = 5). (F) Representative images and quantitative analysis of platelet spreading on immobilized fibrinogen at different time points (n = 5), comparing WT and *Vdr*^*−/−*^ mouse platelets subjected to various treatment conditions. One hundred randomly selected platelets were measured for each condition. (G) Representative images and quantitative analysis of clot retraction in resuspended WT and *Vdr*^*−/−*^ mouse platelets subjected to various treatment conditions (n = 5). (H) Representative images and quantitative analysis of FeCl_3_-induced thrombus formation in WT mice (n = 10). Platelet-deprived WT mice were infused with either pretreated WT or *Vdr*^*−/−*^ platelets before FeCl_3_-induced mesenteric artery injury. The area of thrombus and the final occlusion time were analyzed. Data were analyzed by 1-way ANOVA followed by Sidak's multiple comparisons in (A to E and H), and 2-way ANOVA with Tukey's multiple comparisons test in (F and G). Data are presented as mean ± SD. ∗*P* < 0.05; ∗∗*P* < 0.01; ∗∗∗*P* < 0.001; ns, not significant. ADP = adenosine diphosphate; ANOVA = analysis of variance; ATP = adenosine triphosphate; FeCl_3_ = ferric trichloride; MFI = mean fluorescence intensity; VDR = vitamin D receptor;WT = wild-type.

We performed a calcitriol titration study (40-200 pg/mL) in washed mouse platelets. Increasing calcitriol concentrations progressively inhibited platelet aggregation, which plateaued above 120 pg/mL (Supplemental Figures 8A to 8C). Consistent with its effects on human WPs, calcitriol attenuated agonist-induced platelet aggregation and ATP release in WPs from WT. These inhibitory effects were VDR-dependent, as evidenced by their attenuation in the presence of MeTC7 or in *Vdr*^*−/−*^ mice (Figures 3A to 3C). Furthermore, calcitriol reduced thrombin-induced P-selectin exposure (Figure 3D) and αIIbβ3 activation (Figure 3E) in WT platelets but not in MeTC7-pretreated WT platelets or *Vdr*^*−/−*^ platelets. Baseline P-selectin exposure and αIIbβ3 activation remained unchanged in all groups (Supplemental Figures 9A and 9B). In addition, calcitriol inhibited platelet spreading (Figure 3F) and clot retraction (Figure 3G) in WT platelets, while those effects diminished by MeTC7 or *Vdr*^*−/−*^ platelets. In vivo, platelet-deprived WT mice receiving calcitriol-treated WT platelets exhibited reduced thrombus formation compared with controls. Notably, this reduction was attenuated in mice receiving calcitriol-treated *Vdr*^*−/−*^ platelets (Figure 3H). These data collectively provide strong evidence that calcitriol inhibits platelet activation in both in vitro and in vivo models, primarily through its interaction with the VDR in platelets.

### Calcitriol downregulates platelet P2Y_12_ receptor expression

**Figure 4:**
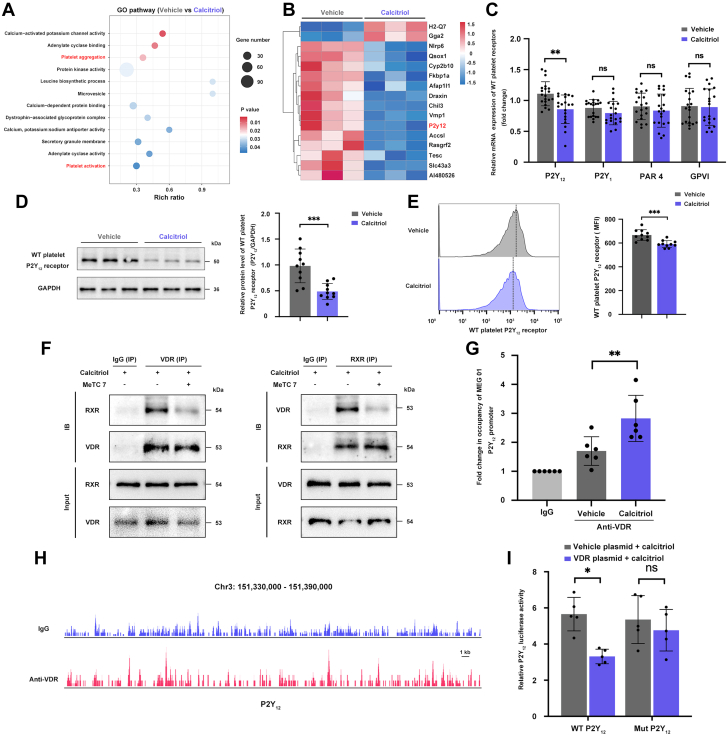
Calcitriol-Activated VDR Downregulates P2Y_12_ Receptor Expression (A) Identification of enriched gene ontology pathways associated with differentially expressed genes in platelets from WT mice after 2 weeks of calcitriol or vehicle administration. (B) Heatmap highlighting the most significantly enriched proteins. (C) Quantitative polymerase chain reaction analysis of mRNA expression levels for a panel of critical platelet activation receptors in WT mice subjected to 2 weeks of calcitriol or vehicle administration (n = 20). (D and E) Assessment of P2Y_12_ receptor protein expression in platelets isolated from WT mice after 2 weeks of calcitriol or vehicle administration, using Western blotting (D) (n = 10) and flow cytometry (E) (n = 10). (F) Coimmunoprecipitation of calcitriol-activated VDR with RXR in Meg-01 cells. Mouse anti-VDR and anti-RXR antibodies were used to immunoprecipitate the corresponding proteins from cell lysates derived from Meg-01 cells subjected to pretreatment with or without MeTC7. Mouse isotype IgG served as a negative control. Representative results from at least 3 independent experiments are shown. (G) Quantitative polymerase chain reaction analysis of VDR binding to the P2Y_12_ promoter in Meg-01 cells subjected to pretreatment with calcitriol or a vehicle control, after chromatin immunoprecipitation. Nonspecific IgG served as a negative control. Data were normalized to the preimmunoprecipitation input for each sample and expressed as the fold change relative to the control (n = 6). (H) Chromatin immunoprecipitation sequencing analysis of VDR occupancy at the P2Y_12_ promoter in calcitriol-treated Meg-01 cells. (I) Luciferase assays were performed in 293T cells cotransfected with either pFLAG-VDR and pGL3-P2Y_12_ WT promoter plasmids, or pFLAG-VDR and pGL3-P2Y_12_ mutant promoter plasmids. The pFLAG vector served as a control (n = 5). Cells were cultured in the presence of 40 pg/mL calcitriol. Data were analyzed by the nonparametric Mann-Whitney *U* test in the comparison of P2Y_12_ and P2Y_1_ in (C) and unpaired Student's *t*-test in the comparison of PAR4 and GPVI in C, as well as (D and E). Data were analyzed by 1-way analysis of variance with Sidak's multiple comparisons in (G and I). Data are presented as mean ± SD. ∗*P* < 0.05; ∗∗*P* < 0.01; ∗∗∗*P* < 0.001; ns, not significant. GPVI = glycoprotein VI; MFI = mean fluorescence intensity; PAR4 = protease-activated receptor 4; RXR = retinoid X receptor; VDR = vitamin D receptor; WT = wild-type.

On ligand binding, the VDR binds to specific DNA sequences within the promoter regions of various target genes, thereby regulating their expression.^30^ Our subsequent investigation focused on elucidating whether VDR exerts a regulatory effect on platelet activation by modulating associated gene expression. RNA sequencing was conducted on platelet samples obtained from WT mice administered calcitriol (50 ng/kg per day for a 2-week period) and a vehicle control. Gene ontology enrichment analysis revealed a significant over-representation of differentially expressed genes associated with platelet aggregation and platelet activation (Figure 4A). Moreover, the P2Y_12_ receptor, a pivotal mediator of platelet activation and thrombosis,^31^ exhibited a marked downregulation of gene expression after calcitriol intervention (Figure 4B), suggesting its potential as a therapeutic target for the inhibitory effects of calcitriol on platelet activation. To validate the observations from RNA sequencing, we quantified the messenger RNA (mRNA) expression levels of various critical platelet activation receptors. Quantitative polymerase chain reaction analysis demonstrated a specific downregulation of P2Y_12_ receptor mRNA expression by calcitriol, whereas the mRNA levels of other key platelet activation receptors, including P2Y_1_, protease-activated receptor 4, and glycoprotein VI, remained unchanged (Figure 4C). In addition, the downregulation of P2Y_12_ receptor expression was consistently observed at protein level, as confirmed by Western blotting and flow cytometry (Figures 4D and 4E).

### Calcitriol-activated VDR downregulates P2Y_12_ transcription in megakaryocytes

The regulatory effect of calcitriol on P2Y_12_ expression was investigated in Meg-01 cells. Calcitriol induced the formation of a VDR/retinoid X receptor (RXR) heterodimer, which is critical for VDR elements (VDRE)–mediated transcription.^30^ This interaction was demonstrated by coimmunoprecipitation and was effectively blocked by the VDR antagonist MeTC7 (Figure 4F). Although calcitriol did not alter the expression of the VDR at either the mRNA or protein levels (Supplemental Figure 10), chromatin immunoprecipitation analysis demonstrated the enrichment of the VDR at the P2Y_12_ promoter region in Meg-01 cells pretreated with calcitriol (Figure 4G). In addition, we performed chromatin immunoprecipitation sequencing analysis for VDR occupancy in the presence of calcitriol and observed that the preponderance of VDR binding occurred within the P2Y_12_ promoter region (Figure 4H). In megakaryocytes isolated from WT mice, we observed an enhancement of VDR enrichment within the P2Y_12_ promoter region after calcitriol ingestion (Supplemental Figure 11), recapitulating our findings from the Meg-01 cell line. Luciferase reporter assays further validated the inhibitory effect of the VDR on P2Y_12_ promoter activity. The ^1628^TAGGTTCA^1635^ region was identified as a critical motif for the binding of the VDR to the P2Y_12_ promoter, as mutations within this region eliminated the VDR-mediated reduction in P2Y_12_ receptor expression (Figure 4I). These observations collectively suggest that calcitriol-activated VDR exerts its genomic effects by downregulating P2Y_12_ receptor expression, thereby modulating platelet activation.

### Calcitriol regulates downstream signaling pathways of platelet VDR to decrease platelet activation

**Figure 5:**
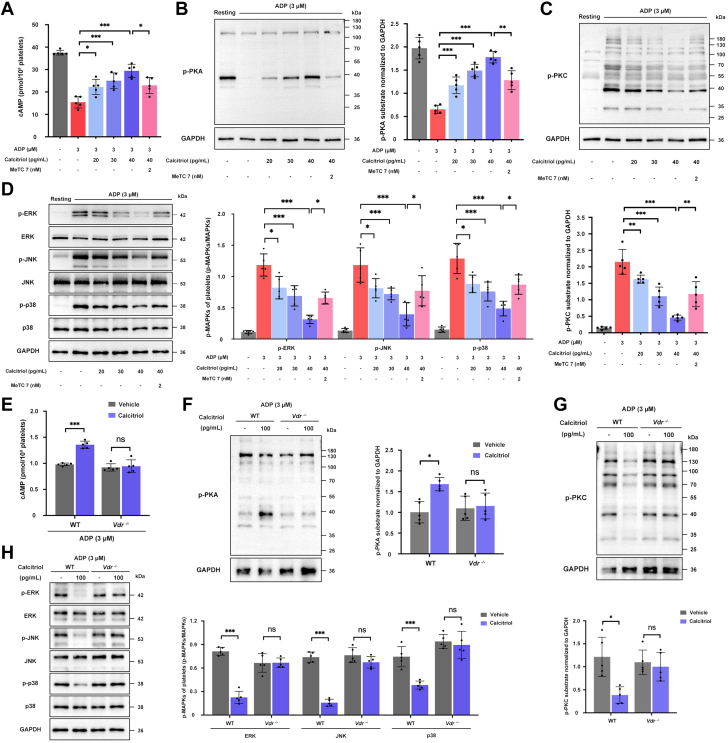
Calcitriol Regulates VDR Signaling in Platelets to Reduce Platelet Activation (A) ELISA-based quantification of cAMP levels in human platelets subjected to pretreatment with varying concentrations of calcitriol in the presence or absence of MeTC7, followed by stimulation with ADP. Summary data are presented (n = 5). (B and C) Detection of ADP-induced phosphorylation of PKA (B) and PKC (C) in human platelets using Western blotting, after pretreatment with various concentrations of calcitriol in the presence or absence of MeTC7. Representative immunoblotting results and summary data are presented (n = 5). (D) Assessment of ADP-induced ERK, JNK, and p38 phosphorylation in human platelets treated with varying calcitriol concentrations, with and without MeTC7. Representative immunoblotting results and summary data from 5 independent experiments are shown. (E) Quantification of intracellular cAMP levels in WT and *Vdr*^*−/−*^ mouse platelets, treated with calcitriol or vehicle control, followed by stimulation with ADP. Summary data from 5 independent experiments are presented. (F and G) Assessment of the effects of calcitriol on ADP-induced phosphorylation of PKA (F) and PKC (G) in WT and *Vdr*^*−/−*^ mouse platelets. Summary data from 5 independent experiments are presented. (H) Evaluation of ADP-induced phosphorylation of ERK, JNK, and p38 in WT and *Vdr*^*−/−*^ mouse platelets subjected to calcitriol or vehicle treatment. Representative immunoblotting results and summary data from 5 independent experiments are presented. Data were analyzed by 1-way ANOVA with Dunnett's multiple comparisons in (A to D) and 1-way ANOVA with Sidak's multiple comparisons in (E to H). Data are presented as mean ± SD. ∗*P* < 0.05; ∗∗*P* < 0.01; ∗∗∗*P* < 0.001; ns, not significant. ADP = adenosine diphosphate; ANOVA =analysis of variance; cAMP = cyclic adenosine monophosphate; ELISA = enzyme-linked immunoadsorbent assay; ERK1/2 = extracellular signal-regulated kinase 1/2; GAPDH = glyceraldehyde-3-phosphate dehydrogenase; JNK = c-Jun N-terminal kinase; MAPK = mitogen-activated protein kinase; PKA = protein kinase A; PKC = protein kinase C; VDR = vitamin D receptor; WT = wild-type.

Previous studies have demonstrated the involvement of the cAMP/PKA signaling pathway in the nongenomic actions of VDR in muscle and sertoli cells.^32^^,^^33^ Our study revealed that calcitriol concentration-dependently increased cAMP levels (Figure 5A) and potentiated ADP-induced phosphorylation of PKA in human platelets (Figure 5B). These effects were blunted by the VDR antagonist MeTC7 (Figures 5A and 5B). The cAMP/PKA signaling pathway suppresses the activation of protein kinase C (PKC), culminating in the inhibition of platelet activation and thrombosis.^34^, ^35^, ^36^ Consistent with this, calcitriol concentration-dependently attenuated PKC phosphorylation stimulated by ADP. These effects were also abrogated by MeTC7 (Figure 5C). Moreover, treating resting platelets with calcitriol led to elevated intracellular cAMP concentrations and enhanced PKA phosphorylation, but had limited effects on PKC phosphorylation (Supplemental Figures 12A to 12C). In addition, we investigated the MAPK pathway, which is known to be regulated by calcitriol^37^ and to play a well-established role in platelet activation and thrombosis.^38^ Calcitriol exhibited a concentration-dependent inhibition of the phosphorylation of extracellular signal-regulated kinase 1/2, c-Jun N-terminal kinase, and p38 in human platelets, all of which were abrogated by MeTC7 (Figure 5D).

Consistent with the findings in human platelets, calcitriol increased cAMP levels in WT platelets but not in *Vdr*^*−/−*^ platelets (Figure 5E). In addition, calcitriol increased the activity of PKA (Figure 5F), while reducing the phosphorylation of PKC, extracellular signal-regulated kinase 1/2, c-Jun N-terminal kinase, and p38 in ADP-activated WT platelets (Figures 5G and 5H). These effects were abolished in *Vdr*^*−/−*^ platelets (Figures 5E to 5H). Collectively, these findings demonstrate that calcitriol triggers the cAMP/PKA signaling pathway and inhibits the MAPK signaling pathway downstream of the platelet VDR.

### Calcitriol addresses platelet hyperactivity of patients with MASLD

**Figure 6:**
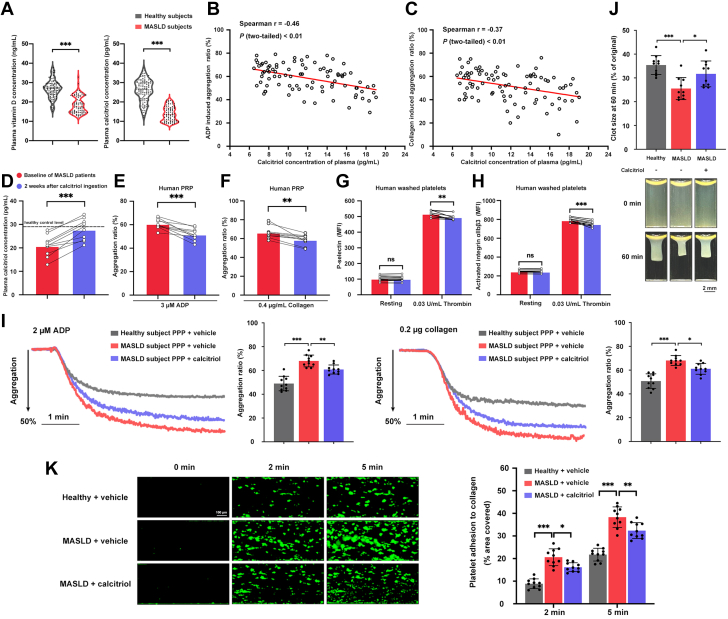
Calcitriol Mitigates Platelet Hyperactivity in Patients With MASLD (A) Plasma vitamin D and calcitriol levels were quantified in patients with MASLD and healthy participants. (B and C) Platelet aggregation induced by ADP (B) or collagen (C) in PRP from patients with MASLD was negatively correlated with plasma calcitriol levels (Spearman *r* = −0.46, *P* < 0.01; Spearman *r* = −0.37, *P* < 0.01; n = 89). Platelet aggregation was measured using the aggregometer. Each data point represents a single individual. (D) Plasma calcitriol levels in patients with MASLD before and after a 2-week calcitriol supplementation period (n = 10, 5 males). The dashed line represents the baseline calcitriol levels of the healthy group. (E and F) Platelet aggregation in PRP from patients with MASLD before and after calcitriol intervention in response to 3 μM ADP (E) or 0.4 μg/mL collagen (F) (n = 10, 5 males). Each data point represents the mean of 5 replicate platelet aggregation measurements. (G and H) P-selectin expression (G) and PAC1 binding (H) were assessed on human platelets stimulated with 0.03 U/mL thrombin from patients with MASLD (n = 10, 5 males) before and after calcitriol ingestion. (I) PPP from patients with MASLD was preincubated with either calcitriol or saline and then used to resuspend healthy platelets. PPP from healthy participants preincubated with saline served as a vehicle control. Platelet aggregation was triggered by 2 μM ADP (left panel) or 0.2 μg/mL collagen (right panel). Representative aggregation tracings and summary data are presented (n = 10). (J) PPP from patients with MASLD was preincubated with either calcitriol or saline and then added to healthy platelets. PPP from healthy participants preincubated with saline served as a vehicle control. Clot sizes were measured at 60 minutes. Representative results and summary data are presented (n = 10). (K) Mepacrine-labeled (100 μM) whole blood from patients with MASLD and healthy participants, pretreated with calcitriol or saline, was perfused through a microfluidic device under arterial flow conditions (40 dynes/cm^2^). The device was coated with fibrillar collagen (100 μg/mL), and thrombus formation was monitored in real time using fluorescence microscopy. Representative thrombus formation images were captured at 2 and 5 minutes (n = 10). Data were analyzed by the Mann-Whitney *U* test in A, the paired-samples *t*-test in (D to H), and 1-way analysis of variance with Dunnett's multiple comparisons in (I to K). Data are presented as mean ± SD. ∗*P* < 0.05; ∗∗*P* < 0.01; ∗∗∗*P* < 0.001; ns, not significant. ADP = adenosine diphosphate; MASLD = metabolic dysfunction–associated steatotic liver disease; MFI = mean fluorescence intensity; PPP = platelet-poor plasma; PRP = platelet-rich plasma.

MASLD is a significant cardiovascular risk factor,^15^ and its association with vitamin D deficiency is well documented.^39^ Our study consistently showed that compared with age- and gender-matched healthy participants, patients with MASLD exhibited reduced plasma vitamin D and calcitriol levels (Figure 6A). Their baseline characteristics are summarized in Supplemental Table 3. We then correlated platelet reactivity in PRP with plasma calcitriol levels from 89 patients with MASLD (baseline characteristics in Supplemental Table 4). Notably, plasma calcitriol levels showed a negative correlation with platelet aggregation induced by 2 μM ADP (Figure 6B) and 0.2 μg/mL collagen (Figure 6C). Based on baseline plasma calcitriol tertiles, 89 patients with MASLD were stratified into groups with mild (18.30 ± 1.57 pg/mL), moderate (13.51 ± 1.33 pg/mL), and severe (9.72 ± 0.72 pg/mL) calcitriol deficiency. The severity of calcitriol deficiency in patients with MASLD was related to increased platelet hyperactivation (Supplemental Figures 13A and 13B).

Furthermore, we conducted a calcitriol intervention study in 10 patients with MASLD to investigate the effects of calcitriol in vitamin D–deficient individuals. Baseline characteristics are detailed in Supplemental Table 5. Plasma calcitriol levels demonstrated an increase after 2 weeks of calcitriol administration (Figure 6D). Calcitriol administration resulted in an attenuation of platelet aggregation induced by ADP and collagen in PRP (Figures 6E and 6F) and a reduction in thrombin-stimulated P-selectin expression (Figure 6G) and integrin αIIbβ3 activation (Figure 6H). In addition, we observed a positive correlation between the increase in plasma calcitriol levels and the corresponding decrease in platelet activation in patients with MASLD (Supplemental Figures 14A to 14D). We also compared the effects of calcitriol on healthy and MASLD platelets by normalizing the reduction in platelet reactivity to the individual increase in plasma calcitriol levels. Although absolute changes in parameters such as platelet aggregation and P-selectin expression showed a trend toward greater inhibition in the MASLD group (without reaching statistical significance), the MASLD population exhibited a more pronounced reduction in activated integrin αIIbβ3 per unit of calcitriol increase compared with the healthy cohort (Supplemental Figure 15).

We subsequently investigated the effects of calcitriol on platelets from patients with MASLD. Compared with plasma from healthy controls, plasma from patients with MASLD enhanced platelet aggregation and thrombin-induced clot retraction. However, these enhancements were notably attenuated by calcitriol (Figures 6I and 6J). To simulate in vivo thrombogenesis, we used a microfluidic whole-blood perfusion assay. Throughout the perfusion period, blood from patients with MASLD exhibited an increase in thrombus size compared with blood from healthy participants. Notably, calcitriol treatment attenuated thrombus formation on immobilized collagen in patients with MASLD (Figure 6K). In summary, calcitriol negatively regulates platelet function in MASLD and may offer a therapy to reduce MASLD-related thrombotic risk.

### Calcitriol administration inhibits platelet activation and thrombosis in MASLD mice

**Figure 7:**
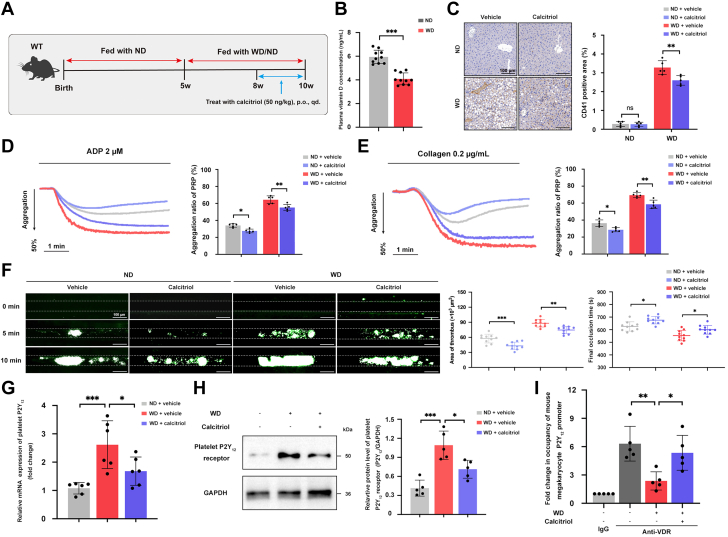
Calcitriol Suppresses Platelet Function and Thrombosis in WD-Induced MASLD Mice (A) A schematic diagram illustrating the construction of the MASLD mouse model and the experimental design for administering calcitriol to both MASLD and control WT mice (healthy mice). Five-week-old WT mice were fed a WD for 5 weeks to induce a MASLD model. Healthy mice received an ND for the same period. After 3 weeks on either WD or ND, calcitriol (50 ng/kg per day) was administered intragastrically for the remaining 2 weeks. (B) Quantification of plasma vitamin D levels in mice fed an ND or a WD. Summary data from 10 independent experiments are presented. (C) CD41 staining and quantification of intrahepatic platelets (CD41^+^) in mice fed ND or WD, treated with calcitriol or vehicle. Representative images and summary data are presented (n = 5). (D and E) Representative traces and quantitative analysis of platelet aggregation in platelet-rich plasma from mice fed an ND or a WD, treated with vehicle or calcitriol, and induced by ADP (D) or collagen (E) (n = 5). (F) Representative images and quantitative analysis of FeCl_3_-induced thrombus formation in mice fed an ND (n = 10). Platelet-deprived ND mice were subsequently infused with platelets from either calcitriol- or vehicle-treated ND or WD mice before FeCl_3_-induced mesenteric artery injury. (G) Quantitative polymerase chain reaction analysis of P2Y_12_ receptor mRNA expression in platelets isolated from ND and WD mice after 2 weeks of calcitriol or vehicle treatment (n = 5). (H) Western blotting analysis of platelet P2Y_12_ receptor protein expression in ND and WD mice after 2 weeks of calcitriol or vehicle treatment (n = 5). (I) Quantitative polymerase chain reaction analysis of VDR binding to the P2Y_12_ promoter in megakaryocytes isolated from calcitriol- or vehicle-treated ND and WD mice after chromatin immunoprecipitation. The isotype IgG of anti-VDR served as a negative control. Data were normalized to the preimmunoprecipitation input for each sample and expressed as the fold change relative to the control (n = 5). Data were analyzed by unpaired Student's *t*-test in B, 1-way ANOVA with Sidak's multiple comparisons in (C to F), and 1-way ANOVA with Dunnett's multiple comparisons in (G to I). Data are presented as mean ± SD. ∗*P* < 0.05; ∗∗*P* < 0.01; ∗∗∗*P* < 0.001; ns, not significant. ADP = adenosine diphosphate; ANOVA, analysis of variance; FeCl_3_ = ferric trichloride; GAPDH = glyceraldehyde-3-phosphate dehydrogenase; MASLD = metabolic dysfunction–associated steatotic liver disease; ND = normal diet; VDR = vitamin D receptor; WD = Western diet; WT = wild-type.

We established a mouse model of MASLD to investigate the potential role of vitamin D in protecting against thrombosis (Figure 7A). After 5 weeks of Western diet (WD) feeding, the MASLD model was successfully established (Supplemental Figure 16). A notable decrease in plasma vitamin D concentrations was observed in WD-induced MASLD mice compared with healthy mice (Figure 7B). In addition, MASLD mice exhibited an increase in intrahepatic platelet infiltration, which was attenuated by calcitriol treatment (Figure 7C). Compared with healthy mice, PRP from WD-induced MASLD mice exhibited enhanced platelet aggregation, which was attenuated by oral calcitriol gavage (Figures 7D and 7E). Calcitriol also reduced accelerated thrombus formation in the mesenteric arterioles of WD-induced MASLD mice in vivo (Figure 7F). In addition, calcitriol could augment the inhibitory effects of clopidogrel on platelet aggregation and thrombosis formation in MASLD (Supplemental Figures 17A and 17B), without increasing bleeding time (Supplemental Figure 17C).

We also used the methionine- and choline-deficient diet (MCDD)–induced MASLD model, which is advantageous for studying platelet function owing to its minimal effects on plasma lipids and glucose.^40^ Similar to WD-induced MASLD, vitamin D levels were decreased in MCDD-induced MASLD mice (Supplemental Figure 18). In addition, calcitriol could address potentiated platelet aggregation (Supplemental Figures 19A and 19B) and accelerated thrombus formation in mesenteric arterioles (Supplemental Figure 19C) observed in MCDD-induced MASLD mice. Our findings highlight the potential therapeutic benefits of calcitriol in addressing platelet hyperactivity associated with MASLD. A previous study showed that P2Y_12_ receptor expression was upregulated in the context of MASLD.^41^ Our findings corroborated this observation, demonstrating an overexpression of the P2Y_12_ receptor at both mRNA and protein levels in WD-induced MASLD mice compared with healthy mice. Calcitriol administration effectively reversed this overexpression (Figures 7G and 7H). Furthermore, MASLD mice exhibited reduced VDR enrichment in the P2Y_12_ promoter region of megakaryocytes, which was restored by calcitriol ingestion (Figure 7I). This study demonstrates that calcitriol effectively mitigates platelet hyperactivity in MASLD mice.

### Calcitriol attenuates thromboembolism and microvascular thrombosis in MASLD mice

**Figure 8:**
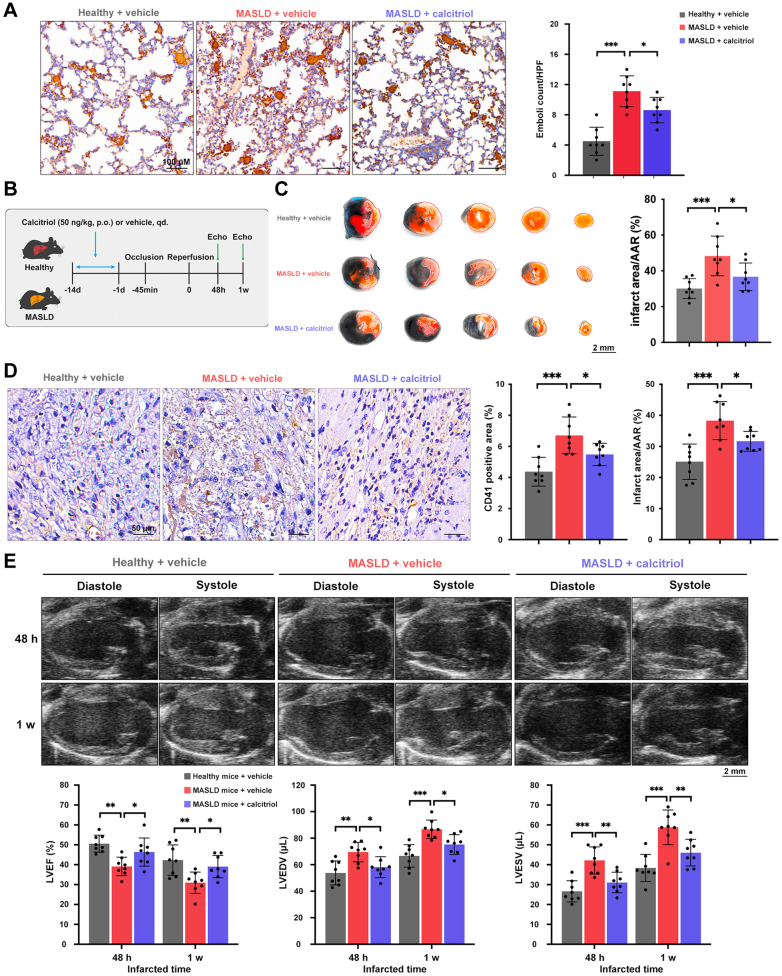
Calcitriol Prevents Thromboembolism and Microvascular Thrombosis in MASLD Mice (A) Representative immunohistochemistry images of lung tissue from MASLD and control WT mice (healthy mice) were examined in a collagen/epinephrine-induced pulmonary embolism model. Mice were supplemented with calcitriol (50 ng/kg) or a vehicle control for 2 weeks. Lung tissues were collected 10 minutes after collagen/epinephrine injection, and platelets were labeled with CD41. The number of lung emboli was quantified (n = 8). (B) Schematic protocol of calcitriol treatment and echocardiographic examination in a myocardial I/R injury study using MASLD and healthy mice. Before surgery, MASLD mice were administered calcitriol (50 ng/kg per day) for 2 weeks, with the final dose given 5 hours before occlusion. (C) Representative blue/TTC-stained left ventricular tissue sections from healthy and MASLD mice treated with either calcitriol or vehicle, after myocardial I/R injury. The blue areas indicate noninfarcted tissue, the white dashed lines delineate the infarct area (TTC-negative, pale region), and the red dashed lines indicate the AAR. The infarct area/AAR ratio was quantified. Representative results and summary data are provided (n = 8). (D) Representative immunohistochemistry images of myocardial tissue that underwent myocardial I/R injury from healthy and MASLD mice pretreated with vehicle or calcitriol. The area of intracardiac platelet positivity was quantified. Representative images and summary data are presented (n = 8). (E) Representative 2-dimensional echocardiographic images were obtained from vehicle- or calcitriol-pretreated healthy and MASLD mice after myocardial I/R injury. LVEF, LVEDV, and LVESV were quantified at 48 hours and 1 week postreperfusion. LVEF was determined using the biplane modified Simpson method. Representative images and summary data are presented (n = 8). Data were analyzed by 1-way analysis of variance with Dunnett's multiple comparisons in (A to C and E). Data are presented as mean ± SD. ∗*P* < 0.05; ∗∗*P* < 0.01; ∗∗∗*P* < 0.001. AAR = area at risk; HPF = high-power field; I/R = ischemia-reperfusion; LVEDV = left ventricular end-diastolic volume; LVEF = left ventricular ejection fraction; LVESV = left ventricular end-systolic volume; MASLD = metabolic dysfunction–associated steatotic liver disease; TTC = triphenyl tetrazolium chloride; WT = wild type.

To investigate the potential therapeutic effects of calcitriol in mitigating the thrombus propensity associated with MASLD and protecting vital organs from exacerbated tissue infarction, we used various thrombosis models. First, in a collagen/epinephrine-induced thromboembolism assay, MASLD mice exhibited more numerous pulmonary vascular thrombi compared with healthy controls. Calcitriol treatment reduced the number of pulmonary vascular thrombi in MASLD mice (Figure 8A). In addition, calcitriol does not affect fibrinolytic activity, as evidenced by an unchanged D-dimer concentration in this thromboembolism model (Supplemental Figure 20).

Next, we used a myocardial ischemia-reperfusion injury model (Figure 8B). Calcitriol treatment reduced the infarct area/area at risk ratio in MASLD mice (Figure 8C). Immunohistochemical analysis revealed an attenuation of ischemia-reperfusion–induced microvascular thrombosis in the reperfused myocardium of calcitriol-treated mice (Figure 8D). Moreover, cardiac function was evaluated via the biplane modified Simpson method^42^ (Supplemental Figure 21) and M-mode echocardiography. Calcitriol enhanced cardiac function, as evidenced by an elevated left ventricular ejection fraction and improved left ventricular volume parameters (Figure 8E, Supplemental Figure 22). Furthermore, we found positive correlations between microvascular thrombosis and infarct size (Supplemental Figure 23A), as well as inverse correlations with left ventricular ejection fraction (Supplemental Figure 23B) in MASLD mice.

Finally, we conducted a middle cerebral artery occlusion model. Calcitriol treatment effectively reduced infarct volumes and microvascular thrombosis in the brains of MASLD mice (Supplemental Figures 24A and 24B). In addition, a positive correlation existed between intracerebral platelet positivity area and brain infarct size in MASLD mice (Supplemental Figure 24C). In summary, calcitriol mitigated thromboembolism and microvascular thrombosis in MASLD mice, safeguarding vital organs from severe infarction.

## Discussion

Our findings reveal that 1) calcitriol inhibits platelet activation and in vivo thrombosis; 2) calcitriol-activated VDR in megakaryocytes downregulates P2Y_12_ receptor transcription and decreases platelet activation; 3) calcitriol binds to platelet VDR, activating cAMP/PKA signaling and inhibiting MAPK signaling to regulate platelet function; and 4) calcitriol ameliorates MASLD-associated platelet hyperreactivity, thereby suppressing in vivo thrombus formation and protecting key organs from ischemic infarction.

The vitamin D axis is a complex system regulated at multiple levels and interconnected with numerous cardiovascular signaling pathways.^43^ Vitamin D deficiency is a widespread condition, affecting approximately 30% to 60% of the global population.^44^^,^^45^ In Western countries, this deficiency is notably prevalent, with significant associations with established CVD risk factors^46^, ^47^, ^48^, ^49^ and a substantial increase in the occurrence of ischemic cardiovascular events, including MI, IS, peripheral artery disease, and sudden cardiac death.^1^^,^^50^ A meta-analysis of 19 prospective studies revealed that when 25(OH)D levels fell below approximately 24 ng/mL, the risk of CVD increased steadily with decreasing 25(OH)D levels, with a relative risk of 1.03 for every 10 ng/mL decline in 25(OH)D levels.^51^ A prospective study demonstrated that low levels of 25(OH)D are independently predictive of MI in men over a 10-year follow-up period.^7^ Another prospective study found that lower 25(OH)D levels were associated with an increased risk of IS in women.^8^ All of these clues collectively suggest that low 25(OH)D levels are a strong indicator of elevated thrombotic cardiovascular risk, and that circulating vitamin D may directly affect platelet function. Consistent with previous findings,^52^, ^53^, ^54^ our study demonstrated that calcitriol, an activated form of vitamin D, concentration-dependently attenuated agonist-induced platelet aggregation, granule release, integrin αIIbβ3 activation, platelet spreading, and clot retraction. Our data offer a theoretical rationale for the observed increase in ischemic events associated with vitamin D deficiency, suggesting that optimized calcitriol supplementation could prevent thrombotic events in deficient individuals.

Vitamin D exerts its biological effects by binding to the virtually ubiquitous VDR.^55^ Although the presence of VDR within the mitochondrial compartment of human platelets has been documented,^28^ and the VDR signaling pathway is well characterized in monocytes and vascular cells,^56^^,^^57^ the precise role of VDR in platelets has remained largely a mystery. Our research builds on previous findings by demonstrating a direct interaction between calcitriol and the platelet VDR. Significantly, the inhibitory effects of calcitriol on platelet activation were reversed by a VDR antagonist and in VDR-deficient mice, strongly suggesting a VDR-dependent mechanism.

VDR is a member of the nuclear receptor superfamily. It forms a heterodimer with the RXR when activated by calcitriol. This heterodimer acts as a transcription factor, binding to specific VDREs located within the promoter regions of target genes, and regulates approximately 3% of the genome.^55^^,^^58^ The genomic effects of VDR were reported to have protective effects on cardiovascular system. VDR was found to reverse age-related hypertension via downregulating the renal AP1/AT_1_R pathway^59^ and upregulate PTPN6 expression in macrophages to inhibit foam cell formation and induce autophagy.^60^ We determined the genomic mechanism by which VDR regulates platelet activation, revealing that calcitriol-activated VDR, on forming a heterodimer with RXR, directly binds to the VDRE in the P2Y_12_ gene promoter in megakaryocytes. This interaction leads to decreased P2Y_12_ receptor expression in megakaryocytes and subsequently reduced P2Y_12_ receptor levels on circulating platelets. The P2Y_12_ receptor plays a central role in platelet activation and thrombosis;^31^ our findings therefore hold significant potential for clinical translation. In addition, the observed disparity between the reduction in total P2Y_12_ protein and its surface expression highlights the complex dynamics of platelet receptor trafficking. Although genomic modulation of P2Y_12_ transcription mediated by the VDR-RXR complex substantially depletes intracellular reservoirs such as ɑ-granules or the open canalicular system, platelets may prioritize the maintenance of a functional surface pool to preserve hemostatic capacity. This prioritization is potentially sustained through active receptor recycling mechanisms, explaining why total protein levels exhibit a more pronounced decline compared with surface expression. Given that the 14-day calcitriol treatment encompasses the full turnover cycle of the circulating platelet population, these findings confirm that genomic regulation at the megakaryocyte level effectively translates into a sustained and fine-tuned functional deficit in mature platelets.

Given their anucleate nature and rapid response to calcitriol, platelets primarily manifest nongenomic effects mediated via the VDR.^61^ Our findings demonstrate that calcitriol potentiates the cAMP/PKA axis while antagonizing MAPK signaling downstream of the platelet VDR, directly attenuating activation. Within the hierarchy of platelet signaling, the cAMP/PKA axis functions as a negative regulator that elevates the activation threshold across multiple cascades. This mechanism elucidates how calcitriol exerts a broad-spectrum inhibitory effect, simultaneously dampening GPVI-, P2Y_12_-, and PAR-mediated responses to their respective ligands. Rather than targeting a single receptor, calcitriol reinforces the intrinsic homeostatic brake of the platelet. These nongenomic pathways operate as immediate biochemical modulators, rapidly shifting the platelet's sensitivity. Complementing this, the genomic effect (driven by VDR activity in megakaryocytes) provides a structural recalibration by reducing the surface density of P2Y_12_ receptors. This establishes a lower baseline of reactivity, whereby nongenomic signaling exerts dynamic, real-time fine-tuning.

MASLD is a significant risk factor for major cardiovascular events and associated complications.^18^^,^^62^^,^^63^ A substantial body of research supports the association between vitamin D deficiency and the progression of MASLD, independent of other risk factors.^64^ Although the causal relationship between vitamin D deficiency and MASLD remains difficult to definitively prove, it can be partly attributed to reduced capacity for vitamin D synthesis by the liver.^43^ Our findings consistently demonstrated lower vitamin D and calcitriol levels in the context of MASLD and further revealed the reversed association between plasma calcitriol levels and platelet aggregation in patients with MASLD. Importantly, calcitriol intervention reduced platelet hyperactivity and thrombus formation in both MASLD mice and patients, preventing severe infarction in vital organs. Calcitriol is anticipated to exert a more potent antithrombotic effect in vivo than indicated by in vitro assays, as it orchestrates both immediate signaling cascades and sustained transcriptional remodeling of the prothrombotic phenotype. Therefore, the biological significance of calcitriol in vivo likely stems from a synergistic dual layer mechanism. The genomic effect in megakaryocytes ensures a chronically suppressed state of reactivity, whereas the nongenomic effects in circulating platelets provide an adaptive response to acute metabolic or inflammatory surges. By addressing the etiology of vitamin D–dependent platelet dysfunction, calcitriol normalizes reactivity without compromising hemostasis or exacerbating the bleeding tendencies inherent in liver disease. Consequently, calcitriol intervention emerges as a promising targeted strategy to mitigate thrombotic complications in the MASLD population.

The efficacy of vitamin D supplementation in CVD prevention remains debated. Initial analysis of the D-Health cohort suggested that vitamin D supplementation did not reduce all-cause mortality from CVD.^65^ However, the effect on major cardiovascular events remained unexplored. A subsequent re-evaluation of the D-Health data, examining monthly 60,000 IU vitamin D supplementation in Australians aged 60 years and older, indicated a potential reduction in major cardiovascular events, particularly MI and coronary revascularization.^66^ Conversely, large-scale studies such as Vitamin D and Omega-3 Trial and Vitamin D Assessment Study have consistently shown no significant reduction in cardiovascular event risk with vitamin D supplementation.^4^^,^^5^ Although Vitamin D and Omega-3 Trial included a more racially diverse cohort and used annual surveys for participant-reported events,^4^ this methodology may have introduced reporting bias, potentially obscuring a protective effect of vitamin D.^66^ In addition, the hazard ratio of the Vitamin D Assessment Study study for MI was similar to the D-Health Trial findings, although the confidence interval was wide.^5^ This study demonstrated that calcitriol ingestion increased plasma calcitriol levels and reduced platelet aggregation, degranulation, and integrin αIIbβ3 activation in both healthy volunteers and patients with MASLD. Our findings suggest that patients with MASLD and individuals with vitamin D insufficiency (20-10 ng/mL) or deficiency (<10 ng/mL) are potential suitable candidates for calcitriol therapy. Although 0.25 μg twice daily may be sufficient for healthy individuals, we recommend regular monitoring of plasma calcitriol concentrations and dose adjustments to achieve a target of 60 pg/mL for optimal platelet function. Furthermore, close monitoring of renal function, plasma calcium, electrocardiogram, and clinical signs of vitamin D toxicity is essential.^67^ The efficacy of calcitriol administration in vitamin D–insufficient individuals, notably those with MASLD, warrants further verification through randomized controlled trials.

Our study has 3 limitations: First, although VDR downregulates nuclear factor-κB,^68^ a transcription factor that increases platelet P2Y_12_ receptor expression in DM,^69^ it remains unclear whether VDR-mediated P2Y_12_ downregulation is partly due to reduced nuclear factor-κB Secondly, the potential contributions of coagulation factors,^70^ endothelial dysfunction,^71^ plasma proteins,^72^ and inflammation^73^^,^^74^ to platelet hyperreactivity in patients with MASLD cannot be excluded. Finally, excluding MASLD individuals with preexisting CVD may limit the generalizability of our findings to the broader MASLD population.

## Conclusions

This study unveils a previously unrecognized mechanism by which calcitriol regulates platelet function and thrombotic risk. Calcitriol reduces platelet activation and thrombosis by targeting the VDR in megakaryocytes and platelets, involving P2Y_12_ downregulation and cAMP/PKA activation alongside MAPK inhibition. Our results indicate that calcitriol supplementation may offer a valuable therapeutic strategy for MASLD-associated thrombophilia, thereby protecting against infarction in critical organs.

### Data Availability

The data that support the findings of this study are available from the corresponding author on reasonable request.

## Funding Support and Author Disclosures

This work was supported by the National Natural Science Foundation of China (82570397 to Dr Qi, 82300375 to Dr Zhao, 82570396 to Dr Wu, and 82400156 to Dr Xu), Beijing, China, and the Fudan University Zhongshan Hospital's Science Foundation (2025ZSRC-005 to Dr Qi), Shanghai, China. The authors have reported that they have no relationships relevant to the contents of this paper to disclose.

Perspectives **COMPETENCY IN MEDICAL KNOWLEDGE:** Although vitamin D deficiency is associated with increased thrombotic risk, the direct impact of vitamin D on platelet function and its therapeutic potential in conditions with both vitamin D deficiency and platelet hyperreactivity, such as metabolic dysfunction–associated steatotic liver disease, are not fully understood.

**TRANSLATIONAL OUTLOOK:** Calcitriol negatively regulates platelet activity through vitamin D receptor activation in megakaryocytes and platelets. By downregulating P2Y12 expression and modulating cyclic adenosine monophosphate/protein kinase A and mitogen-activated protein kinase signaling, calcitriol offers therapeutic potential for reducing thrombotic complications in metabolic dysfunction–associated steatotic liver disease patients.

## References

[1] Pilz S., Verheyen N., Grübler M.R., Tomaschitz A., März W. (2016). Vitamin D and cardiovascular disease prevention. Nat Rev Cardiol.

[2] Hossein-nezhad A., Holick M.F. (2013). Vitamin D for health: a global perspective. Mayo Clin Proc.

[3] Ruiz-García A., Pallarés-Carratalá V., Turégano-Yedro M. (2023). Vitamin D supplementation and its impact on mortality and cardiovascular outcomes: systematic review and meta-analysis of 80 randomized clinical trials. Nutrients.

[4] Manson J.E., Cook N.R., Lee I.M. (2019). Vitamin D supplements and prevention of cancer and cardiovascular disease. N Engl J Med.

[5] Scragg R., Stewart A.W., Waayer D. (2017). Effect of monthly high-dose vitamin D supplementation on cardiovascular disease in the vitamin D assessment study: a randomized clinical trial. JAMA Cardiol.

[6] Brøndum-Jacobsen P., Benn M., Jensen G.B., Nordestgaard B.G. (2012). 25-hydroxyvitamin d levels and risk of ischemic heart disease, myocardial infarction, and early death: population-based study and meta-analyses of 18 and 17 studies. Arterioscler Thromb Vasc Biol.

[7] Giovannucci E., Liu Y., Hollis B.W., Rimm E.B. (2008). 25-hydroxyvitamin D and risk of myocardial infarction in men: a prospective study. Arch Intern Med.

[8] Sun Q., Pan A., Hu F.B., Manson J.E., Rexrode K.M. (2012). 25-Hydroxyvitamin D levels and the risk of stroke: a prospective study and meta-analysis. Stroke.

[9] Pilz S., Tomaschitz A., Drechsler C., Zittermann A., Dekker J.M., März W. (2011). Vitamin D supplementation: a promising approach for the prevention and treatment of strokes. Curr Drug Targets.

[10] Nsengiyumva V., Fernando M.E., Moxon J.V. (2015). The association of circulating 25-hydroxyvitamin D concentration with peripheral arterial disease: a meta-analysis of observational studies. Atherosclerosis.

[11] Gurbel P.A., Jeong Y.H., Navarese E.P., Tantry U.S. (2016). Platelet-mediated thrombosis: from bench to bedside. Circ Res.

[12] Korzonek-Szlacheta I., Hudzik B., Nowak J. (2018). Mean platelet volume is associated with serum 25-hydroxyvitamin D concentrations in patients with stable coronary artery disease. Heart Vessels.

[13] Aleva F.E., Tunjungputri R.N., van der Vorm L.N. (2020). Platelet integrin αIIbβ3 activation is associated with 25-Hydroxyvitamin D concentrations in healthy adults. Thromb Haemost.

[14] Le M.H., Yeo Y.H., Li X. (2022). 2019 global NAFLD prevalence: a systematic review and meta-analysis. Clin Gastroenterol Hepatol.

[15] Targher G., Valenti L., Byrne C.D. (2025). Metabolic dysfunction-associated steatotic liver disease. N Engl J Med.

[16] Targher G., Day C.P., Bonora E. (2010). Risk of cardiovascular disease in patients with nonalcoholic fatty liver disease. N Engl J Med.

[17] Targher G., Byrne C.D., Tilg H. (2020). NAFLD and increased risk of cardiovascular disease: clinical associations, pathophysiological mechanisms and pharmacological implications. Gut.

[18] Chew N.W.S., Mehta A., Goh R.S.J. (2025). Cardiovascular-liver-metabolic health: recommendations in screening, diagnosis, and management of metabolic dysfunction-associated steatotic liver disease in cardiovascular disease via modified Delphi approach. Circulation.

[19] Lee H.H., Lee H.A., Kim E.J. (2024). Metabolic dysfunction-associated steatotic liver disease and risk of cardiovascular disease. Gut.

[20] Guo X.F., Wang C., Yang T., Li S., Li K.L., Li D. (2020). Vitamin D and non-alcoholic fatty liver disease: a meta-analysis of randomized controlled trials. Food Funct.

[21] Tabrizi R., Moosazadeh M., Lankarani K.B. (2017). The effects of vitamin D supplementation on metabolic profiles and liver function in patients with non-alcoholic fatty liver disease: a systematic review and meta-analysis of randomized controlled trials. Diabetes Metab Syndr.

[22] Mansour-Ghanaei F., Pourmasoumi M., Hadi A., Ramezani-Jolfaie N., Joukar F. (2020). The efficacy of vitamin D supplementation against nonalcoholic fatty liver disease: a meta-analysis. J Diet Suppl.

[23] Kasper P., Martin A., Lang S. (2021). NAFLD and cardiovascular diseases: a clinical review. Clin Res Cardiol.

[24] Malladi N., Alam M.J., Maulik S.K., Banerjee S.K. (2023). The role of platelets in non-alcoholic fatty liver disease: from pathophysiology to therapeutics. Prostaglandins Other Lipid Mediat.

[25] Ross A.C. (2011). The 2011 report on dietary reference intakes for calcium and vitamin D. Public Health Nutr.

[26] Bea J.W., Jurutka P.W., Hibler E.A. (2015). Concentrations of the vitamin D metabolite 1,25(OH)_2_D and odds of metabolic syndrome and its components. Metabolism.

[27] Evans R.M., Mangelsdorf D.J. (2014). Nuclear receptors, RXR, and the big bang. Cell.

[28] Silvagno F., De Vivo E., Attanasio A., Gallo V., Mazzucco G., Pescarmona G. (2010). Mitochondrial localization of vitamin D receptor in human platelets and differentiated megakaryocytes. PLoS One.

[29] Khazan N., Kim K.K., Hansen J.N. (2022). Identification of a Vitamin-D receptor antagonist, MeTC7, which inhibits the growth of xenograft and transgenic tumors *in vivo*. J Med Chem.

[30] Carlberg C. (2022). Vitamin D and its target genes. Nutrients.

[31] Dorsam R.T., Kunapuli S.P. (2004). Central role of the P2Y_12_ receptor in platelet activation. J Clin Invest.

[32] Boland R.L. (2011). VDR activation of intracellular signaling pathways in skeletal muscle. Mol Cell Endocrinol.

[33] Menegaz D., Barrientos-Duran A., Kline A. (2010). 1alpha,25(OH)_2_-Vitamin D_3_ stimulation of secretion via chloride channel activation in sertoli cells. J Steroid Biochem Mol Biol.

[34] Aburima A., Wraith K.S., Raslan Z., Law R., Magwenzi S., Naseem K.M. (2013). cAMP signaling regulates platelet myosin light chain (MLC) phosphorylation and shape change through targeting the RhoA-Rho kinase-MLC phosphatase signaling pathway. Blood.

[35] Qi Z., Zhang W., Zhang P. (2025). The gut microbiota-bile acid-TGR5 axis orchestrates platelet activation and atherothrombosis. Nat Cardiovasc Res.

[36] Liu X., Zhang P., Lian Z. (2025). CCL21 enhances platelet activation and atherothrombosis via CCR7 activation. Circ Res.

[37] Hii C.S., Ferrante A. (2016). The non-genomic actions of vitamin D. Nutrients.

[38] Estevez B., Du X. (2017). New concepts and mechanisms of platelet activation signaling. Physiology (Bethesda).

[39] Liu J., Song Y., Wang Y., Hong H. (2023). Vitamin D/vitamin D receptor pathway in non-alcoholic fatty liver disease. Expert Opin Ther Targets.

[40] Jin K., Shi Y., Zhang H. (2023). TNFα/Miz1-positive feedback loop inhibits mitophagy in hepatocytes and propagates non-alcoholic steatohepatitis. J Hepatol.

[41] Malehmir M., Pfister D., Gallage S. (2019). Platelet GPIbα is a mediator and potential interventional target for NASH and subsequent liver cancer. Nat Med.

[42] Heinen A., Raupach A., Behmenburg F. (2018). Echocardiographic analysis of cardiac function after infarction in mice: validation of single-plane long-axis view measurements and the bi-plane simpson method. Ultrasound Med Biol.

[43] Carbone F., Liberale L., Libby P., Montecucco F. (2023). Vitamin D in atherosclerosis and cardiovascular events. Eur Heart J.

[44] Amrein K., Scherkl M., Hoffmann M. (2020). Vitamin D deficiency 2.0: an update on the current status worldwide. Eur J Clin Nutr.

[45] Holick M.F. (2007). Vitamin D deficiency. N Engl J Med.

[46] Trimarco V., Manzi M.V., Mancusi C. (2022). Insulin resistance and vitamin D deficiency: a link beyond the appearances. Front Cardiovasc Med.

[47] Rafiq S., Jeppesen P.B. (2021). Insulin resistance is inversely associated with the status of vitamin D in both diabetic and non-diabetic populations. Nutrients.

[48] Carbone F., Mach F., Vuilleumier N., Montecucco F. (2014). Potential pathophysiological role for the vitamin D deficiency in essential hypertension. World J Cardiol.

[49] Surdu A.M., Pînzariu O., Ciobanu D.M. (2021). Vitamin D and its role in the lipid metabolism and the development of atherosclerosis. Biomedicines.

[50] Michos E.D., Cainzos-Achirica M., Heravi A.S., Appel L.J. (2021). Vitamin D, calcium supplements, and implications for cardiovascular health: JACC focus seminar. J Am Coll Cardiol.

[51] Wang L., Song Y., Manson J.E. (2012). Circulating 25-hydroxy-vitamin D and risk of cardiovascular disease: a meta-analysis of prospective studies. Circ Cardiovasc Qual Outcomes.

[52] Nie S., Huang P., Niu H. (2025). Vitamin D deficiency enhances platelet activation and thrombosis by regulating VDR/Akt pathway based on platelet proteomics. Eur J Pharmacol.

[53] Johny E., Jala A., Nath B. (2022). Vitamin D supplementation modulates platelet-mediated inflammation in subjects with type 2 diabetes: a randomized, double-blind, placebo-controlled trial. Front Immunol.

[54] Sultan M., Twito O., Tohami T., Ramati E., Neumark E., Rashid G. (2019). Vitamin D diminishes the high platelet aggregation of type 2 diabetes mellitus patients. Platelets.

[55] Christakos S., Dhawan P., Verstuyf A., Verlinden L., Carmeliet G. (2016). Vitamin D: metabolism, molecular mechanism of action, and pleiotropic effects. Physiol Rev.

[56] Zhang Y., Leung D.Y., Richers B.N. (2012). Vitamin D inhibits monocyte/macrophage proinflammatory cytokine production by targeting MAPK phosphatase-1. J Immunol.

[57] Ni W., Watts S.W., Ng M., Chen S., Glenn D.J., Gardner D.G. (2014). Elimination of vitamin D receptor in vascular endothelial cells alters vascular function. Hypertension.

[58] Norman P.E., Powell J.T. (2014). Vitamin D and cardiovascular disease. Circ Res.

[59] Hua R., Liu B., He W. (2023). Calcitriol reverses age-related hypertension via downregulating renal AP1/AT_1_R pathway through regulating mitochondrial function. Clin Exp Hypertens.

[60] Kumar S., Nanduri R., Bhagyaraj E. (2021). Vitamin D_3_-VDR-PTPN6 axis mediated autophagy contributes to the inhibition of macrophage foam cell formation. Autophagy.

[61] Unsworth A.J., Flora G.D., Gibbins J.M. (2018). Non-genomic effects of nuclear receptors: insights from the anucleate platelet. Cardiovasc Res.

[62] Tsutsumi T., Eslam M., Kawaguchi T. (2021). MAFLD better predicts the progression of atherosclerotic cardiovascular risk than NAFLD: generalized estimating equation approach. Hepatol Res.

[63] Lonardo A., Sookoian S., Pirola C.J., Targher G. (2016). Non-alcoholic fatty liver disease and risk of cardiovascular disease. Metabolism.

[64] Zhang Z., Moon R., Thorne J.L., Moore J.B. (2023). NAFLD and vitamin D: evidence for intersection of microRNA-regulated pathways. Nutr Res Rev.

[65] Neale R.E., Baxter C., Romero B.D. (2022). The D-Health trial: a randomised controlled trial of the effect of vitamin D on mortality. Lancet Diabetes Endocrinol.

[66] Thompson B., Waterhouse M., English D.R. (2023). Vitamin D supplementation and major cardiovascular events: D-health randomised controlled trial. BMJ.

[67] Marcinowska-Suchowierska E., Kupisz-Urbańska M., Łukaszkiewicz J., Płudowski P., Jones G. (2018). Vitamin D toxicity-a clinical perspective. Front Endocrinol.

[68] Yu X.P., Bellido T., Manolagas S.C. (1995). Down-regulation of NF-kappa B protein levels in activated human lymphocytes by 1,25-dihydroxyvitamin D_3_. Proc Natl Acad Sci USA.

[69] Hu L., Chang L., Zhang Y. (2017). Platelets express activated P2Y_12_ receptor in patients with diabetes mellitus. Circulation.

[70] Kotronen A., Joutsi-Korhonen L., Sevastianova K. (2011). Increased coagulation factor VIII, IX, XI and XII activities in non-alcoholic fatty liver disease. Liver Int.

[71] Stahl E.P., Dhindsa D.S., Lee S.K., Sandesara P.B., Chalasani N.P., Sperling L.S. (2019). Nonalcoholic fatty liver disease and the heart: JACC state-of-the-art review. J Am Coll Cardiol.

[72] Zhang P., Qi Z., Xu H. (2025). Fetuin-A increases thrombosis risk in non-alcoholic fatty liver disease by binding to TLR-4 on platelets. Cardiovasc Res.

[73] Francque S.M., Van Der Graaff D., Kwanten W.J. (2016). Non-alcoholic fatty liver disease and cardiovascular risk: pathophysiological mechanisms and implications. J Hepatol.

[74] Simon T.G., Trejo M.E.P., McClelland R. (2018). Circulating Interleukin-6 is a biomarker for coronary atherosclerosis in nonalcoholic fatty liver disease: results from the multi-ethnic study of atherosclerosis. Int J Cardiol.

