# Supplementary material for: Calcitriol Suppresses Platelet Activation and Thrombosis, Mitigating Cardiovascular Risks in Metabolic Dysfunction–Associated Steatotic Liver Disease
Source: JACC Basic Transl Sci. 2026 Jul 10;11(8):101632. doi: 10.1016/j.jacbts.2026.101632 (PMC13380722; doi:10.1016/j.jacbts.2026.101632)

Figure 4D

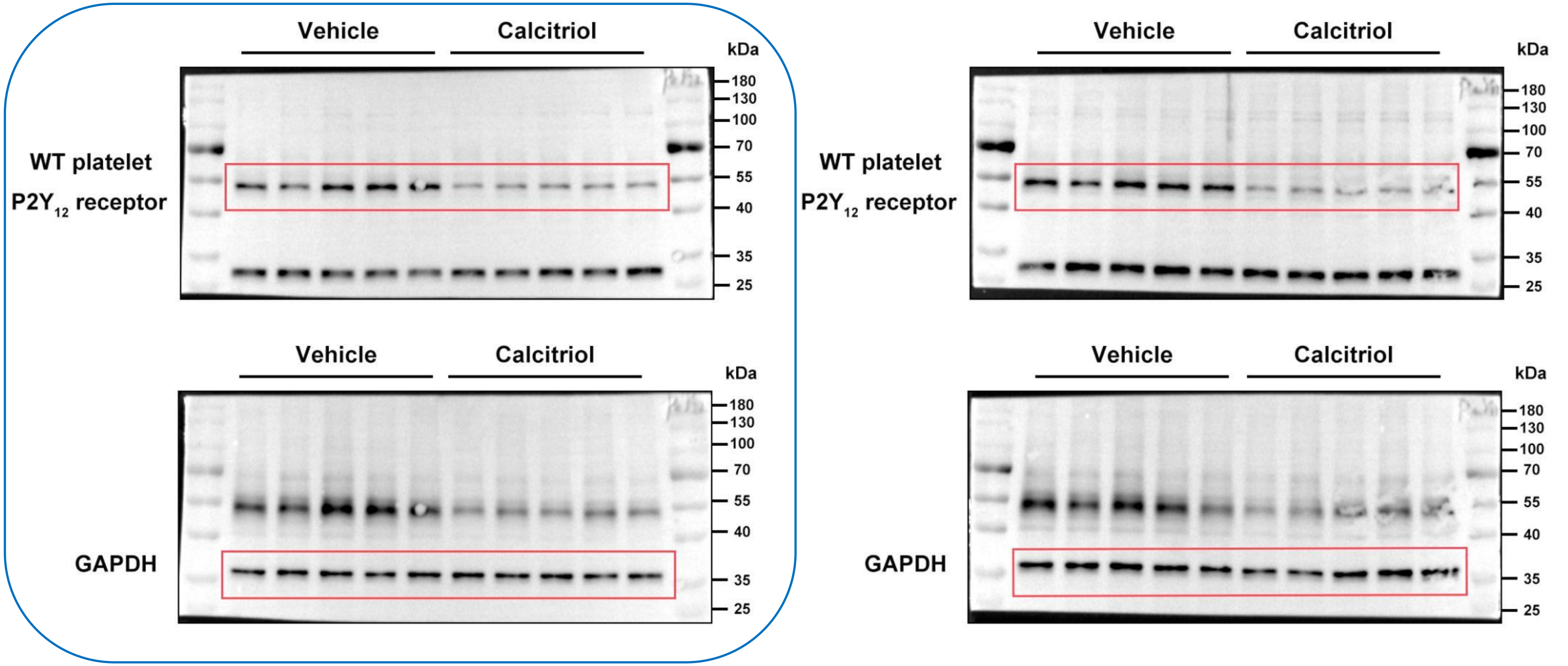

Representative figures

Figure 4F

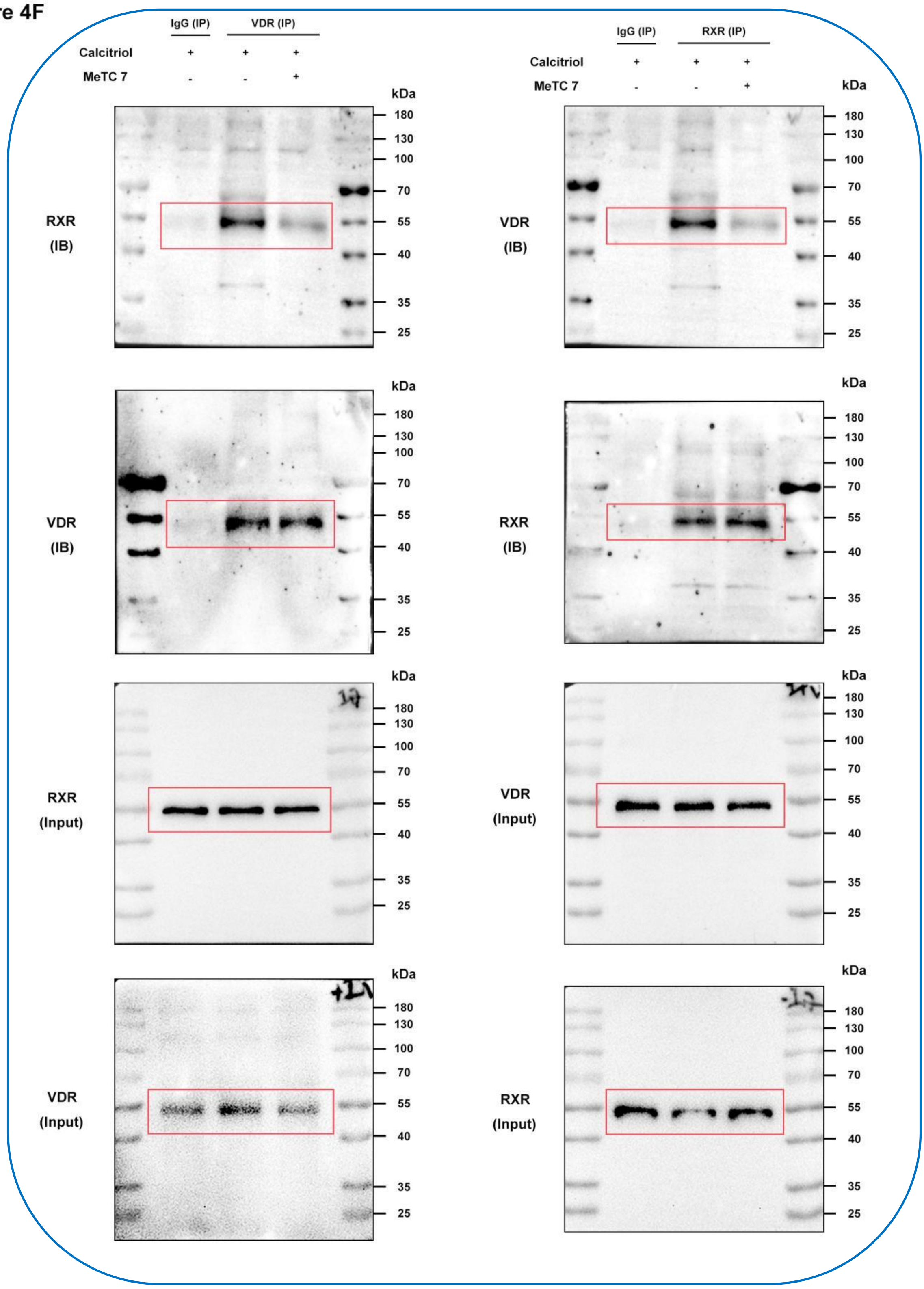

Representative figures

Figure 5B

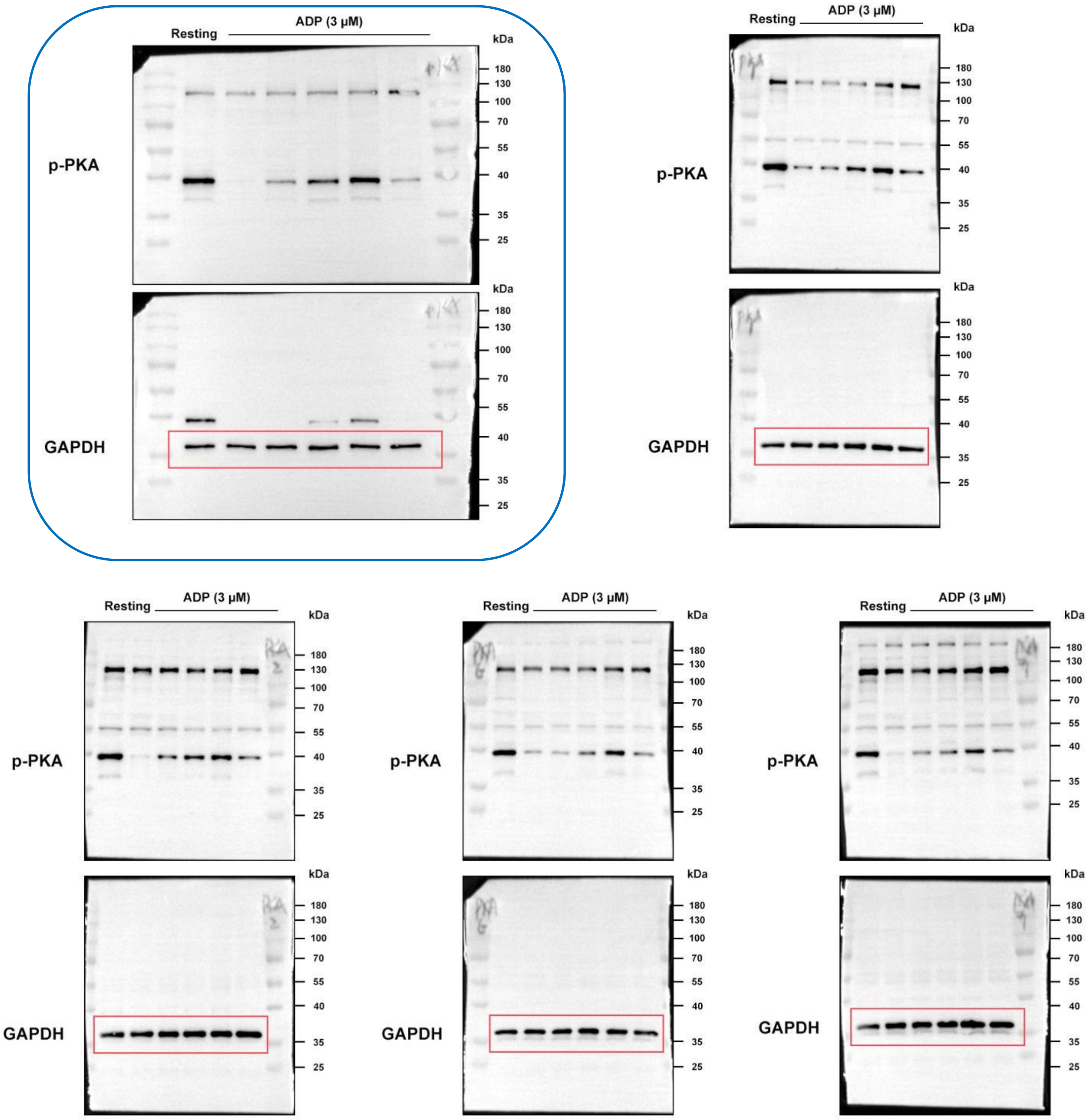

Figure 5C

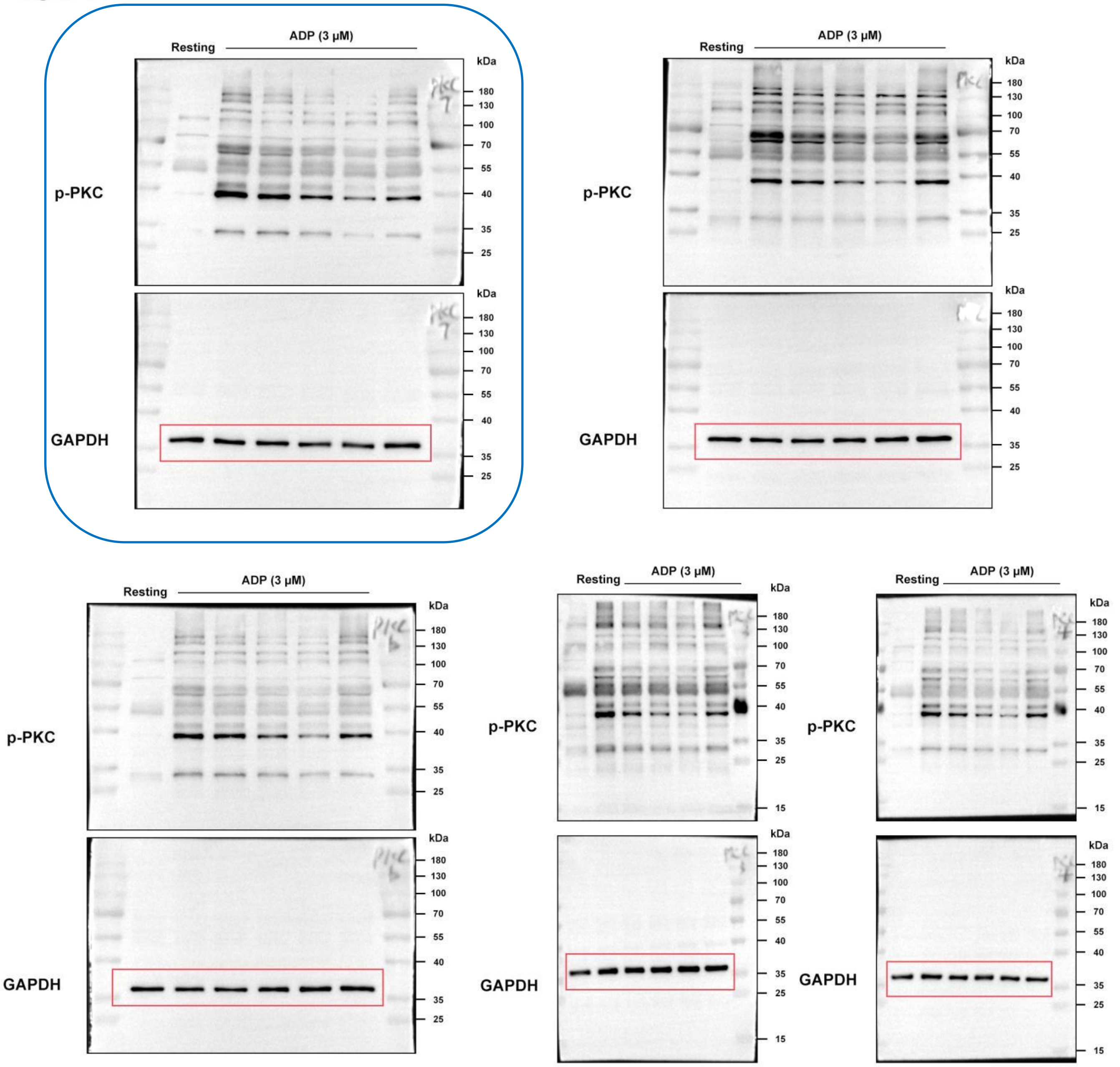

Figure 5D

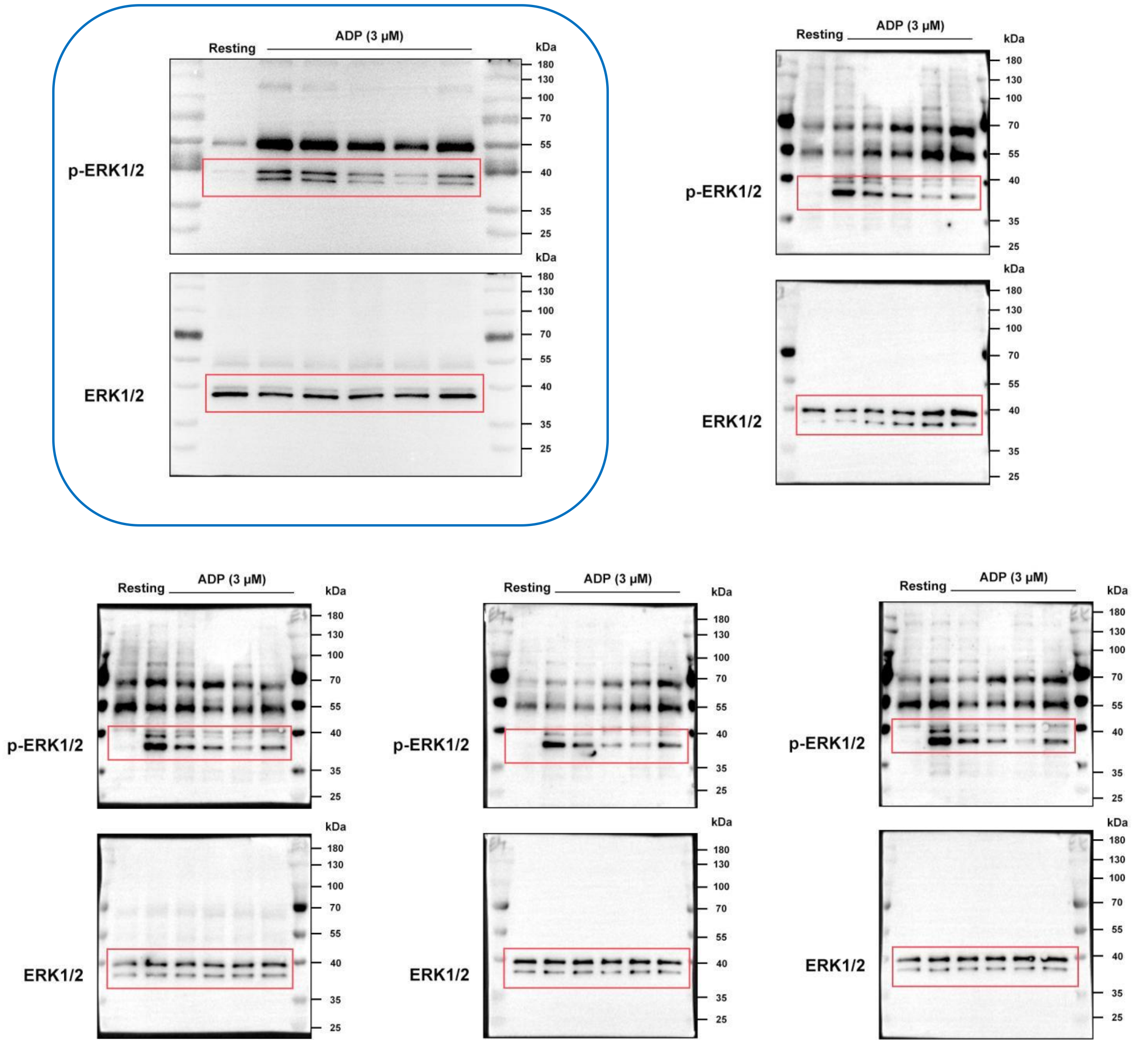
Resting
ADP (3 μM)
kDa
180
130
100
70
55
40
35
25
p-ERK1/2
ERK1/2
Resting
ADP (3 μM)
kDa
p-ERK1/2
ERK1/2
Resting
ADP (3 μM)
kDa
p-ERK1/2
ERK1/2
Resting
ADP (3 μM)
kDa
p-ERK1/2
ERK1/2
Resting
ADP (3 μM)
kDa
p-ERK1/2
ERK1/2

Figure 5D

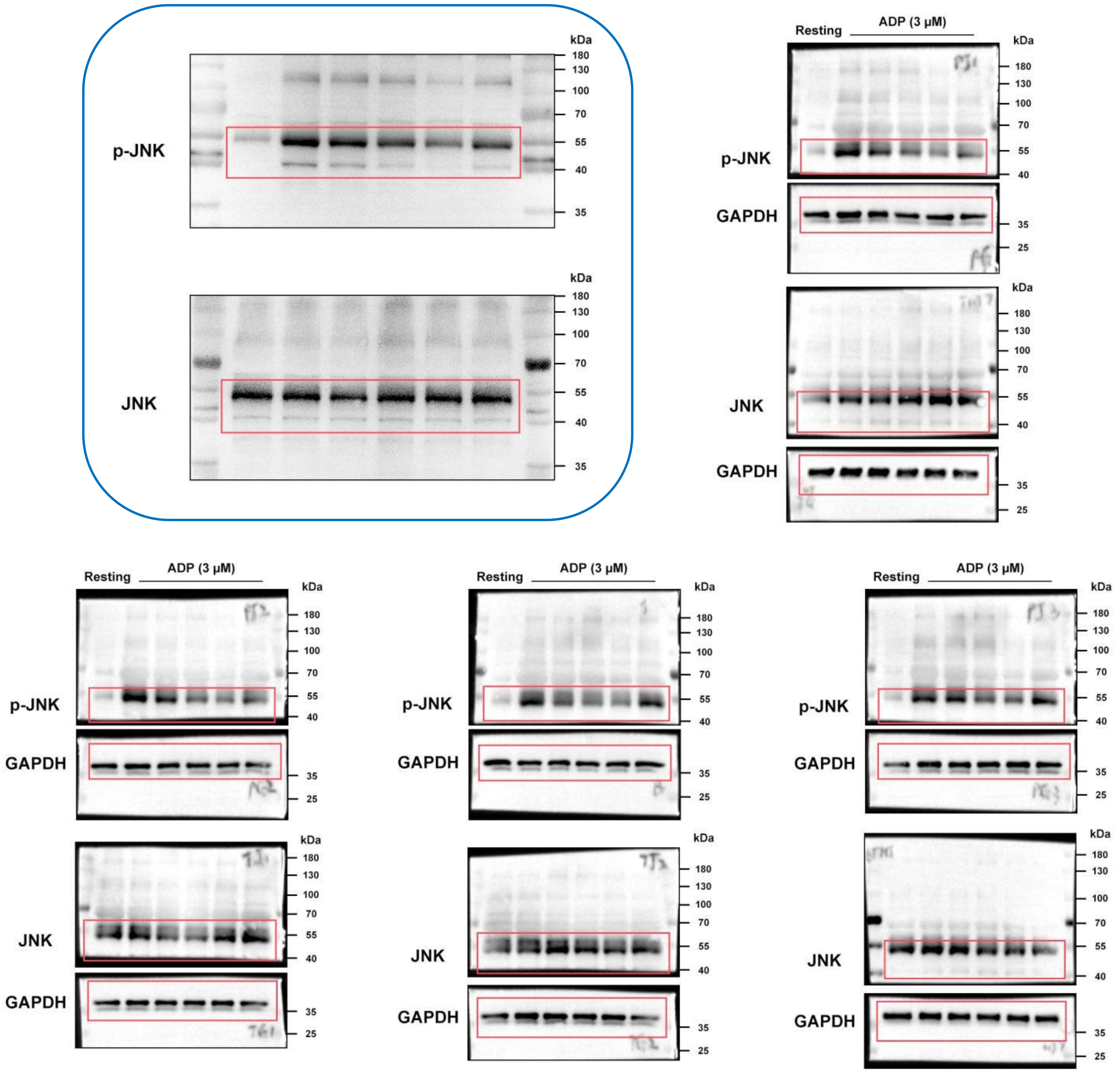

Figure 5D

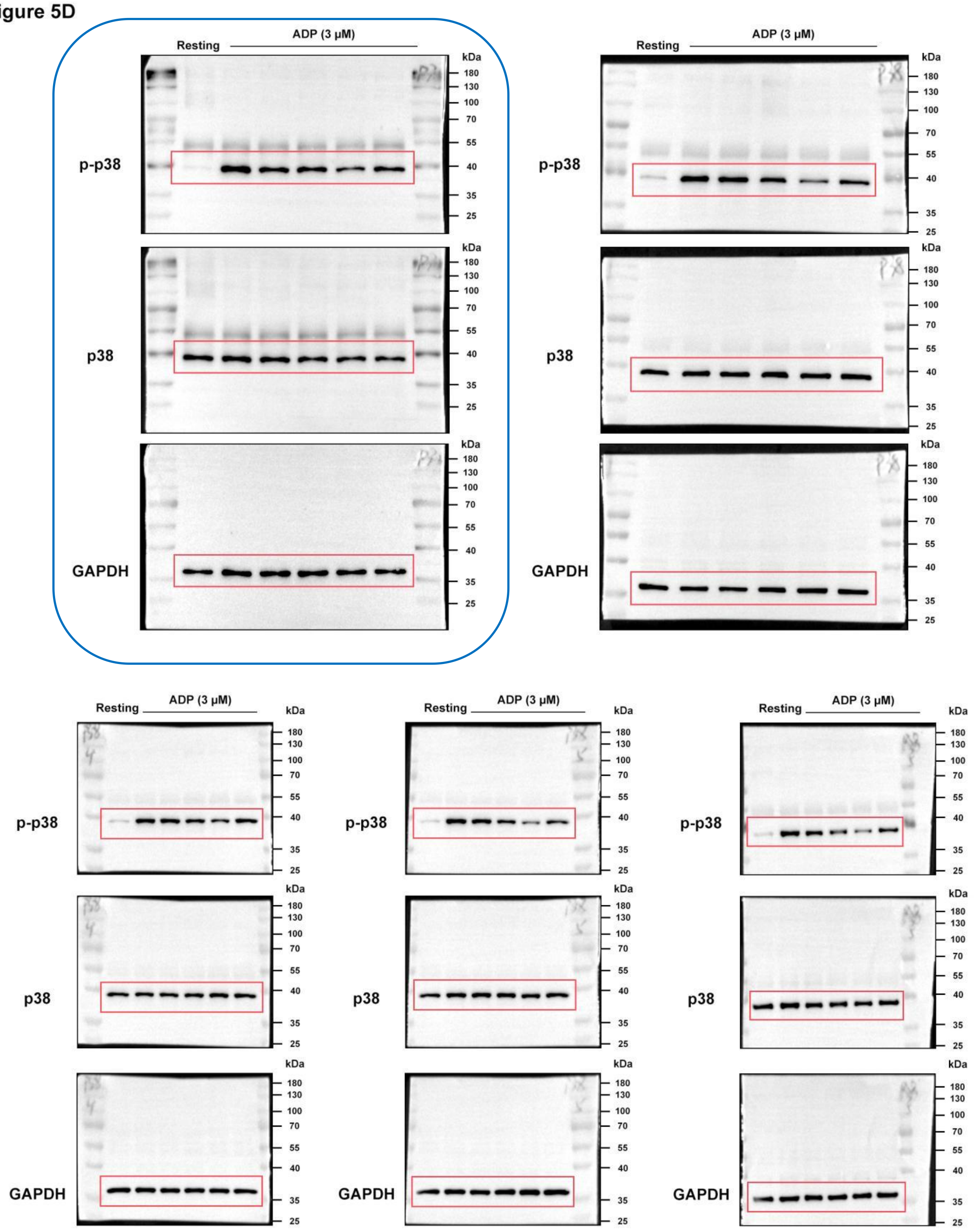

Figure 5F

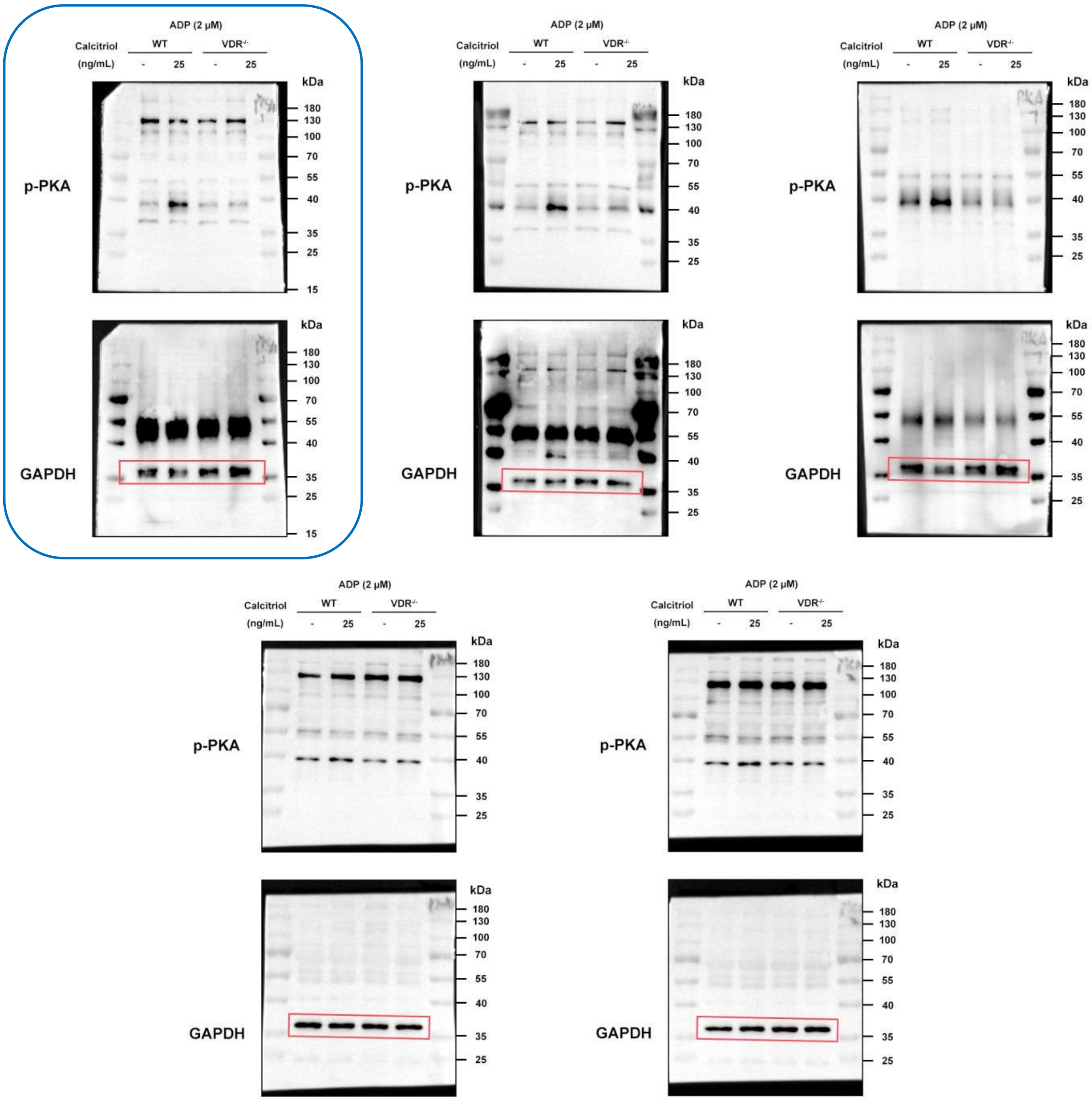
ADP (2 μM)
Calcitriol (ng/mL)
WT
VDR-/-
- 25 - 25
kDa
180
130
100
70
55
40
35
25
15
p-PKA
GAPDH
ADP (2 μM)
Calcitriol (ng/mL)
WT
VDR-/-
- 25 - 25
kDa
p-PKA
GAPDH
ADP (2 μM)
Calcitriol (ng/mL)
WT
VDR-/-
- 25 - 25
kDa
p-PKA
GAPDH
ADP (2 μM)
Calcitriol (ng/mL)
WT
VDR-/-
- 25 - 25
kDa
p-PKA
GAPDH
ADP (2 μM)
Calcitriol (ng/mL)
WT
VDR-/-
- 25 - 25
kDa
p-PKA
GAPDH

Figure 5G

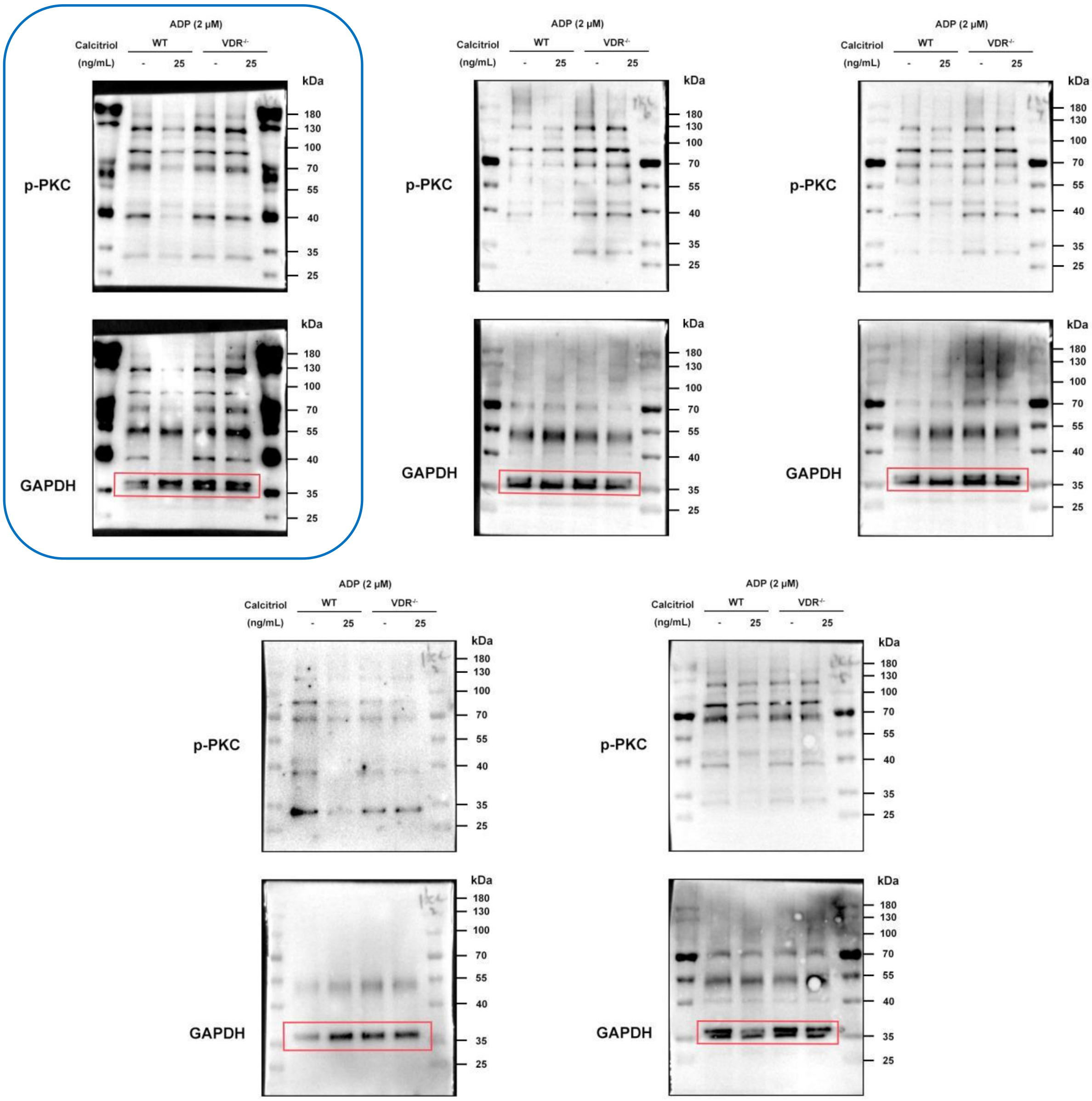
ADP (2 μM)
Calcitriol (ng/mL)
WT
VDR-/-
- 25 - 25
kDa
180
130
100
70
55
40
35
25
p-PKC
GAPDH

Figure 5H

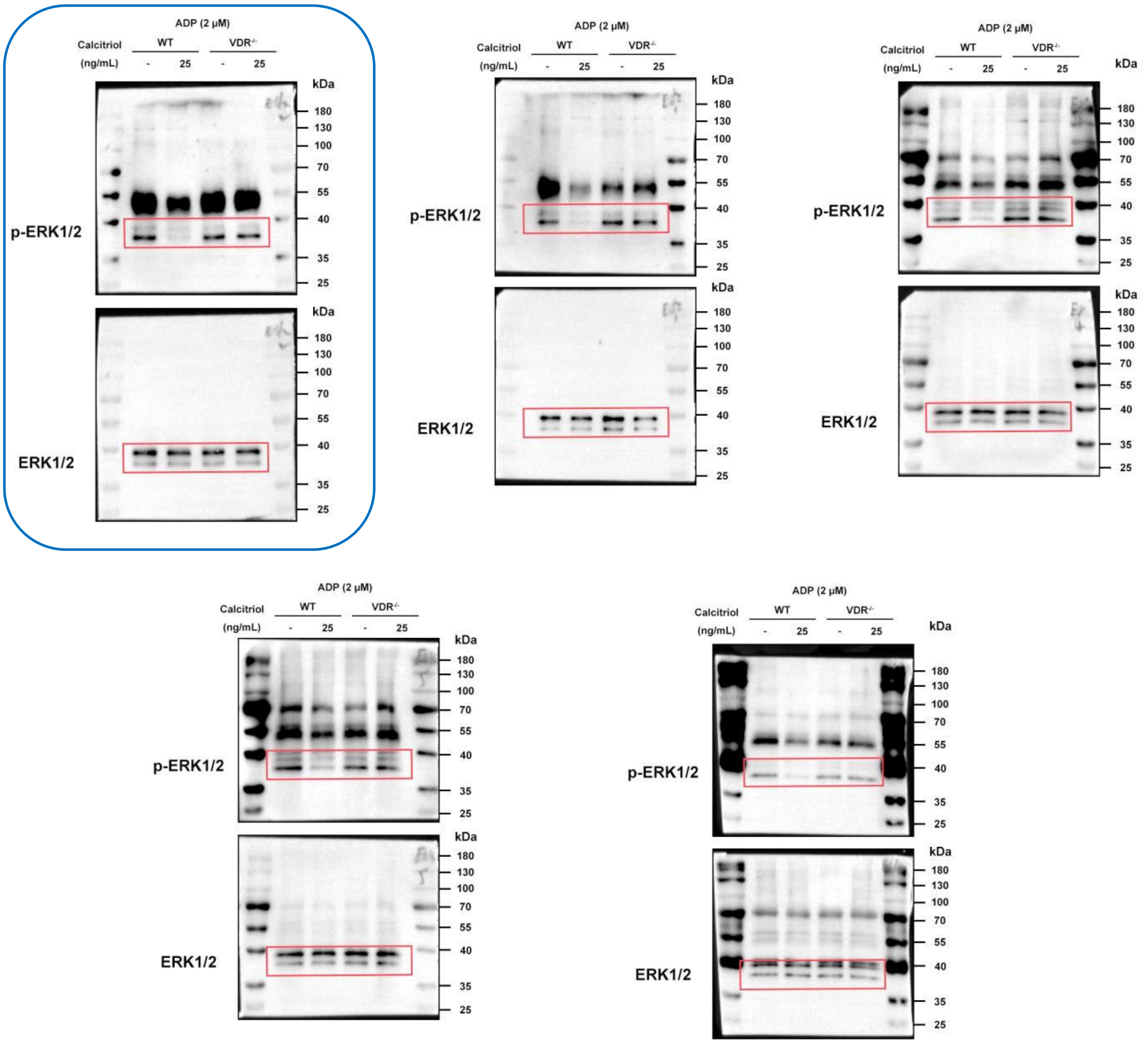
ADP (2 μM)
Calcitriol
(ng/mL)
WT
VDR-/-
-
25
kDa
180
130
100
70
55
40
35
25
p-ERK1/2
ERK1/2

Figure 5H

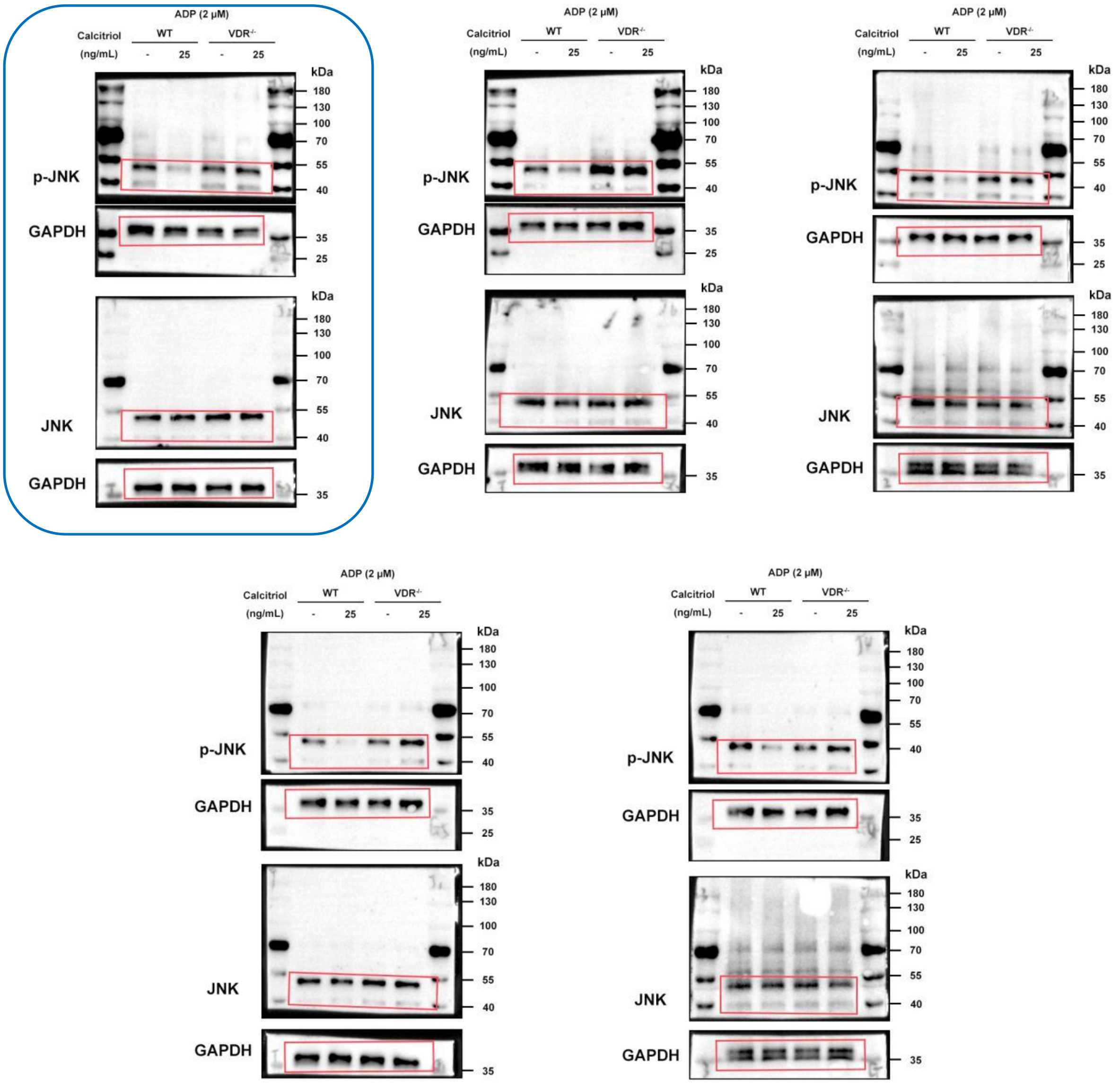
ADP (2 μM)
Calcitriol
(ng/mL)
WT
VDR-/-
-
25
-
25
kDa
180
130
100
70
55
40
35
25
p-JNK
GAPDH
JNK
GAPDH

Figure 5H

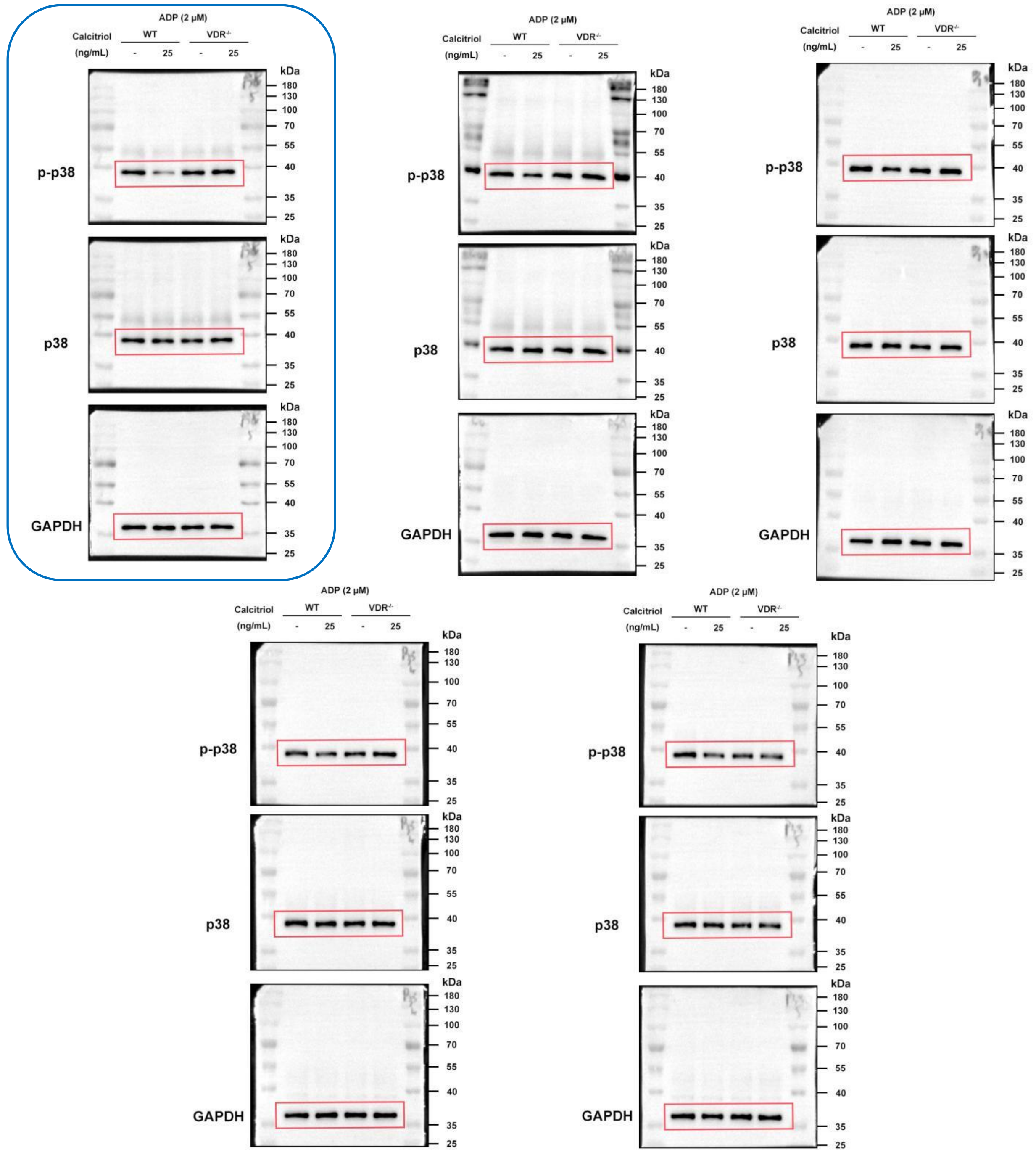

ADP (2 μM)
Calcitriol (ng/mL)
WT
VDR-/-
- 25 - 25
kDa
180
130
100
70
55
40
35
25
p-p38
p38
GAPDH

Figure 7H

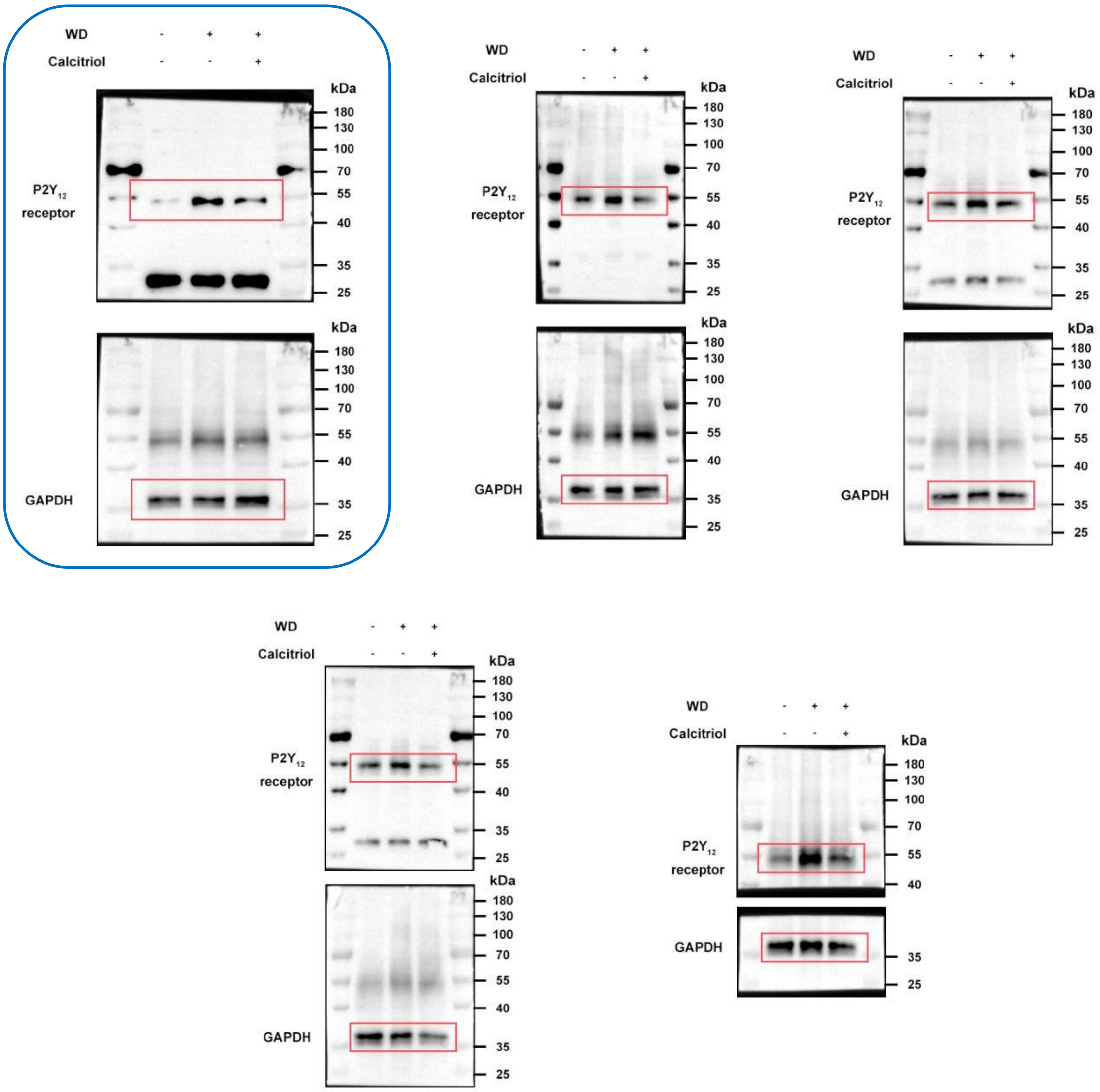
WD
Calcitriol
kDa
180
130
100
70
55
40
35
25
P2Y12 receptor
GAPDH

Supplementary Figure 7B

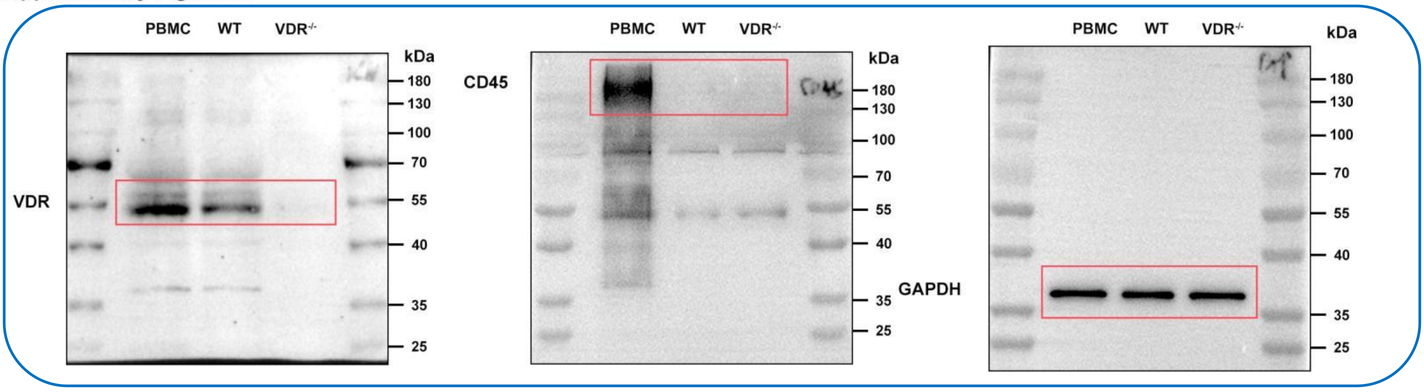

Supplementary Figure 10B

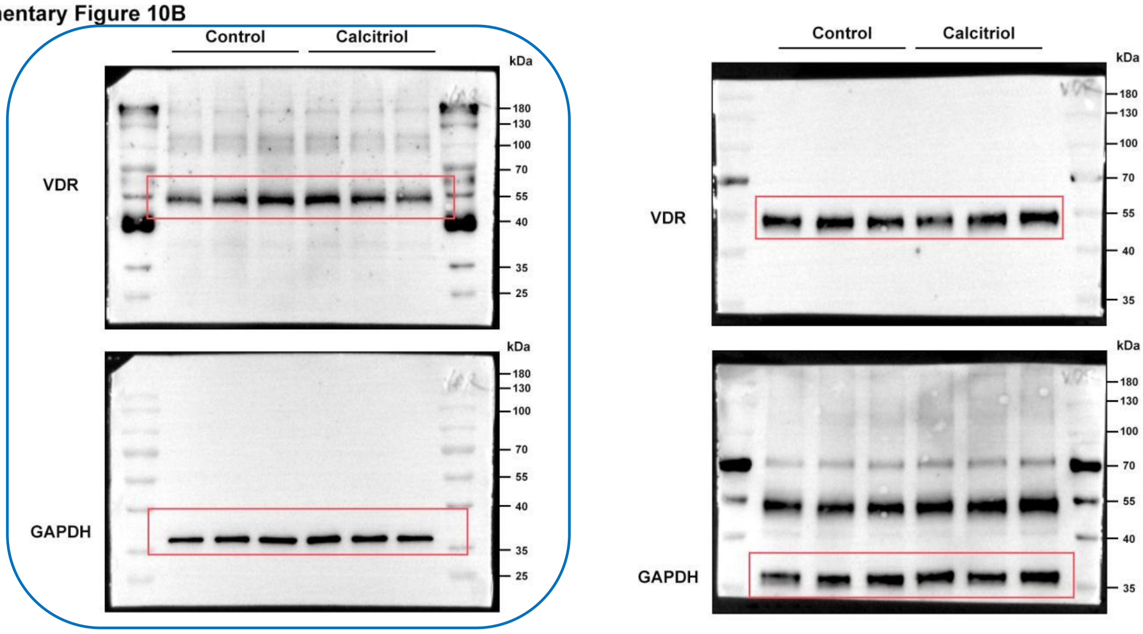

Supplementary Figure 12B

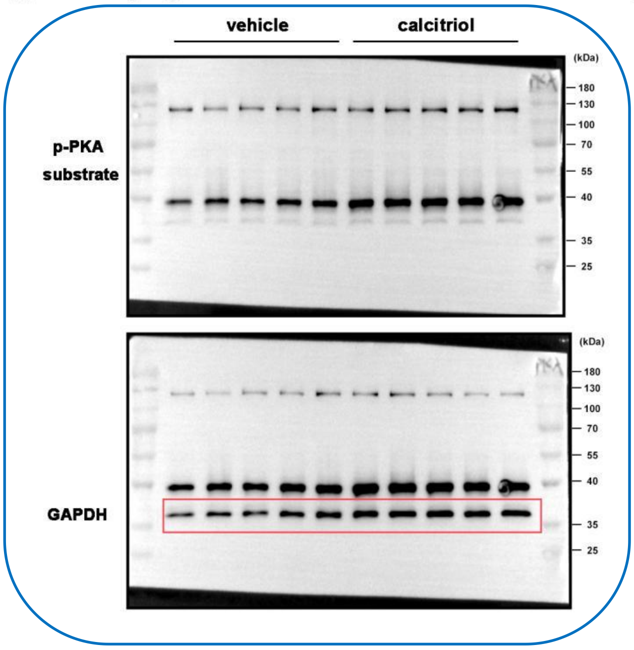

Supplementary Figure 12C

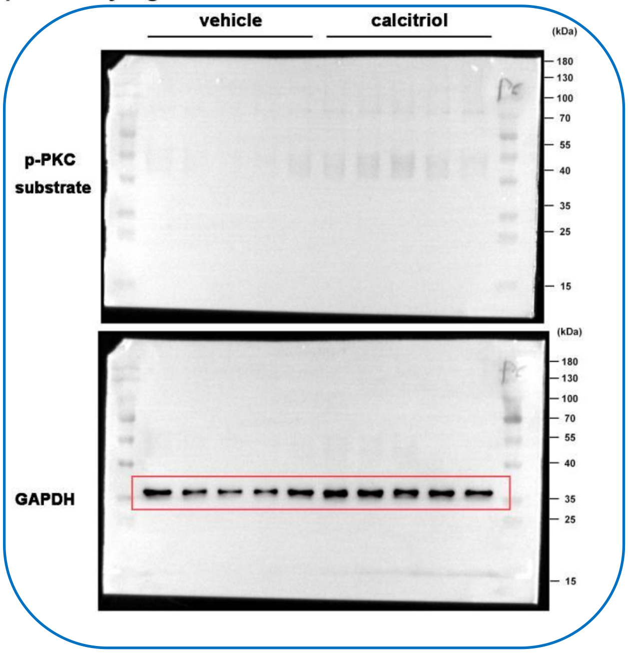

Supplement: Supplemental Methods and Supplemental Tables 1 to 7 and Supplemental Figures 1 to 24 [file mmc1.pdf]
