# Supplementary material for: Calcitriol Suppresses Platelet Activation and Thrombosis, Mitigating Cardiovascular Risks in Metabolic Dysfunction–Associated Steatotic Liver Disease
Source: JACC Basic Transl Sci. 2026 Jul 10;11(8):101632. doi: 10.1016/j.jacbts.2026.101632 (PMC13380722; doi:10.1016/j.jacbts.2026.101632)
Supplement: Supplemental Material [file mmc2.docx]

**Contents**

1. **Supplemental Methods** .................................................................................................................................4
2. **Supplementary Tables**..................................................................................................................................26

**Supplementary Table 1.** Characteristics of healthy participants enrolled in the study.....................................26

**Supplementary Table 2.** Characteristics of the healthy participants for calcitriol intervention........................27

**Supplementary Table 3.** Baseline characteristics of healthy participants and MASLD patients......................28

**Supplementary Table 4.** Characteristics of MASLD patients enrolled in the study (n=89) ............................29

**Supplementary Table 5.** Characteristics of the MASLD patients for calcitriol intervention.........................30

**Supplementary Table 6.** Antibodies used..........................................................................................................31

**Supplementary Table 7.** Primers used................................................................................................................32

1. **Supplementary Figures**.................................................................................................................................33

**Supplementary Figure 1.** Ingestion of calcitriol inhibits human platelet P-selectin expression and integrin αIIbβ3 activation...................................................................................................................................................33

**Supplementary Figure 2.** Correlation between the elevation of plasma calcitriol levels and the reduction of platelet reactivity in healthy individuals..............................................................................................................34

**Supplementary Figure 3.** Ingestion of calcitriol inhibits mouse platelet activation..........................................35

**Supplementary Figure 4.** Calcitriol treatment does not significantly affect bleeding time...............................37

**Supplementary Figure 5.** Calcitriol significantly reduced P-selectin release and platelet integrin αIIbβ3 activation in healthy human platelets stimulated with ADP or collagen..............................................................38

**Supplementary Figure 6**. Calcitriol dose titration for human platelet function..................................................39

**Supplementary Figure 7.** Platelet ultrastructure and count remained unchanged in VDR-deficient mice.........40

**Supplementary Figure 8.** Calcitriol dose titration for mouse platelet function..................................................41

**Supplementary Figure 9.** The resting status of P-selectin exposure and JON/A binding in Figure 3D and 3E.42

**Supplementary Figure 10.** VDR expression in Meg-01 cells remained unchanged following calcitriol treatment................................................................................................................................................................43

**Supplementary Figure 11.** Calcitriol ingestion increased VDR enrichment within the P2Y_12_ promoter region in mouse megakaryocytes.....................................................................................................................................44

**Supplementary Figure 12.** Calcitriol directly modulates the cAMP-PKA pathway in resting platelets............45

Supplementary Figure 13. The severity of vitamin D deficiency was associated with clinically significant platelet hyperactivation in MASLD patients.........................................................................................................46

Supplementary Figure 14. Correlation between the elevation of plasma calcitriol levels and the reduction of platelet reactivity in MASLD individuals.............................................................................................................47

Supplementary Figure 15. Comparative analysis of platelet sensitivity to calcitriol in healthy and MASLD individuals.............................................................................................................................................................48

**Supplementary Figure 16.** MASLD induced by a MCDD in WT mice.............................................................49

**Supplementary Figure 17.** Calcitriol potentiates the antiplatelet effects of P2Y_12_ inhibitors in MASLD mice.......................................................................................................................................................................50

**Supplementary Figure 18.** MCDD-induced MASLD is associated with a significant decrease in vitamin D levels......................................................................................................................................................................51

**Supplementary Figure 19.** Calcitriol inhibits platelet function and thrombosis in mice with MCDD-induced MASLD.................................................................................................................................................................52

**Supplementary Figure 20.** Calcitriol did not significantly affect fibrinolytic activity in MASLD mice injected with collagen and epinephrine...............................................................................................................................53

**Supplementary Figure 21.** Determination of ventricular volume by the modified Simpson method................54

**Supplementary Figure 22.** Calcitriol improved cardiac function in MASLD mice following myocardial I/R injury, evidenced by M-mode echocardiography..................................................................................................55

**Supplementary Figure 23.** Microvascular thrombosis is closely correlated with myocardial infarct area and LVEF in MASLD mice.........................................................................................................................................56

**Supplementary Figure 24.** Calcitriol protects MASLD mice from severe brain infarction after MCAO..........57

**Supplementary Methods**

1. **Human studies**

The study and all experimental procedures were performed in accordance with the Declaration of Helsinki and approved by the Institutional Review Board Fudan University Zhongshan Hospital. Written informed consent was obtained from participants before inclusion in our human studies.

1. **An observational discovery study in healthy participants**

This study aimed to investigate the potential association between plasma calcitriol levels and platelet reactivity in healthy participants, with a target correlation coefficient of 0.30, 90% power, and two-sided alpha=0.05. Sample size calculations indicated that a minimum of 113 participants were needed^1^. In January to April 2025, we enrolled 143 healthy participants at Fudan University Zhongshan Hospital. We excluded participants with cardiovascular disease, metabolic dysfunction-associated steatotic liver disease (MASLD), renal dysfunction, diabetes mellitus, hypertension, hyperlipidemia, malignancy, pregnancy, infection, hepatic surgery, antiplatelet medication use, or psychiatric disorders. After applying these exclusion criteria, 25 participants were excluded, leaving 118 participants for analysis. Baseline characteristics are detailed in *Supplementary Table 1*.

1. **Calcitriol intervention in healthy participants**

This study aimed to investigate the effects of calcitriol on platelet activation in healthy volunteers. Ten participants aged 18 or older were recruited from Fudan University through advertisements and chart reviews. All study materials were approved by the Fudan University Zhongshan Hospital Institutional Review Board, and informed consents were obtained from each participant after explaining the study design and potential risks. Exclusion criteria included recent anti-platelet drug usage, hypercalcaemia, hyperparathyroidism, osteomalacia, kidney stones, sarcoidosis, diabetes mellitus, significant chronic illnesses, and pregnancy. All participants were in good general health without self-reported cardiovascular disease (*Supplementary Table 2*). Participants were 27.0 ± 2.6 years of age, 40% males, nonsmokers, without hypertension and diabetes. To evaluate the impact of calcitriol on platelet function, a within-subject design was employed, comparing baseline measurements to those obtained after two weeks of standardized calcitriol administration (two calcitriol capsules per day, total of 0.5 µg/d). The dosage of calcitriol supplementation was based on a previous clinical study^2^. Participants were advised to maintain their usual dietary and lifestyle habits during the trial. Blood samples were drawn from the antecubital vein at baseline and after two weeks of calcitriol treatment. Platelet-rich plasma (PRP) was harvested and subjected to aggregation assays, while washed platelets (WPs) were isolated and quantified by flow cytometry. Based on previous studies^3^, ten participants per group was sufficient to detect at least 15% change in platelet aggregation, P-selectin expression, and integrin with 90% power and two-sided alpha=0.05, assuming a standard deviation of approximately 10%.

1. **Observational discovery studies in patients with MASLD**

To investigate the potential association between plasma calcitriol levels and platelet reactivity in patients with MASLD, we aimed to achieve a significant correlation coefficient of 0.4 with 90% power using a two-tailed test. Based on sample size calculations, a minimum of 61 participants was required^1^. Between January 2025 and April 2025, we consecutively enrolled 276 MASLD patients from Fudan University Zhongshan Hospital. MASLD was diagnosed based on the following criteria: (1) hepatic steatosis detected by abdominal ultrasound in more than 5% of hepatocytes. (2) presence of ≥1 of 5 cardiometabolic criteria: ① Body mass index ≥23 kg/m^2^ or waist circumference ≥94 cm (for males) or ≥80 cm (for females); ② Fasting glucose ≥ 5.6 mmol/L or 2-hour post-load glucose level ≥ 7.8 mmol/L or hemoglobin A1c ≥5.7%; ③ Blood pressure ≥130/85 mmHg; ④ Plasma triglyceride ≥1.7 mmol/L; ⑤ Plasma high-density lipoprotein cholesterol < 1.0 mmol/L (for males) and < 1.3 mmol/L (for females). (3) exclusion of other liver diseases^4^. All individuals were first-visit patients without prior MASLD-related or anti-platelet treatment. We excluded patients with cardiovascular disease, renal dysfunction, malignant tumors, pregnancy, infectious diseases, hepatic surgery, or psychiatric disorders. Based on these criteria, 187 patients were excluded, leaving 89 MASLD patients without anti-platelet drug use before blood sample collection. The baseline characteristics of included individuals are presented in *Supplementary Table 4*. Plasma calcitriol concentration and platelet aggregation in PRP were measured, and a correlation analysis was conducted to assess their relationship. MASLD patients were subsequently stratified into tertiles based on baseline plasma calcitriol concentrations. ADP- and collagen-induced platelet aggregation in PRP were then compared statistically across these tertiles.

1. **Calcitriol intervention in MASLD patients**

The objective of this study was to investigate the effects of calcitriol on platelet activation in patients with MASLD. The study protocol and all associated materials were approved by the Fudan University Zhongshan Hospital Institutional Review Board. Written informed consent was obtained from each participant after a thorough explanation of the study design and any potential risks. Based on previous studies^3^, a power analysis indicated that 10 participants would be sufficient to detect a 15% or greater change in platelet aggregation, P-selectin expression, and integrin αIIbβ3 activation rates with 90% power and a 5% alpha level. This calculation assumed a standard deviation of approximately 10%. During February 2025, 10 participants aged 18 years or older were recruited from Fudan University Zhongshan Hospital. Baseline characteristics of the included participants are presented in *Supplementary Table 5*. All participants were first-visit MASLD patients and had no prior history of MASLD-related or antiplatelet treatment. MASLD was diagnosed based on the criteria mentioned above^4^. Patients were excluded if they presented with any of the following conditions: cardiovascular disease, hypercalcemia, hyperparathyroidism, osteomalacia, sarcoidosis, kidney stones, renal dysfunction, malignancy, pregnancy, active infection, a history of hepatic surgery, or psychiatric disorders. Using a within-subject design, baseline measurements were compared to those obtained after two weeks of standardized calcitriol administration (0.5 µg/day, administered as two capsules). Blood samples were collected from the antecubital vein at baseline and at two weeks post-calcitriol treatment. Platelet-rich plasma was prepared and used in aggregation assays. Washed platelets were isolated and quantified via flow cytometry.

1. **Animal studies**

All animal procedures adhered to the National Institutes of Health Guidelines for Laboratory Animal Care (NIH Publication No. 85-23, revised 1996) and were approved by the Zhongshan Hospital, Fudan University Animal Care and Use Committee. Wild-type (WT) C57BL/6J male mice were obtained from Shanghai JieSiJie Laboratory Animals, while VDR-deficient (*Vdr^-/-^*) mice were acquired from SaiYe Laboratory. Genotypes were confirmed using polymerase chain reaction (PCR) and western blotting (WB). Mice had ad libitum access to water and standard chow and were housed in specific pathogen-free facilities with a 12-hour light-dark cycle. The animals were randomly assigned to different groups for the experiments on pulmonary thromboembolism, myocardial ischemia/reperfusion (I/R), middle cerebral artery occlusion (MCAO), and ferric trichloride (FeCl_3_)-induced thrombosis using a random number generator in Microsoft Excel software. Pentobarbital sodium was administered intraperitoneally for both euthanasia (200 mg/kg) and anesthesia (50 mg/kg). Euthanasia was conducted in a CO_2_ chamber followed by cervical dislocation to ensure death. Death was confirmed by the absence of a heartbeat and reflexes.

1. **Reagents and materials**

Adenosine diphosphate (ADP), collagen, thrombin, epinephrine, and luciferin were purchased from Chrono-Log (Havertown, PA). Human fibrinogen, FITC-labeled phalloidin, apyrase, prostaglandin E1 (PGE1), calcein acetoxymethyl ester, 25-OH-D_3_, and Evans blue were purchased from Sigma-Aldrich (St Louis, MO). Protein A/G Plus agarose was purchased from Santa Cruz Biotechnology (Dallas, USA). Calcitriol (*in vivo* human study) was produced by Catalent Germany Eberbach GmbH (Eberbach, Germany). Calcitriol (*in vitro experiments*), MeTC7 (VDR inhibitor), and clopidogrel were purchased from MCE (New Jersey, USA). Methionine- and choline-deficient diet (MCDD) and Western diet (WD) were purchased from Dysts (Wuxi, China). Triphenyltetrazolium chloride was purchased from Solarbio (Beijing, China). Enzymatic chromatin IP Kit was purchased from Cell Signaling Technology (Danvers, USA). CD61 microbeads and MidMACS starting Kit were purchased from Miltenyi Biotechnology (Bergisch Gladbach, Germany). Dual-luciferase report assay system was purchased from Promega (Madison, USA). Vitamin D, D-dimer, and cAMP ELISA kits were obtained from MLBIO (Shanghai, China). The antibodies used in the study were listed in the *Supplementary Table 6*.

1. **Platelet preparation**

Human platelets were isolated following established protocols^5,6^. Blood was collected from the antecubital vein and mixed with acidcitrate dextrose (85 mM sodium citrate, 71.38 mM citric acid, and 27.78 mM glucose) buffer (9:1 vol/vol). PRP was obtained by centrifuging whole blood at 300 g for 10 minutes. Resuspended platelets (RP) were prepared by centrifuging PRP at 720 g for 10 minutes and resuspending the platelet pellet in Tyrode’s solution (137 mM NaCl, 12 mM NaHCO_3_, 2 mM KCl, 0.34 mM Na_2_HPO_4_, 1 mM MgCl_2_, 5.5 mM glucose, and 5 mM HEPES; pH 7.4). This preparation was used for experiments where a rapid isolation was sufficient. Additionally, WP were prepared by filtering PRP through a Sepharose 2B column (Sigma-Aldrich) equilibrated with Tyrode’s solution (134 mM NaCl, 2.9 mM KCl, 340 μM Na_2_HPO_4_·12H_2_O, 12 mM NaHCO_3_, 20 mM HEPES, 1 mM MgCl_2_, 5 mM glucose, pH 7.35, 37℃). WP were chose for aggregation assays and flow cytometry assays to ensure that the platelet responses were evaluated in a highly controlled environment, completely free from the potential confounding effects of residual plasma factors.

  Mouse platelets were prepared as previously described^5,6^. Mice were anesthetized with an intraperitoneal injection of pentobarbital sodium (50 mg/kg body weight). Blood was subsequently collected from the abdominal aorta using a syringe containing 3.8% citrate as an anticoagulant (9:1 vol/vol), along with 1 U/mL apyrase and 0.1 μg/mL PGE1 to prevent platelet activation. Two milliliters of whole blood was then centrifuged at 300 g for 2 minutes to separate PRP. The PRP was further centrifuged at 420 g for 10 minutes to isolate platelets, which were finally resuspended in Tyrode buffer.

1. **Mouse hematology**

Mouse blood was collected from the abdominal aorta using citrate as an anticoagulant. Blood cell counts were then determined using an automatic hematology analyzer (Mindray, Shenzhen, China).

1. **Mouse peripheral blood mononuclear cells (PBMCs) preparation**

Whole blood was collected from WT mice via the abdominal aorta using a citrate-containing syringe (9:1 vol/vol) and processed within two hours. The blood was diluted with Hanks’ balanced salt solution (HBSS, Sigma-Aldrich, H6648) and PBMCs were isolated using Ficoll-Paque PREMIUM 1.804 (GE Healthcare Biosciences, 28-4039-56) following the manufacturer’s protocol. The cells were immediately used for RNA extraction and protein sample preparation.

1. **Mouse bone marrow isolation and CD61^+^ megakaryocyte preparation**

Mouse bone marrow aspirate samples were collected in tubes containing EDTA. CD61^+^ megakaryocytes were isolated using CD61 MicroBeads (Miltenyi Biotech, Germany) according to the manufacturer’s instructions as previously reported^7^. By using the magnet, isolated megakaryocytes were poured and used immediately for RNA extraction.

1. **Cell culture**

Human megakaryocytic cells (Meg-01) were cultured in RPMI-1640 medium (Invitrogen) supplemented with 10% heat-inactivated fetal bovine serum and 20 mM L-glutamine for at least five passages to adapt to the culture conditions. Meg-01 cells were then treated with 25 pg/mL calcitriol for 16 hours. To pharmacologically inhibit the vitamin D receptor (VDR), 2 nM MeTC7 was added to some cultures. After treatment, cells were collected and processed for RNA and protein extraction.

1. **Platelet aggregation and secretion**

Platelet aggregation experiment was performed following a previously established protocol^5,6^. The concentration of WPs was set at 300 × 10^9^ platelets/L. For human platelet aggregation and ATP secretion assays, platelets were preincubated with varying concentrations of calcitriol or vehicle control at 37℃ for 1 minute under stirring (1200 rpm), followed by a 10-minute incubation at rest. For mouse platelet aggregation and ATP secretion assays, platelets were preincubated with MeTC7 or vehicle control at 37℃ for 1 minute with stirring (1200 rpm), followed by 5 minutes at rest. Calcitriol or vehicle control was then added, mixed by stirring (1200 rpm) for 1 minute, and incubated at rest for 10 minutes. Platelet aggregation in response to collagen, thrombin, and ADP with/without treatment was measured using a lumiaggregometer (Model 400 VS; Chrono-Log) under standardized conditions: stirring at 1200 rpm and temperature at 37℃. Fibrinogen (0.25 g/L) was added prior to ADP stimulation to facilitate maximal platelet aggregation. Platelet dense granule secretion was assessed concurrently with collagen and thrombin stimulation by measuring the release of adenosine triphosphate (ATP) using CHRONO-LUME reagent (Chrono-Log)^5,6^. The aggregation amplitude of aggregation tracings was quantified as an aggregation ratio, reflecting the extent of platelet aggregation. Similarly, the ATP release amplitude of release tracings was calculated as a release ratio, representing the extent of platelet ATP release.

1. **Human PRP aggregation**

Human PRP was isolated as previously described^8^. Whole blood was drawn from volunteers and anticoagulated with sodium citrate (9:1 vol/vol). PRP was prepared by centrifugation at 100 × g for 10 minutes at 22 ℃. Platelet-poor plasma (PPP) was obtained by further centrifuging the remaining blood samples at 11,000 × g for 2 minutes. Platelets were then counted using a hemocytometer and adjusted to a concentration of 150 × 10^9^ platelets/L with PPP. Platelet aggregation was induced by adding ADP or collagen at the indicated concentrations and measured using a lumiaggregometer under stirring conditions (1200 rpm) at 37℃.

1. **Mouse PRP aggregation**

Mouse PRP aggregation was measured using a previously described protocol^9,10^. Blood was collected from the abdominal aorta of anesthetized mice (50 mg/kg pentobarbital sodium) using a syringe containing 3.8% citrate as an anticoagulant (9:1 volume ratio of blood to citrate). Red blood cells were removed from the blood by centrifugation at 100 g for 7 minutes. Blood from two mice of the same strain was typically pooled and diluted with PPP from the same strain to achieve a final platelet count of 300 × 10^9^ /L. Samples were incubated at 37℃ for 20 minutes before platelet aggregation experiments. Aggregation was induced with collagen or ADP in 300-μL aliquots of PRP and measured using a ChronoLog Corp. Lumiaggregometer.

1. **Platelet spreading**

In preparation for human platelet spreading, RPs (20 × 10^9^/L) were incubated with calcitriol or vehicle control. The mixture was homogenized by gently pipetting up and down 10 times using a 2.0 mL Pasteur pipette. Then, the platelets were incubated at rest at 37℃ for 10 minutes. Prior to mouse platelet spreading, RPs (20 × 10^9^/L) were subjected to a sequential incubation protocol. Initially, platelets were incubated with MeTC7 or vehicle control and mixed by 10 gentle pipetting actions using a 2.0 mL Pasteur pipette, followed by a 5-minute resting incubation at 37℃. Subsequently, calcitriol or vehicle control was added, mixed in the same manner, and incubated at rest for 10 minutes.

After treatment, RPs (20 × 10^9^/L) were pretreated and transferred onto Lab-Tek chamber slides (Nalge Nunc International, Rochester, NY) precoated with 100 μg/mL of fibrinogen, and then allowed to adhere for the indicated time at 37℃. Attached platelets were fixed and permeabilized using Cytofix/Cytoper^TM^ Fixation/Permeabilization solution (Becton Dickinson Company), and stained with FITC-labeled phalloidin after being washed with phosphate-buffered saline (PBS). A Leica SPE confocal laser scanning microscope was used to view adherent platelets. Platelet spreading area was calculated by Image-J software of random images.

1. **Clot retraction**

  Platelet clot retraction was assessed using a modified version of a previously described protocol^6^. RPs (500 × 10^9^/L) of human or mice were pretreated as described in the platelet spreading procedure. Clot retraction was initiated by adding 0.1 mL of human PPP to 0.3 mL of RPs. Thrombin (1.0 U/mL) was then added to trigger clot formation, which was monitored at 37℃ by taking photographs at specified time points using a digital camera. The clot surface area was quantified using ImageJ software, and the results are expressed as the percentage of clot retraction.

1. **Platelet P-selectin and activated integrin abundance**

Flow cytometry analysis was conducted using a modified version of a previously described protocol^6^. Fluorophore-labeled antibodies were used to detect the expression of P-selectin (P-selectin-APC for human platelets, CD62P-APC for mouse platelets) and the activated form of αIIbβ3 integrin (PAC-1-FITC for human platelets, JoN/A-PE for mouse platelets). Platelets were identified using anti-CD41 antibodies (CD41-PE in human platelets, CD41-FITC in mouse platelets). Human or mouse WPs (10 × 10^9^/L) were pretreated as described in the platelet spreading procedure and then activated for 5 minutes with thrombin, collagen, or ADP. Fluorophore-labeled antibodies were then added and incubated in the dark at room temperature for 20 minutes. Unstimulated platelet samples served as resting controls for platelet activation. At least 10,000 events were recorded for each sample. P-selectin expression, PAC-1 binding, and JoN/A binding were then analyzed by flow cytometry (FACSCalibur, Becton Dickinson Biosciences).

1. **Intravital microscopy of FeCl_3_-induced thrombus formation in mouse mesenteric arterioles**

Intravital microscopy of FeCl_3_-induced thrombus formation in mouse mesenteric arterioles was conducted using a previously described protocol^6^. Platelet depletion was induced in recipient mice by intraperitoneally injecting 20 μL of rabbit anti-mouse thrombocyte serum, followed by a 24-hour waiting period. Platelet depletion was considered successful when the platelet count decreased to less than 10% of the baseline value. Anesthesia was induced with intraperitoneal pentobarbital sodium (50 mg/kg). Mouse RPs (500 × 10^9^ /L) from different therapeutic regimens were labeled with calcein for 30 minutes and administered to recipient mice via lateral tail vein injection. Mesenteric arteriolar thrombosis was induced by applying 10% FeCl_3_ for 2 minutes. The process was observed using intravital microscopy, and the time to first thrombus greater than 20μm and occlusion time were quantified with ImageJ software.

1. **Tail bleeding assay**

Mice were anesthetized with pentobarbital (50 mg/kg, i.p.) and placed in a prone position on a warming pad. A 3 mm segment was excised from the tail tip, and the tail was immediately immersed in 10 mL of 37 ℃ saline. Bleeding time was defined as the duration from tail transection to the point at which blood flow ceased for more than 2 minutes.

1. **Microfluidic whole-blood perfusion assay**

The flow chamber assay was performed as previously described with minor modifications^5,6^. Briefly, the Bioflux plate channels were coated overnight at 4℃ with 40 μg/mL fibrillar collagen. Excess collagen was washed away with PBS, and the channels were subsequently blocked with 5% bovine serum albumin for 1 hour. Whole blood from MASLD patients and healthy participants was anticoagulated with sodium citrate, pretreated with calcitriol or vehicle, incubated with mepacrine for 30 minutes, and then perfused over fibrillar collagen-coated Bioflux plates at a shear rate of 40 dynes/cm² for 5 minutes using a Bioflux 200 system (Fluxion, South San Francisco, CA). Images were acquired, and the platelet-covered area was quantified using Bioflux software (Fluxion, San Francisco, CA, USA).

1. **Transmission electron microscopy**

Platelet ultrastructure was examined using standard scanning electron microscopy following established protocols^5^. Purified resting platelets were fixed in 50 mM cacodylate buffer (pH 7.2) containing glutaraldehyde for 1 hour at ambient temperature, washed thoroughly, and post-fixed with 1% osmium tetroxide in PBS. Dehydration was performed using a graded series of ethanol washes (30%, 50%, 75%, 85%, 95%, 100%), followed by embedding. Ultrathin sections were counterstained with lead citrate and uranyl acetate and visualized under a transmission electron microscope (FEI Tecnai G2 Spirit, Hillsboro, Oregon, USA).

1. **Reverse transcription polymerase chain reaction (RT-PCR) and qPCR**

  Total RNA was extracted from WPs using Trizol reagent (Invitrogen). Subsequently, 1 µg of RNA was reverse transcribed into cDNA using a RT-PCR kit (TaKaRa) according to the manufacturer’s instructions. CD45 is a specific marker used to deplete leukocyte contamination from platelet preparations. RT-PCR was performed using specific primers (*Supplementary Table 7*).

qPCR was performed using specific primers (*Supplementary Table 7*) and TB Green Premix Ex Taq II (TaKaRa, Dalian, China) according to the manufacturer’s protocol on an Applied Biosystems 7500 qPCR System (Thermo Fisher, Shanghai, China). Relative mRNA expression level from each sample was standardized comparing with its GAPDH.

1. **RNA Sequencing**

Total RNA was extracted from calcitriol treated mice and control mice using TRIzol reagent and quantified with a NanoDrop 2000 spectrophotometer (Thermo Fisher Scientific). RNA integrity was evaluated using an Agilent 2100 Bioanalyzer, and libraries were subsequently prepared for sequencing on an Illumina NovaSeq 6000 platform. Cutadapt was used to filter out low-quality reads, followed by a quality check using FastQC. Transcriptome sequencing and analysis were performed by LC Bio Technology CO (Hangzhou, China). All sample reads were aligned to the mouse genome using HISAT2 (version 2.2.1). Differentially expressed genes were identified using DESeq2 software, with genes having a false discovery rate (FDR) below 0.05 and an absolute fold change of 2 or greater considered differentially expressed. Gene set enrichment analysis (GSEA) was conducted to identify enriched pathways. Gene ontology (GO) gene sets from the Molecular Signatures Database (MSigDB) were employed for this analysis. The enrichment score (ES) in GSEA was calculated by ranking genes based on their significance within the two groups and assessing the distribution of proteins from each gene set across the ranked list.

1. **Chromatin immunoprecipitation (ChIP) assay**

ChIP was performed using a ChIP Assay Kit (9005S, Cell Signaling Technology, MA), following the manufacturer’s protocol with minor modifications. Meg-01 cells were cultured to 90-95% confluence in a 10 cm dish and crosslinked with 1% formaldehyde for 10 minutes at 37℃ to stabilize protein-DNA interactions. The crosslinking reaction was quenched with 0.125 M glycine. Cells were washed three times with ice-cold PBS, scraped into a conical tube, pelleted, lysed, sonicated, and immunoprecipitated using a VDR antibody (Abcam, AB109234) or an isotype IgG control (Abcam, AB171870). Immune complexes were collected using 50 µL of protein A agarose beads for 60 minutes at 4℃ with rotation. After washing once with low-salt wash buffer (0.1% SDS, 1% Triton X-100, 2 mmol/L EDTA, 20 mmol/L Tris-HCl, pH 8.0, 150 mmol/L NaCl), high-salt wash buffer (0.1% SDS, 1% Triton X-100, 2 mmol/L EDTA, 20 mmol/L Tris-HCl, pH 8.0, 1.5 mol/L NaCl), and LiCl wash buffer (250 mmol/L LiCl, 1% NP-40, 1% sodium deoxycholate, 1 mmol/L EDTA, 10 mmol/L Tris-HCl, pH 8.0), and twice with 10 mmol/L Tris-HCl, pH 8.0, and 1 mmol/L EDTA, 10 µL of supernatant was saved as input. Immune complexes were eluted with freshly prepared elution buffer (1% SDS in 0.1 M NaHCO3) and crosslinks were reversed by heating at 65℃ overnight in the presence of 0.2 M NaCl. P2Y_12_ enrichment was assessed by qPCR.

1. **ChIP Sequencing**

Raw reads were aligned to the P2Y_12_ genome using Bowtie (version 1.2.3) to identify the best alignment for each read. Genome coverage bedGraph files for the UCSC genome browser were generated using deepTools (version 3.0.2) and the bamCoverage command with the following parameters: ‘-of bedgraph -binSize 5 --normalizeUsingRPKM’.

1. **Transient transfection and luciferase assay of P2Y_12_ promoters**

Transient transfection and luciferase assays were performed as previously described^11^. Firefly luciferase constructs containing various P2Y_12_ promoter fragments were transiently transfected into HEK293T cells using Lipofectamine 3000 (Invitrogen, NY) and assayed for promoter activity. Briefly, 6 × 10^4^ cells were plated in 24-well plates 24 hours before transfection. A mixture of 2.4 µL Lipofectamine 3000 and 25 µL Opti-MEM media was prepared, followed by the addition of 0.4 µg of pGL3 plasmid containing the P2Y_12_ promoter (wild-type, mutant, or truncated constructs) and the VDR plasmid. After a 20-minute incubation to form DNA-lipid complexes, 16 ng of pRL-TK plasmid was added. The cells were transfected with the mixture for 6 hours, and the culture medium was then replaced. After an additional 48 hours of culture, cells were washed twice with cold PBS and lysed with 100 µL of lysis buffer. The protein supernatant was collected and analyzed for firefly and renilla luciferase activities using the dual-luciferase report assay system (Promega) according to the manufacturer’s instructions. Luminescence readings were obtained using a TD 20/20 luminometer (Turner Design Inc., Sunnyvale, CA), and sample values were normalized to the reference value of pGL3-Basic empty vector/empty pRL-TK. At least 5 independent transfections were performed.

1. **WB**

Pretreated WPs were stimulated with agonists for appropriate durations at 37℃ in a Chrono-Log aggregometer with stirring. Reactions were terminated by adding 2× lysis buffer (50 mmol/L Tris, 150 mmol/L NaCl, pH 7.4) containing protease and phosphatase inhibitors. Proteins were separated by SDS-polyacrylamide gel electrophoresis, transferred to polyvinylidene fluoride membranes, and immunoblotted. Immunoreactive bands were visualized by enhanced chemiluminescence and imaged with Tanon 4600SF (Tanon Science, Shanghai, China) after incubation with corresponding secondary antibodies. To ensure sample purity, CD45, a pan-leukocyte marker, was used to deplete any leukocyte contamination. Antibodies used in this study are listed in *Supplementary Table 6*.

1. **Co-immunoprecipitation** **(Co-IP)**

  Co-IP was performed as previously described^5^. Meg-01 cells were centrifuged and lysed with 300 mL of chilled 1× NP-40 lysis buffer (100 mM Tris-HCL pH 7.4, 300 mM NaCl, 2 mM NaF, 2% NP-40, 2 mM EDTA, and 2 × protease and phosphatase inhibitor solution) on ice for 30 minutes. Lysatecrntr supernatants were precleared with protein A/G-agarose beads for 3 hours at 4℃ and centrifuged. Immunoprecipitation was performed using anti-VDR or anti-RXR antibodies for 2 hours, with an isotype IgG control. The samples were then incubated with protein A/G-agarose beads overnight on a rocker at 4℃. The beads were harvested and washed three times with 1× NP-40 lysis buffer. VDR or RXR captured by the beads was detected by WB.

1. **ELISA**

PPP from mice or MASLD patients were isolated from blood by centrifugation. Vitamin D and D-dimer concentrations were measured using ELISA kits according to the manufacturer’s instructions: MLBIO ml060600 (human) and ml058557 (mouse) for vitamin D; and MLBIO ml038012 (mouse) for D-dimer. After incubation with a secondary antibody, color was developed, and the optical density was measured using a BioTek microplate reader.

1. **cAMP assay**

WPs were pre-incubated with ADP and varying concentrations of calcitriol for 5 minutes at room temperature. The reaction was terminated by adding 0.1 mol/L hydrochloric acid, followed by incubation for 20 minutes at room temperature. Subsequently, the mixture was centrifuged at 1000 g for 10 minutes. The supernatant was collected and used to measure cAMP levels using a cAMP ELISA kit (MLBIO) according to the manufacturer’s instructions.

1. **Liquid chromatography-tandem mass spectrometry (LC/MS/MS)**

A stable-isotope-dilution LC/MS/MS method was developed to specifically quantify calcitriol [1,25-(OH)_2_-D_3_] in human plasma. As described previously^12^, an internal standards solution was prepared by dissolving known amounts of 1α,25-dihydroxy-vitamin-D_3_ [d6-1,25-(OH)_2_-D_3_] and 1α,25-dihydroxy- vitamin-D_2_ [d6-1,25-(OH)_2_-D_2_] in acetonitrile. Calibration mix solutions were similarly prepared by dissolving known amounts of 1,25-(OH)_2_-D_3_ and 1,25-(OH)_2_-D_2_ in acetonitrile. The final concentrations of the calibration mixes were 0, 15.63, 31.25, 62.5, 125, and 250 pg/mL.

Plasma samples, calibration matrices, and reference standards were prepared by equilibration, vortexing, and aliquoting. Samples were diluted with deionized water, vortexed, centrifuged, and spiked with an internal standard. Calibration mixes were added to the top cartridge. Samples were loaded and allowed to sit for 10 minutes. The top cartridge was eluted with di-isopropyl ether. The silica cartridge was washed with isopropyl alcohol in hexanes to remove impurities. Analytes were eluted with isopropyl alcohol in hexanes. Samples were dried, reconstituted with methanol, and transferred to smaller vials. Amplifex reagent was diluted with reagent diluent to a concentration of 1 to 1.5 mg/mL. Thirty microliters of the diluted Amplifex reagent were added to each dried sample, vortexed, centrifuged, and incubated for 30 minutes to 1 hour at ambient temperature. Deionized water were then added to each tube, vortexed, centrifuged, and transferred for LC injection.

Derivatized extracts were analyzed using a Shimadzu Prominence (Addison, IL, USA) integrated HPLC system coupled with an AB SCIEX (Foster City, CA) QTRAP 5500 mass spectrometer (Framingham, MA, USA). A Phenomenex Kinetex analytical column was used with a binary reversed-phase gradient elution. The mobile phase consisted of 0.1% formic acid in water (Channel A) and methanol (Channel B). The flow rate was 0.25 mL/min. The mass spectrometer operated in positive TurboIonSpray mode. Relevant MS/MS settings included a CAD gas pressure of 6 psig, CUR of 20 psig, GS1 of 60 psig, GS2 of 30 psig, IS of 5000 V, EP of 10 V, and TEM of 600 ℃. The limit of detection for 1,25-(OH)_2_-D_3_ was 2.0 pg/L. Calibration curves were constructed using peak area ratios of 1,25-(OH)_2_-D_3_ to d6-1,25-(OH)_2_-D_2_ and linear regression. Quality assurance/quality control procedures included running calibration blanks, standards, and triplicate spikes of 50 µg/L 1,25-(OH)_2_-D_3_.

1. **MASLD mouse model**

  MASLD mouse model was constructed as described previously with minor modification^13^. Five-week-old wild-type mice were fed either a Western diet or a methionine choline-deficient diet for five weeks to induce MASLD models. WT mice fed normal diet for 5 weeks were set as healthy negative controls. The MASLD mouse model was verified by H&E staining and Oil Red O staining.

1. **H&E, Oil Red O Staining**

H&E and Oil Red O staining were performed as previously described^13^. Cryosections were stained with H&E according to a standard protocol. Frozen tissue sections were stained with Oil Red O for lipid detection following the manufacturer’s instructions. Photographs were taken with the microscope (OLYMPUS, BX43F) and software (XV Image Processing, 3.15.0.15404). Every index was detected a minimum of three times.

**31. The serum biochemistry assay**

Serum samples were collected from each group of mice and analyzed for alanine aminotransferase (ALT), aspartate aminotransferase (AST), alkaline phosphatase (ALP), total cholesterol (TC), triglycerides (TG), low-density lipoprotein cholesterol (LDL-C), high-density lipoprotein cholesterol (HDL-C), and glucose at the Department of Clinical Laboratory, Zhongshan Hospital, Fudan University (Shanghai, China), using standard laboratory procedures.

**32. Immunohistochemistry of liver**

Immunohistochemical analysis was performed using the UltraSensitiveTM SP (Mouse/Rabbit) IHC Kit (Maixin Biotech; #KIT-9730), which employs a streptavidin-biotin amplification method. Briefly, 4 µm-thick liver tissue sections were deparaffinized, rehydrated, and subjected to antigen retrieval using a citrate solution in a pressure cooker at 121℃ for 5 minutes. Slides were incubated with 3% hydrogen peroxide and blocking buffer for 5 and 30 minutes, respectively, followed by an overnight incubation with the primary antibody anti-CD41 (Abcam; #ab134131) at 4℃. After washing with PBS, biotin-conjugated secondary antibody and streptavidin-conjugated tertiary antibody were added sequentially for 30 and 20 minutes, respectively. Positive signals were visualized with 3,3’-diaminobenzidine solution. Images were captured with an Olympus BX43F microscope and XV Image Processing software (version 3.15.0.15404). Average integrated optical density values were quantified by analyzing five random fields per slide using Image-Pro Plus software version 5.0.

1. **Pulmonary embolism model**

A pulmonary embolism model was established as previously described^14,15^. Mice were anesthetized with an intraperitoneal injection of 1% pentobarbital sodium (10 mL/kg body weight). A mixture of collagen (0.3 μg/g body weight) and epinephrine (0.06 μg/g body weight) in 100 μL double-distilled water was injected into the tail vein. Five minutes post-injection, lung tissues were excised, imaged, and fixed in 4% formalin. The tissues were then dehydrated in 70% ethyl alcohol, embedded in paraffin, and sectioned at 5 μm thickness. Platelets were stained overnight at 4℃ with anti-CD41 antibody (1:50, Abcam). Slides were then washed, incubated with biotin-conjugated anti-rabbit IgG, avidin-linked enzyme peroxidase complex, and DAPI at room temperature for 2 hours. The number of pulmonary emboli per field of view was counted.

1. **Myocardial I/R model**

Surgical induction of myocardial I/R was established using a previously described methodology^16^. Surgery was performed by an investigator who was blinded to the treatment groups, ensuring unbiased procedures. Mice were anesthetized with 2% isoflurane and mechanically ventilated with a rodent respirator (inspiratory tidal volume of 250 μl at 130 breaths per min). A left thoracotomy was performed in the fourth intercostal space to expose the heart. Myocardial ischemia was induced by placing a 6.0 silk suture slipknot around the left anterior descending (LAD) coronary artery at its emergence site. Regional ischemia was confirmed by monitoring ST-segment elevation on electrocardiography. The slipknot was released to facilitate coronary artery reperfusion after 45 minutes of temporary LAD coronary artery ligation.

1. **Measurement of area at risk and infarct area**

The infarct size after I/R injury was determined as previously described with minor modifications^16^. Briefly, after 48 hours of reperfusion, mice were anesthetized, and the LAD artery was occluded with the same suture at the ligation site. 1% Evans blue dye (Sigma-Aldrich, St Louis, MO) was then injected into the left ventricular (LV) cavity. The heart was quickly excised and washed in saline. The ventricles were frozen at -20℃ for 1 hour, sliced at a thickness of 1 mm, and incubated in 1% 2,3,5-triphenyltetrazolium chloride (TTC; Sigma-Aldrich) solution at 37℃ for 15 minutes. The hearts were then fixed in 4% paraformaldehyde. Parasternal short-axis sections were cut from the harvested hearts. Slices were photographed and analyzed by ImageJ software (NIH, Bethesda, MD, USA) to quantify LV area, area at risk (AAR), and infarcted area. The AAR is defined as the region unstained by Evans blue dye, while the infarcted area is defined as the area uncolored by TTC (white area).

1. **Immunohistochemistry in myocardial I/R mice**

The mouse hearts were harvested after one week of reperfusion and fixed in 4% formalin. The tissues were then dehydrated in 70% ethyl alcohol, embedded in paraffin, and cut into 5 μm thick sections. Platelets were stained overnight at 4℃ with anti-CD41 antibody (1:50, Abcam). The slides were then washed and incubated with biotin-conjugated anti-rabbit IgG, avidin-linked enzyme peroxidase complex, and DAPI at room temperature for 2 hours. The stained slides were photographed with an Olympus microscope. The positive staining in each section was quantified using ImageJ software.

1. **Echocardiography**

Mouse echocardiography was conducted using a previously described protocol^17^. Cardiac structure and function were evaluated using a Vevo 2100 ultrasound imaging system (Visual Sonics, Toronto, Canada) equipped with a 30-megahertz linear-array transducer (MS-550D). Mice were anesthetized with 2% isoflurane and positioned supine. After chest preparation, parasternal long-axis (PSLAX) and three orthogonal short-axis (SAX) (mid-ventricular, apical, and basal) brightness (B)-mode echocardiographic images were acquired by a blinded investigator. Post-acquisition analysis was performed by the same investigator in a blinded manner. Volume calculation using the biplane Simpson's method was performed based on ventricular diastolic and systolic lengths derived from PSLAX B-mode imaging, and endocardial tracings from three SAX B-mode views at both diastole and systole^18^ (*Supplementary Figure 27*). The volume (V) was calculated using the formula: V = (area mid-ventricular + area apical + area basal) * h/3, where h represents ventricular length. The left ventricular ejection fraction (LVEF) was calculated using the formula: LVEF = (EDV - ESV) / EDV * 100. M-mode analysis was also used to measure LV systolic and diastolic function. By tracing the endocardium at end-diastole and end-systole, the left ventricular internal dimensions (LVIDd and LVIDs) were measured. The left ventricular end-diastolic volume (LVEDV) and left ventricular end-systolic volume (LVESV) were calculated using the Vevo 2100 software. The LVEF was calculated using the formula: LVEF = (LVEDV - LVESV)/LVEDV × 100%. All measurements were averaged over 10 consecutive cardiac cycles.

1. **MCAO model**

An MCAO model was established following a previously described protocol with minor modifications^19,20^. Mice were anesthetized using an intraperitoneal injection of 1.25% tribromoethanol at a dose of 0.2 mL per 10 grams body weight. After careful dissection, the right internal, external, and common carotid arteries were ligated. A nylon suture was inserted into the internal carotid artery bifurcation via the external carotid artery, inducing an ischemic period of 1 hour. Reperfusion was achieved by removing the suture and releasing the ligature on the common carotid arteries. The infarct volume was measured 24 hours after reperfusion. The brains were removed, rinsed in saline, and frozen at -20℃. Slices were cut to a thickness of 2 mm and incubated in TTC solution. The infarct area (unstained region) was quantified using ImageJ software (NIH, Bethesda, MD, USA).

1. **Statistical analysis**

Categorical variables are presented using counts and percentages and continuous variables as the mean ± standard deviation (SD) or median (25th and 75th percentiles [(Q1, Q3]) based on data distribution. Normality was assessed using the Shapiro-Wilk test. For normally distributed data with a single variable, differences between two groups were compared using an unpaired Student’s *t*-test. For non-normally distributed data, the Mann-Whitney *U* test was used. Comparisons among more than two groups with one variable were performed using one-way analysis of variance (ANOVA) with Dunnett’s (comparing the mean of each column with the mean of a control column) or Sidak’s (comparing the means of preselected pairs of columns) multiple comparison tests for independent data. For normally distributed data with two variables, differences were evaluated using two-way ANOVA with Tukey’s multiple comparisons test. For non-normally distributed data, the Kruskal-Wallis test was used. The sample size for the calcitriol intervention study was calculated using a previously published methodology^21^. The paired samples *t*-test was used to compare change from baseline for continuous variables. The correlation between the platelet aggregation ratio and plasma calcitriol levels was investigated using the nonparametric Spearman’s rank correlation test (r). The sample size was determined by consulting a sample size table^22^. For all statistical analyses, *P*<0.05 was considered statistically significant. Statistical analyses were conducted using GraphPad Prism version 8.0 (GraphPad Inc., San Diego, CA, USA).

**References**

1. Bujang M A, Baharum N. Sample size guideline for correlation analysis. *World Journal of Social Science Research* 2016; **3**:37-46. https://doi.org/10.22158/wjssr.v3n1p37
2. Zaheer, S., Taquechel, K., Brown, J. M., Adler, G. K., Williams, J. S., Vaidya, A. A randomized intervention study to evaluate the effect of calcitriol therapy on the renin-angiotensin system in diabetes. *J Renin Angiotensin Aldosterone Syst* 2018; **19**:1470320317754178. https://doi.org/10.1177/1470320317754178
3. Sultan, M., Twito, O., Tohami, T., Ramati, E., Neumark, E., Rashid, G. Vitamin D diminishes the high platelet aggregation of type 2 diabetes mellitus patients. *Platelets* 2019; **30**:120-125. https://doi.org/10.1080/09537104.2017.1386298
4. Chew NWS, Mehta A, Goh RSJ, Zhang A, Chen Y, Chong B, Chew HSJ, Shabbir A, Brown A, Dimitriadis GK, et al. Cardiovascular-Liver-Metabolic Health: Recommendations in Screening, Diagnosis, and Management of Metabolic Dysfunction-Associated Steatotic Liver Disease in Cardiovascular Disease via Modified Delphi Approach. *Circulation* 2025;**151**: 98-119. doi: 10.1161/CIRCULATIONAHA.124.070535.
5. Chen, Y., Hong, J., Zhong, H., Zhao, Y., Li, J., Shen, W., et al. IL-37 Attenuates Platelet Activation and Thrombosis Through IL-1R8 Pathway. *Circ Res* 2023; **132**:e134-e150. https://doi.org/10.1161/CIRCRESAHA.122.321787
6. Qi, Z., Hu, L., Zhang, J., Yang, W., Liu, X., Jia, D., et al. PCSK9 (Proprotein Convertase Subtilisin/Kexin 9) Enhances Platelet Activation, Thrombosis, and Myocardial Infarct Expansion by Binding to Platelet CD36. *Circulation* 2021; **143**:45-61. https://doi.org/10.1161/CIRCULATIONAHA.120.046290
7. Schmitz, B., Radbruch, A., Kümmel, T., Wickenhauser, C., Korb, H., Hansmann, M. L., et al. Magnetic activated cell sorting (MACS)--a new immunomagnetic method for megakaryocytic cell isolation: comparison of different separation techniques. *Eur J Haematol* 1994; **52**:267-275. https://doi.org/10.1111/j.1600-0609.1994.tb00095.x
8. Zhu, W., Gregory, J. C., Org, E., Buffa, J. A., Gupta, N., Wang, Z., et al. Gut Microbial Metabolite TMAO Enhances Platelet Hyperreactivity and Thrombosis Risk. *Cell* 2016; **165**:111-124. https://doi.org/10.1016/j.cell.2016.02.011
9. Woulfe, D., Jiang, H., Morgans, A., Monks, R., Birnbaum, M., Brass, L. F. Defects in secretion, aggregation, and thrombus formation in platelets from mice lacking Akt2. *J Clin Invest* 2004; **113**:441-450. https://doi.org/10.1172/JCI20267
10. Johnson, E. N., Brass, L. F., Funk, C. D. Increased platelet sensitivity to ADP in mice lacking platelet-type 12-lipoxygenase. *Proc Natl Acad Sci U S A* 1998; **95**:3100-3105. https://doi.org/10.1073/pnas.95.6.3100
11. Dong, Z., Hou, L., Luo, W., Pan, L. H., Li, X., Tan, H. P., et al. Myocardial infarction drives trained immunity of monocytes, accelerating atherosclerosis. *Eur Heart J* 2024; **45**:669-684. https://doi.org/10.1093/eurheartj/ehad787
12. Hedman, C. J., Wiebe, D. A., Dey, S., Plath, J., Kemnitz, J. W., Ziegler, T. E. Development of a sensitive LC/MS/MS method for vitamin D metabolites: 1,25 Dihydroxyvitamin D2&3 measurement using a novel derivatization agent. *J Chromatogr B Analyt Technol Biomed Life Sci* 2014; **953**:62-67. https://doi.org/10.1016/j.jchromb.2014.01.045
13. Jin K, Shi Y, Zhang H, Zhangyuan G, Wang F, Li S, et al. A TNFα/Miz1-positive feedback loop inhibits mitophagy in hepatocytes and propagates non-alcoholic steatohepatitis. *J Hepatol* 2023; **79**:403-416. https://doi.org/10.1016/j.jhep.2023.03.039
14. Shi J, Tong R, Zhou M, Gao Y, Zhao Y, Chen Y, et al. Circadian nuclear receptor Rev-erbα is expressed by platelets and potentiates platelet activation and thrombus formation. *Eur Heart J* 2022; **43**:2317-2334. https://doi.org/10.1093/eurheartj/ehac109
15. Sun X, Zhou M, Pu J, Wang T. Stachydrine exhibits a novel antiplatelet property and ameliorates platelet-mediated thrombo-inflammation. *Biomed Pharmacother* 2022; **152**:113184. https://doi.org/10.1016/j.biopha.2022.113184
16. Pang A, Cheng N, Cui Y, Bai Y, Hong Z, Delaney MK, et al. High-loading Gα13-binding EXE peptide nanoparticles prevent thrombosis and protect mice from cardiac ischemia/reperfusion injury. *Sci Transl Med* 2020; **12**:eaaz7287. https://doi.org/10.1126/scitranslmed.aaz7287
17. Lindsey ML, Kassiri Z, Virag JAI, de Castro Brás LE, Scherrer-Crosbie M. Guidelines for measuring cardiac physiology in mice. *Am J Physiol Heart Circ Physiol* 2018; **314**:H733-H752. https://doi.org/10.1152/ajpheart.00339.2017

18 . Heinen, A., Raupach, A., Behmenburg, F., Hölscher, N., Flögel, U., Kelm, M., et al. Echocardiographic analysis of cardiac function after infarction in mice: validation of single-plane long-axis view measurements and the bi-plane simpson method. *Ultrasound Med Biol* 2018; **44**:1544-1555. https://doi.org/10.1016/j.ultrasmedbio.2018.03.020

19. Gotru SK, Mammadova-Bach E, Sogkas G, Schuhmann MK, Schmitt K, Kraft P, et al. MAGT1 Deficiency Dysregulates Platelet Cation Homeostasis and Accelerates Arterial Thrombosis and Ischemic Stroke in Mice. *Arterioscler Thromb Vasc Biol* 2023; **43**:1494-1509. https://doi.org/10.1161/ATVBAHA.122.318115

20. Zhang F, Deng Y, Wang H, Fu J, Wu G, Duan Z, et al. Gut microbiota-mediated ursodeoxycholic acids regulate the inflammation of microglia through TGR5 signaling after MCAO. *Brain Behav Immun* 2024; **115**:667-679. https://doi.org/10.1016/j.bbi.2023.11.021

21. Nanjundeswaraswamy T S, Divakar S. Determination of sample size and sampling methods in applied research. *Proceedings on engineering sciences* 2021; **3**:25-32. https://doi.org/10.24874/PES03.01.003

22. Pilz S, Verheyen N, Grübler MR, Tomaschitz A, März W. Vitamin D and cardiovascular disease prevention. Nat Rev Cardiol 2016; 13:404-417. https://doi.org/10.1038/nrcardio.2016.73

**Supplementary Tables**

| Variable | Value | Reference range |
| --- | --- | --- |
| Age (years) | 43.0 [35.8, 50.0] | - |
| Male sex, n (%) | 67 (56.8%) | - |
| BMI (kg/m^2^) | 23.9 ± 3.8 | 18.5-23.9 |
| FBG (mmol/L) | 4.9 ± 0.7 | 3.9-6.1 |
| HbA1c (%) | 5.5 ± 0.6 | 4.0-6.0 |
| TC (mmol/L) | 3.8 [3.1, 4.8] | < 5.2 |
| TG (mmol/L) | 1.0 [0.7, 1.4] | < 1.7 |
| LDL (mmol/L) | 2.2 [1.6, 3.0] | < 3.4 |
| HDL (mmol/L) | 1.1 [0.8, 1.3] | > 1.0 |
| ALT (U/L) | 22.5 [16.0, 33.3] | 7-40 |
| AST (U/L) | 22.0 [17.0, 34.0] | 13-35 |
| ALP (U/L) | 72.2 ± 11.0 | 35-130 |
| Creatinine (µmol/L) | 71.0 [61.0, 78.0] | 41-97 |
| Platelet count (×10^9^) | 216.0 ± 43.3 | 100-300 |

**Supplementary Table 1. Characteristics of healthy participants enrolled in the study (n=118).**

Data are presented as mean ± SD for continuous variables with normal distribution, median [25th, 75th percentiles] for continuous variables with non-normal distribution, and n (%) for categorical variables.

BMI: body mass index; FBG: fasting blood glucose; HbA1c: Hemoglobin A1c; TC: total cholesterol; TG: triglyceride; LDL-C: low density lipoprotein cholesterol; HDL-C: high density lipoprotein cholesterol; ALT: alanine aminotransferase; AST: glutamic oxaloacetic transaminase; ALP: alkaline phosphatase.

| Variable | Value | Reference range |
| --- | --- | --- |
| Age (years) | 27.0 ± 2.6 | - |
| Male sex, n (%) | 4 (40.0 %) | - |
| BMI (kg/m^2^) | 23.3 ± 1.8 | 18.5-23.9 |
| Smoking, n (%) | 0 (0.0%) | - |
| Hypertension, n (%) | 0 (0.0%) | - |
| Diabetes mellitus, n (%) | 0 (0.0%) | - |
| Platelet count ( ×10^9^) | 233.9 ± 33.1 | 100-300 |
| Plasma vitamin D (ng/mL) | 24.6 ± 3.8 | 30-100 |

**Supplementary Table 2. Characteristics of the healthy participants for calcitriol intervention (n=10).**

Data are presented as mean ± SD for continuous variables with normal distribution, median [25th, 75th percentiles] for continuous variables with non-normal distribution, and n (%) for categorical variables.

BMI: body mass index.

**Supplementary Table 3. Baseline characteristics of healthy participants and MASLD patients.**

| Variable | Healthy  (n=118) | MASLD  (n=89) | *P* value | Reference range |
| --- | --- | --- | --- | --- |
| Age (years) | 43.0 [35.8, 50.0] | 43.0 [32.0, 58.5] | 0.50^#^ | - |
| Male sex, n (%) | 67 (56.8%) | 53 (59.6%) | 0.69^*^ | - |
| Creatinine (µmol/L) | 71.0 [61.0, 78.0] | 72.0 [58.0, 81.0] | 0.29^#^ | 41-97 |
| Platelet count ( ×10^9^) | 216.0 ± 43.3 | 212.7 ± 55.4 | 0.41^Δ^ | 100-300 |

Data are presented as mean ± SD for continuous variables with normal distribution, median [25th, 75th percentiles] for continuous variables with non-normal distribution, and n (%) for categorical variables. Data were analyzed by Chi-square test (*), unpaired Student’s *t*-test (Δ), and Mann-Whitney *U* test (#).

MASLD: metabolic dysfunction-associated steatotic liver disease.

**Supplementary Table 4. Characteristics of MASLD patients enrolled in the study (n=89).**

| Variable | Value | Reference range |
| --- | --- | --- |
| Age (years) | 43.0 [32.0, 58.5] | - |
| Male sex, n (%) | 53 (59.6%) | - |
| Hypertension, n (%) | 36 (40.5%) | - |
| BMI (kg/m^2^) | 27.9 [25.1, 31.1] | 18.5-23.9 |
| FBG (mmol/L) | 4.9 [4.2, 5.5] | 3.9-6.1 |
| HbA1c (%) | 5.6 [5.2, 7.4] | 4.0-6.0 |
| TC (mmol/L) | 4.4 ± 1.0 | < 5.2 |
| TG (mmol/L) | 1.1 [1.1, 2.0] | < 1.7 |
| LDL (mmol/L) | 2.6 ± 0.8 | < 3.4 |
| HDL (mmol/L) | 1.1 [0.9, 1.2] | > 1.0 |
| ALT (U/L) | 26.0 [14.0, 44.0] | 7-40 |
| AST (U/L) | 19.0 [14.0, 29.5] | 13-35 |
| ALP (U/L) | 75.0 [62.5, 83.5] | 35-130 |
| Creatinine (µmol/L) | 72.8 ± 17.3 | 41-97 |
| Platelet count ( ×10^9^) | 212.7 ± 55.4 | 100-300 |

Data are presented as mean ± SD for continuous variables with normal distribution, median [25th, 75th percentiles] for continuous variables with non-normal distribution, and n (%) for categorical variables.

MASLD: metabolic dysfunction-associated steatotic liver disease; BMI: body mass index; FBG: fasting blood glucose; HbA1c: Hemoglobin A1c; TC: total cholesterol; TG: triglyceride; LDL-C: low density lipoprotein cholesterol; HDL-C: high density lipoprotein cholesterol; ALT: alanine aminotransferase; AST: glutamic oxaloacetic transaminase; ALP: alkaline phosphatase.

**Supplementary Table 5.Characteristics of the MASLD patients for calcitriol intervention (n=10).**

| Variable | Value | Reference range |
| --- | --- | --- |
| Age (years) | 27.4 ± 4.1 | - |
| Male sex, n (%) | 5 (50.0 %) | - |
| BMI (kg/m^2^) | 25.5 ± 1.0 | 18.5-23.9 |
| Smoking, n (%) | 0 (0.0%) | - |
| Hypertension, n (%) | 4 (0.0%) | - |
| Diabetes mellitus, n (%) | 3 (0.0%) | - |
| Anti-platelet drugs, n (%) | 0 (0.0%) | - |
| Platelet count ( ×10^9^) | 223.1 ± 29.3 | 100-300 |
| Plasma vitamin D (ng/mL) | 13.9 ± 3.1 | 30-100 |

Data are presented as mean ± SD for continuous variables with normal distribution, median [25th, 75th percentiles] for continuous variables with non-normal distribution, and n (%) for categorical variables.

BMI: body mass index; MASLD: metabolic dysfunction-associated steatotic liver disease.

| Name | Assay | Cat# | Company |
| --- | --- | --- | --- |
| P-selectin | Flow cytomety | 550888 | BD Biosciences |
| PAC-1 | Flow cytomety | MA5-28564 | Invitrogen |
| Anti-human CD41 | Flow cytomety | 303706 | Biolegend |
| CD62P | Flow cytomety | 148304 | Biolegend |
| JoN/A | Flow cytomety | #M023-2 | Emfret |
| Anti-mouse CD41 | Flow cytomety | 133904 | Biolegend |
| Anti-human P2Y_12_ | Flow cytomety | 848006 | Biolegend |
| APC Rat IgG2b | Flow cytomety | 400611 | Biolegend |
| VDR | ChIP | AB109234 | Abcam |
| Isotype control IgG | ChIP | AB171870 | Abcam |
| VDR | COIP/WB | sc-13133 | Santa Cruz Biotechnology |
| RXR | COIP | sc-515928 | Santa Cruz Biotechnology |
| Isotype mouse IgG1 | COIP | sc-2855 | Santa Cruz Biotechnology |
| CD45 | WB | 20103-1-AP | Proteintech |
| P2Y_12_ | WB | APR-012 | Alomone labs |
| P-PKA | WB | 9624S | Cell Signaling Technology |
| P-PKC | WB | 2261S | Cell Signaling Technology |
| p-ERK1/2 (Thr202/Tyr204) | WB | #4370 | Cell Signaling Technology |
| ERK1/2 | WB | 16443-1-AP | Proteintech |
| p-JNK (Tyr185) | WB | 80024-1-RR | Proteintech |
| JNK | WB | 24164-1-AP | Proteintech |
| p-p38 (Thr180/Tyr182) | WB | #4511 | Cell Signaling Technology |
| p38 | WB | 14064-1-AP | Proteintech |
| GAPDH | WB | 60004-1-Ig | Proteintech |
| Goat Anti-Mouse IgG (H+L) | WB | SA00001-1 | Proteintech |
| Goat Anti-Rabbit IgG (H+L) | WB | SA00001-2 | Proteintech |
| CD41 | IF | AB134131 | Abcam |
| DAPI | IF | C1002 | Beyotime |
| Rabbit anti-mouse thrombocyte | Eliminate platelet | J1943 | Accurate Chemical |

**Supplementary Table 6. Antibodies used.**

**Supplementary Table 7. Primers used.**

| Name | 5’ to 3’ | experiments | species |
| --- | --- | --- | --- |
| GAPDH-F | ACCTGCCAAGTATGATGAC | RT-RCR | mouse |
| GAPDH-R | CTGTTGCTGTAGCCGTAT | RT-RCR | mouse |
| CD45-F | GGATGTCTATGGTTATGTTGTC | RT-RCR | mouse |
| CD45-R | GTATGAAGGAAGTCTCTGGTAT | RT-RCR | mouse |
| VDR-F | ATGAAGCGGAAGGCACTA | RT-RCR | mouse |
| VDR-R | GCAGAAGTCGGAGTAGGT | RT-RCR | mouse |
| P2Y_12_-F | TCACCCAGGTCCTCTTCCC | qPCR | mouse |
| P2Y_12_-R | TGTTCCCAGTTTGGCATCAC | qPCR | mouse |
| P2Y_1_-F | GAGGTGCCTTGGTCGGTTG | qPCR | mouse |
| P2Y_1_-R | CGGCAGGTAGTAGAACTGGAA | qPCR | mouse |
| PAR4-F | TGCTGTATCCTTTGGTGCTGG | qPCR | mouse |
| PAR4-R | CGTTGGCACAGAATTTGCCC | qPCR | mouse |
| GP IV-F | CTTCCCTGCTTGGCCACATA | qPCR | mouse |
| GP IV-R | GGGCCACTCTGTGTTTGGAT | qPCR | mouse |
| P2Y_12_-F | TCGTAGAGACCTGGAGAC | ChIP | mouse |
| P2Y_12_-R | CACACTGCTTAGATGAACAT | ChIP | mouse |
| GAPDH-F | ACATCGCTCAGACACCATG | qPCR | human |
| GAPDH-R | TGTAGTTGAGGTCAATGAAGGG | qPCR | human |
| P2Y_12_-F | TCACCCAGGTCCTCTTCCC | qPCR | human |
| P2Y_12_-R | TGTTCCCAGTTTGGCATCAC | qPCR | human |
| P2Y_12_-F | GATCGCTTGTCTCCTAGCTCTT | ChIP | human |
| P2Y_12_-R | TTGTTGTGTAACAACAGTGCCT | ChIP | human |

**Supplementary Figures**

**
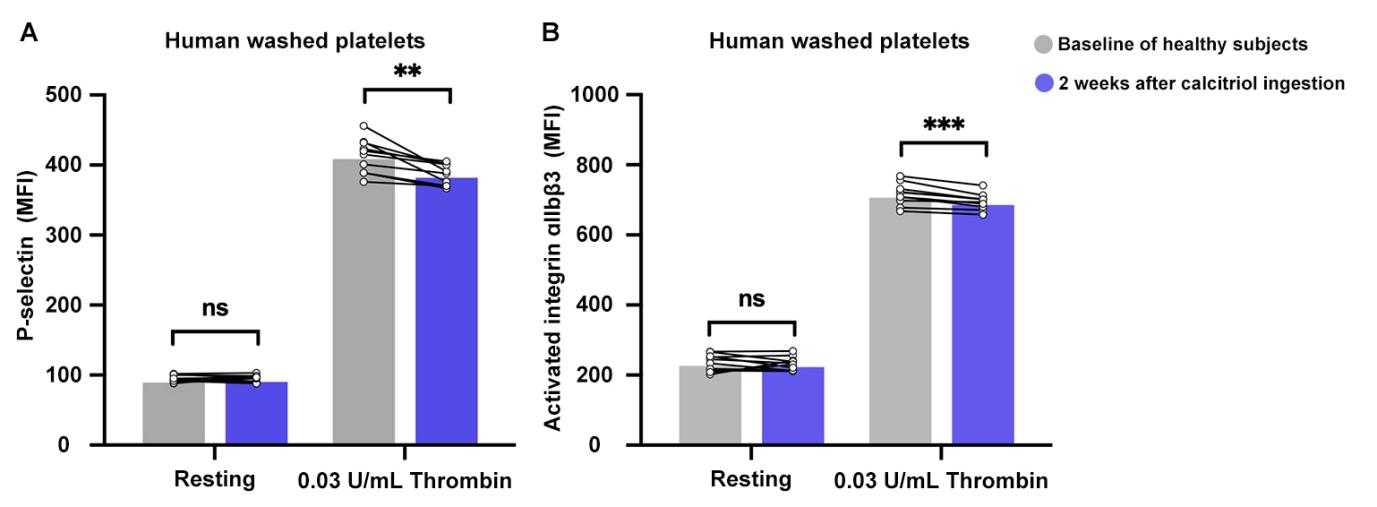
**

**Supplementary Figure 1. Ingestion of calcitriol inhibits human platelet P-selectin expression and integrin αIIbβ3 activation.**

**(A)** Thrombin-induced (0.03 U/mL) P-selectin surface expression on platelets from healthy volunteers before and after calcitriol ingestion (n=10, 4 males). **(B)** PAC1 binding to human platelets stimulated with 0.03 U/mL thrombin from healthy volunteers before and after calcitriol ingestion (n=10, 4 males). Data were analyzed by paired samples *t*-test in **(A)** and **(B)**. Data are presented as mean ± SD. **P<0.01; ***P<0.001; ns, not significant.


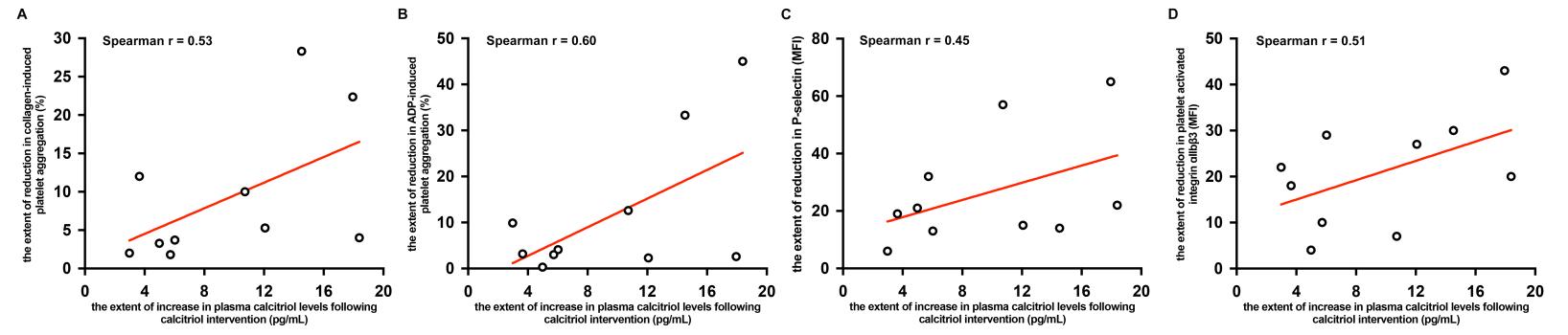


**Supplementary Figure 2. Correlation between the elevation of plasma calcitriol levels and the reduction of platelet reactivity in healthy individuals.**

**(A)** Correlation between the extent of increase in plasma calcitriol levels and the extent of reduction in collagen-induced platelet aggregation. **(B)** Correlation between the extent of increase in plasma calcitriol levels and the extent of reduction in ADP-induced platelet aggregation. **(C)** Correlation between the extent of increase in plasma calcitriol levels and the extent of reduction in surface P-selectin expression. **(D)** Correlation between the extent of increase in plasma calcitriol levels and the extent of reduction in platelet activated integrin αIIbβ3 expression. Spearman r values are indicated on each plot. Data were analyzed by Spearman's rank correlation test. ADP, adenosine diphosphate; MFI, mean fluorescence intensity.


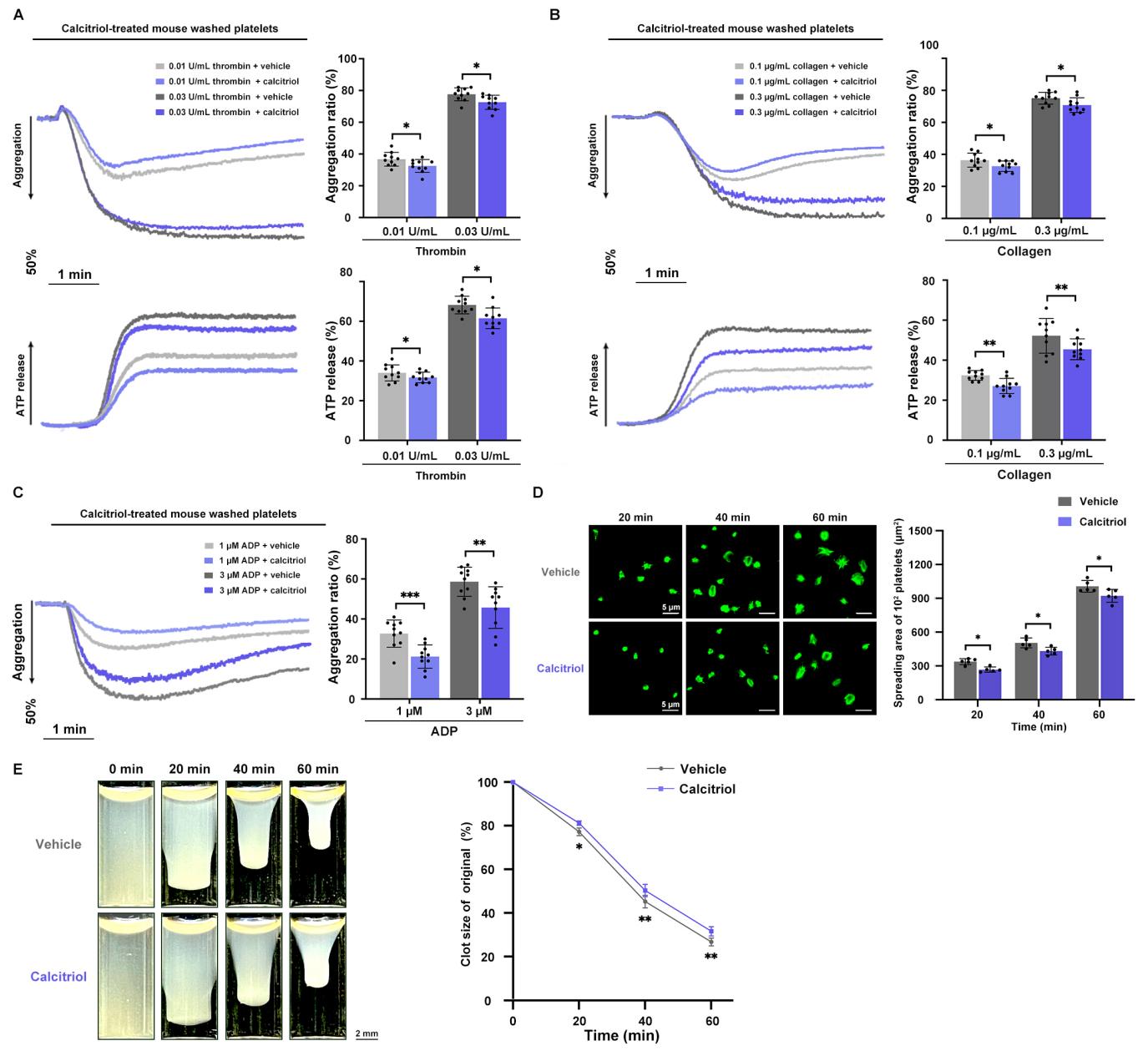


**Supplementary Figure 3.** **Ingestion of calcitriol inhibits mouse platelet activation.**

**(A-B)** Representative traces and summary data illustrating platelet aggregation and ATP release induced by 2 distinct concentrations of thrombin **(A)** or collagen **(B)** in washed mouse platelets from calcitriol-treated and control WT mice (n=10). **(C)** Representative recordings and summary data of platelet aggregation in washed mouse platelets from calcitriol-treated and control WT mice, stimulated with 2 different concentrations of ADP (n=10). **(D)** Representative images and quantitative analysis of platelet spreading on immobilized fibrinogen were performed at various time points (n=5) to compare platelets from calcitriol-treated mice with control WT mice. One hundred randomly selected platelets were measured for each condition. **(E)** Representative images and quantitative analysis of clot retraction in washed platelets from calcitriol-treated and control WT mice (n=5). Data were analyzed by one-way ANOVA with Sidak’s multiple comparisons in **(A)**-**(C)**, two-way ANOVA with Tukey’s multiple comparisons test in **(D)**-**(E)**. Data are presented as mean ± SD. *P<0.05; **P<0.01; ***P<0.001. ADP, adenosine diphosphate; ATP, adenosine triphosphate; WT, wild-type.

**
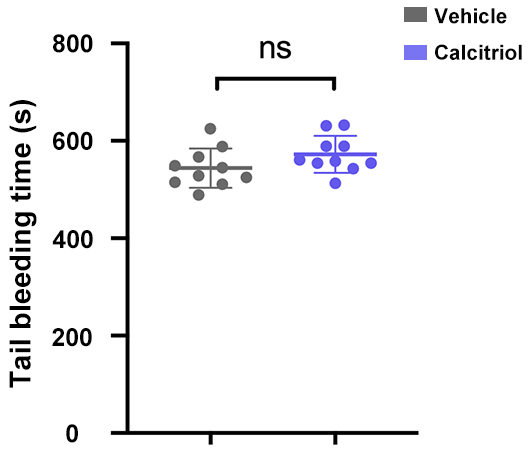
**

**Supplementary Figure 4. Calcitriol treatment does not significantly affect bleeding time.**

Tail bleeding time in calcitriol-treated WT mice and vehicle-treated WT mice (n=10). Data were analyzed by unpaired Student’s *t*-test. Data are presented as mean ± SD. ns, not significant. WT, wild-type.

**
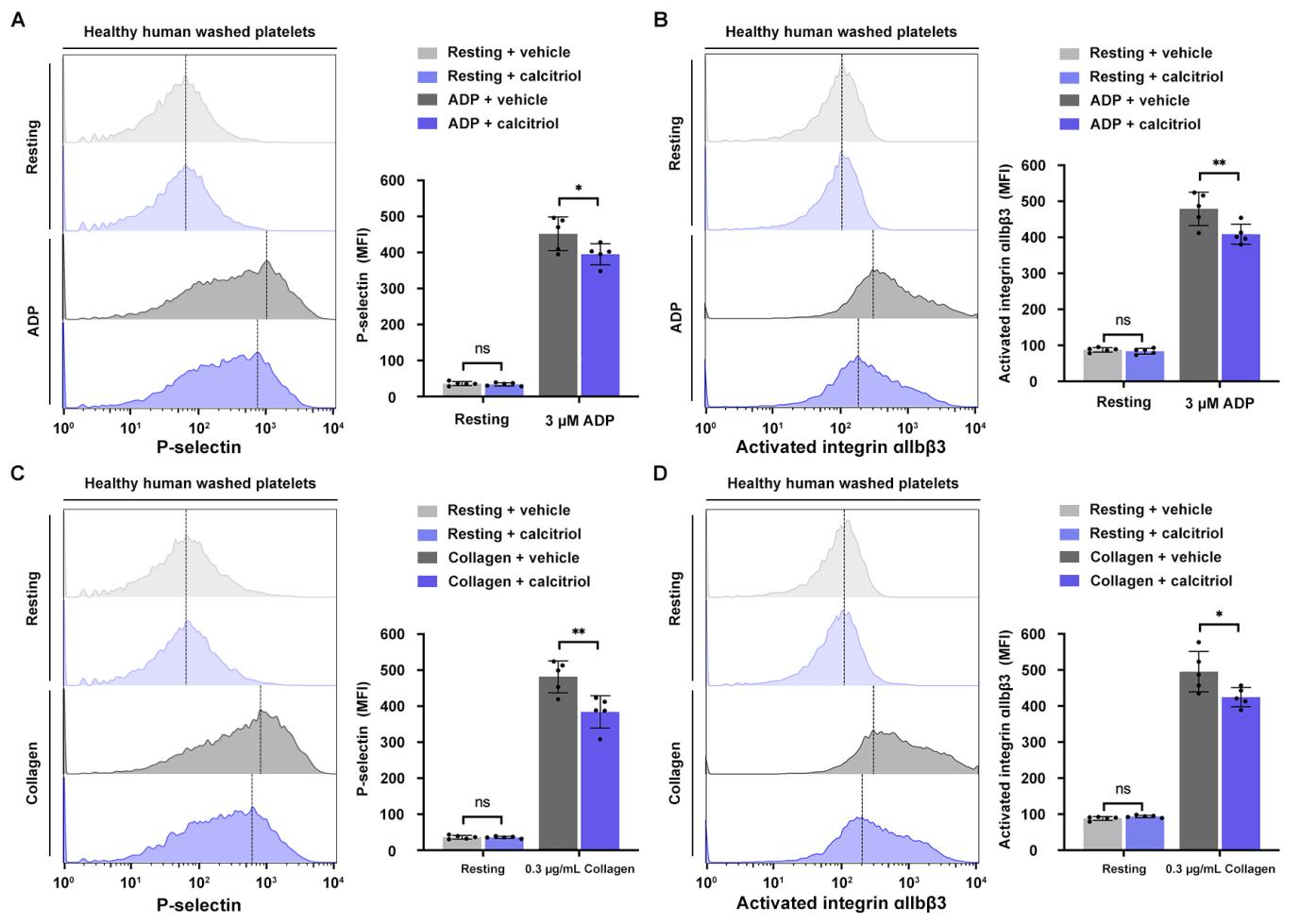
**

**Supplementary Figure 5. Calcitriol significantly reduced P-selectin release and platelet integrin αIIbβ3 activation in healthy human platelets stimulated with ADP or collagen.**

1. Evaluation of P-selectin surface expression on washed human platelets isolated from healthy volunteers, pretreated with 40 pg/mL calcitriol or vehicle and subsequently stimulated with 3 µM ADP (n=5). **(B)** Assessment of PAC1 binding to washed human platelets isolated from healthy volunteers, pretreated with 40 pg/mL calcitriol or vehicle control, and subsequently stimulated with 3 µM ADP (n=5). **(C)** P-selectin surface expression was evaluated on washed human platelets from healthy volunteers pretreated with 40 pg/mL calcitriol or vehicle and subsequently stimulated with 0.3 µg/mL collagen (n=5). **(D)** PAC1 binding was compared between washed human platelets from healthy volunteers pretreated with 40 pg/mL calcitriol or vehicle and subsequently stimulated with 0.3 µg/mL collagen (n=5). Data were analyzed by one-way ANOVA with Sidak’s multiple comparisons in **(A)**-**(D)**. Data are presented as mean ± SD. *P<0.05; **P<0.01; ns, not significant. ADP, adenosine diphosphate.


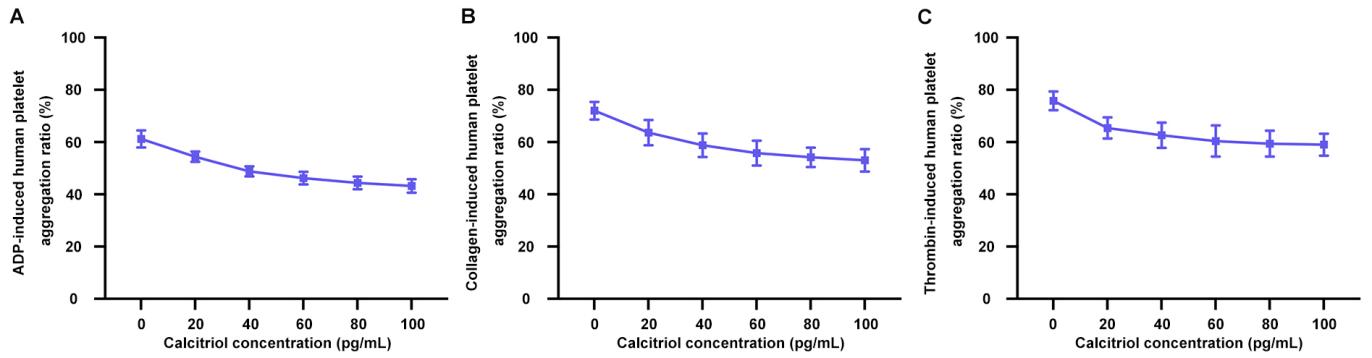


**Supplementary Figure 6**. Calcitriol dose titration for human platelet function.

(A-C) The association between calcitriol concentration and washed human platelet aggregation induced by ADP (3 μM) (A), collagen (0.3 μg/mL) (B), or thrombin (0.03 U/mL) (C). Summary data are presented (n=5, each concentration point). ADP, adenosine diphosphate.

**
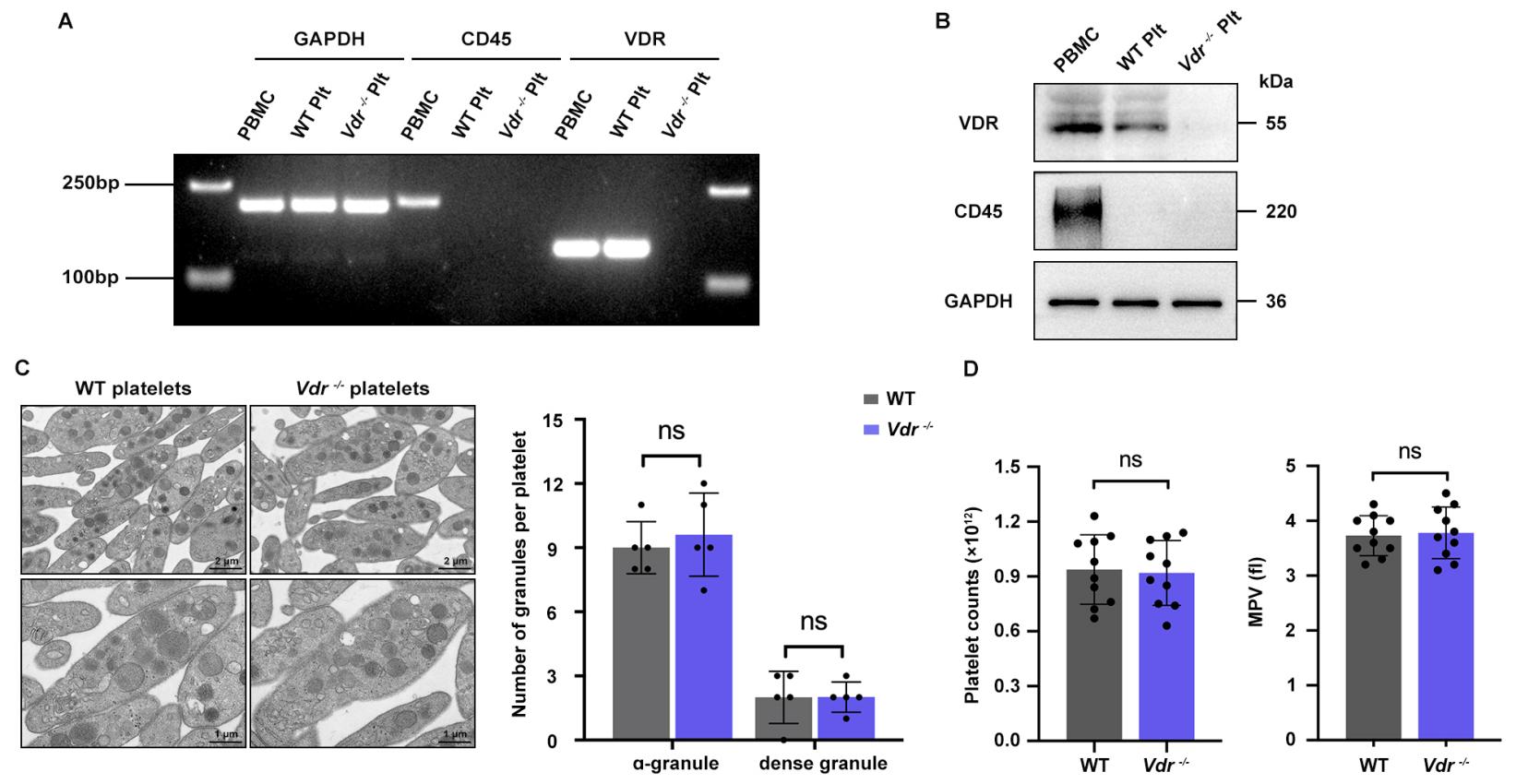
**

**Supplementary Figure 7. Platelet ultrastructure and count remained unchanged in VDR-deficient mice.**

**(A)** Reverse transcription-polymerase chain reaction analysis was performed to quantify VDR mRNA expression in WT mouse PBMC, WT mouse platelets, and VDR-deficient (*Vdr*^-/-^) mouse platelets. The white blood cell-specific marker CD45 was used to confirm the absence of white blood cell contamination in the platelet samples. **(B)** Western blot analysis was conducted to assess VDR protein expression in WT mouse PBMC, WT mouse platelets, and *Vdr*^-/-^ mouse platelets. CD45 was employed to verify the purity of the isolated mouse platelets. **(C)** Quantification of α-granules and dense granules in WT and *Vdr*^-/-^ mice (n=5). **(D)** Assessment of platelet count and MPV in WT and *Vdr*^-/-^ mice (n=10). Data were analyzed by unpaired Student’s *t*-test in **(C)**-**(D)**. Data are presented as mean ± SD. ns, not significant. PBMC, peripheral blood mononuclear cell; MPV, mean platelet volume; VDR, vitamin D receptor; WT, wild-type.


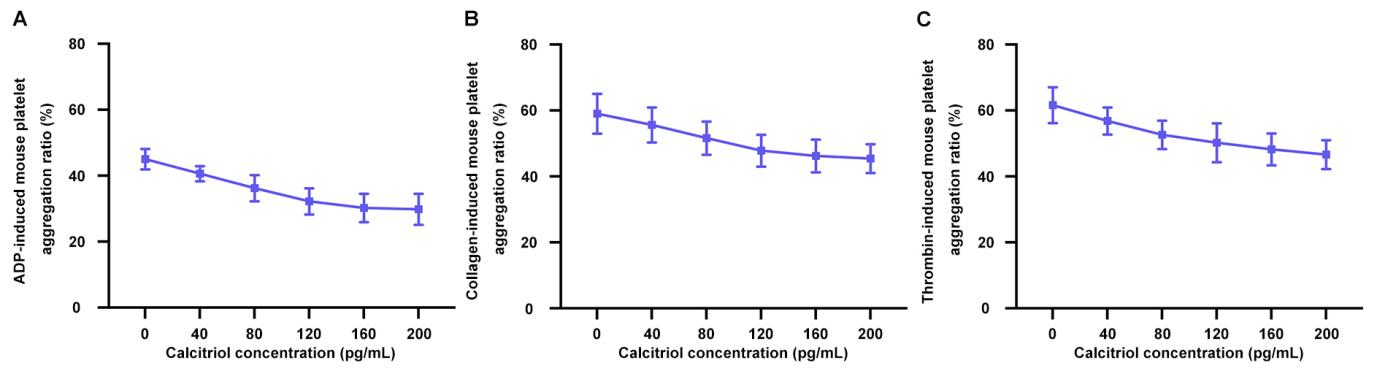


**Supplementary Figure 8. Calcitriol dose titration for mouse platelet function.**

**(A-C)** The association between calcitriol concentration and washed wild-type platelet aggregation induced by ADP (3 μM) **(A)**, collagen (0.3 μg/mL) **(B)**, or thrombin (0.03 U/mL) **(C)**. Summary data are presented (n=5, each concentration point). ADP, adenosine diphosphate.

**
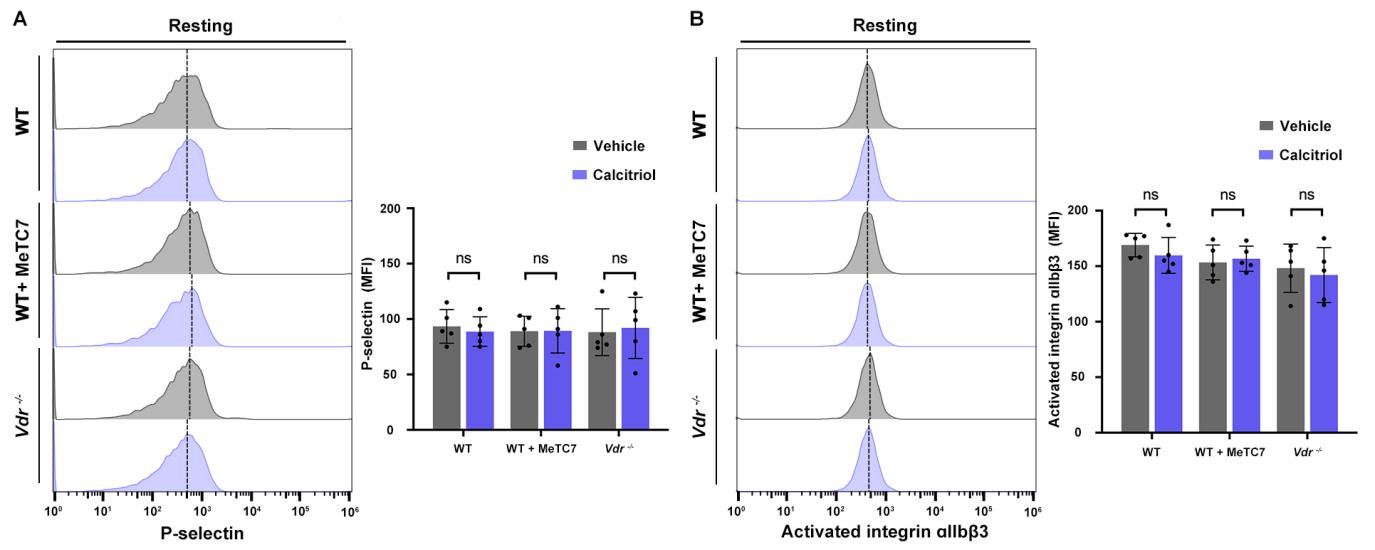
**

**Supplementary Figure 9. The resting status of P-selectin exposure and JON/A binding in Figure 3D and 3E.**

**(A)** P-selectin surface expression was evaluated on washed mouse platelets isolated from WT and VDR-deficient (*Vdr*^-/-^) mice under various resting conditions (n=5). **(B)** JON/A binding to washed WT and *Vdr^-/-^* mouse platelets was assessed in the resting state after different treatments (n=5). Data were analyzed by one-way ANOVA followed by Sidak’s multiple comparisons in **(A)**-**(B)**. Data are presented as mean ± SD. ns, not significant. WT, wild-type.

**
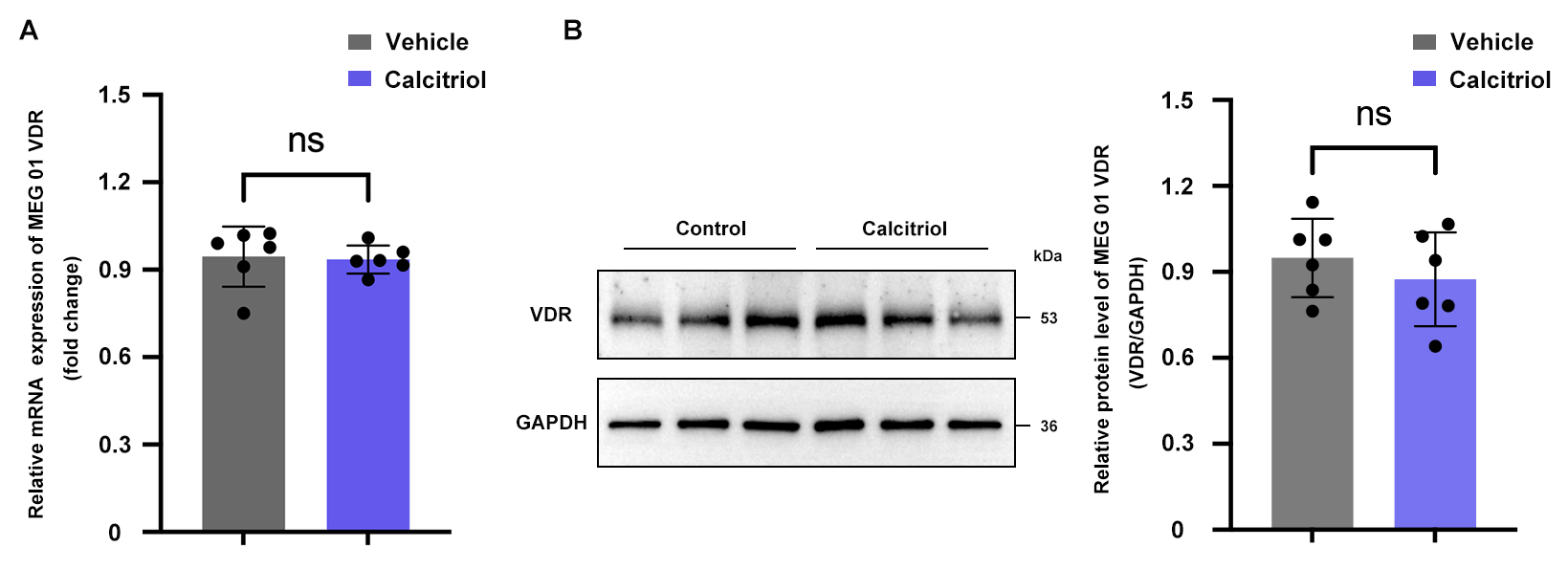
**

**Supplementary Figure 10. VDR expression in Meg-01 cells remained unchanged following calcitriol treatment.**

**(A)** Quantitative polymerase chain reaction analysis of VDR mRNA expression in Meg-01 cells treated with calcitriol or a vehicle control (n=6). **(B)** Western blot analysis of VDR protein expression in Meg-01 cells treated with calcitriol or a vehicle control (n=6). Data were analyzed by unpaired Student’s *t*-test in **(A)**-**(B)**. Data are presented as mean ± SD. ns, not significant. VDR, vitamin D receptor.


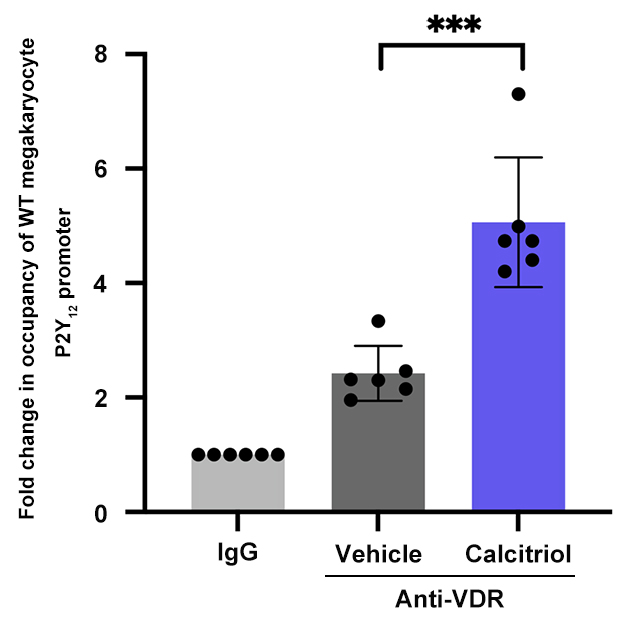


**Supplementary Figure 11. Calcitriol ingestion increased VDR enrichment within the P2Y_12_ promoter region in mouse megakaryocytes.**

Quantitative polymerase chain reaction analysis was performed to assess VDR binding to the P2Y_12_ promoter in megakaryocytes isolated from WT mice treated with calcitriol or a vehicle control. Chromatin immunoprecipitation was conducted using an anti-VDR antibody, with an isotype IgG serving as a negative control. Data were normalized to pre-immunoprecipitation input and expressed as fold change relative to the control group (n=6). Data were analyzed by one-way ANOVA with Dunnett’s multiple comparisons. Data are presented as mean ± SD. ***P<0.001. VDR, vitamin D receptor; WT, wild-type.


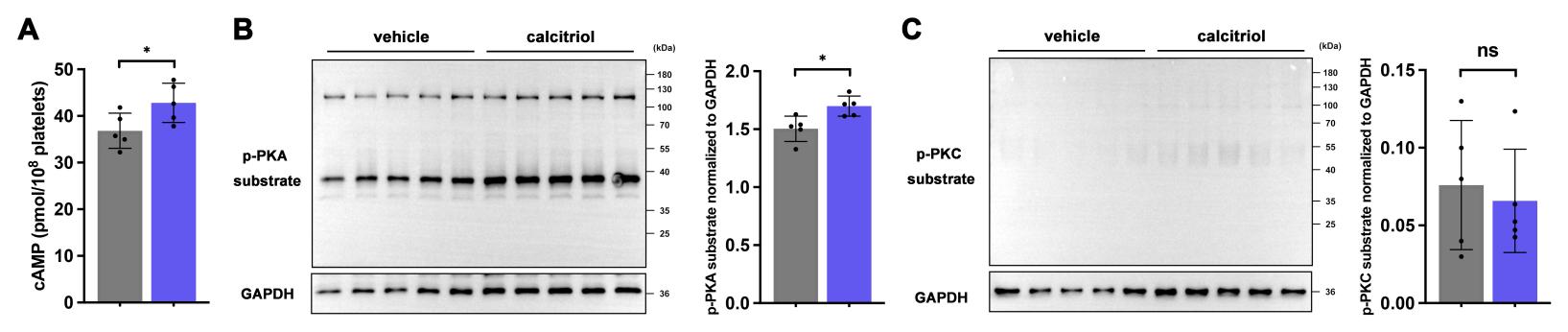


**Supplementary Figure 12. Calcitriol directly modulates the cAMP-PKA pathway in resting platelets.**

**(A)** Intracellular cAMP concentrations in resting platelets treated with calcitriol (40 pg/mL) or a vehicle control (n=5). **(B)** Western blot analysis and quantification of PKA substrate phosphorylation in resting platelets treated with calcitriol (40 pg/mL) or a vehicle control (n=5). **(C)** Western blot analysis and quantification of PKC substrate phosphorylation in resting platelets treated with calcitriol (40 pg/mL) or a vehicle control (n=5). Data were analyzed by unpaired Student’s *t*-test in **(A)**-**(C)**. Data are presented as mean ± SD. *P < 0.05; ns, not significant. cAMP, cyclic adenosine monophosphate; PKA, protein kinase A; PKC, protein kinase C; GAPDH, glyceraldehyde-3-phosphate dehydrogenase.


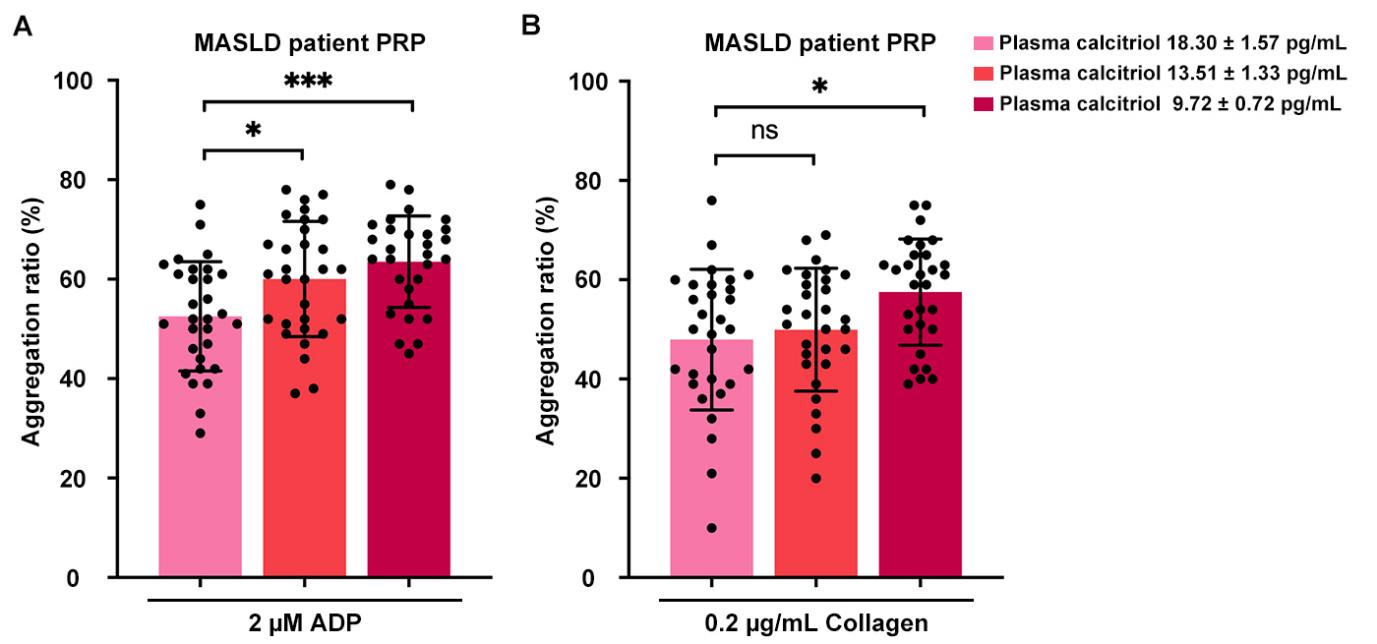


Supplementary Figure 13. The severity of vitamin D deficiency was associated with clinically significant platelet hyperactivation in MASLD patients.

(A-B) Summary data of platelet aggregation in PRP from MASLD patients, categorized into groups with mild (18.30 ± 1.57 ng/mL, n=30), moderate (13.51 ± 1.33 ng/mL, n=30), and severe (9.72 ± 0.72 ng/mL, n=29) vitamin D deficiency based on baseline plasma vitamin D tertiles, were obtained following stimulation with ADP (A) or collagen (B). Data were analyzed by one-way ANOVA with Dunnett’s multiple comparisons in (A) and (B). Data are presented as mean ± SD. *P<0.05; ***P<0.001; ns, not significant. ADP, adenosine diphosphate; MASLD, metabolic dysfunction-associated steatotic liver disease; PRP, platelet-rich plasma.


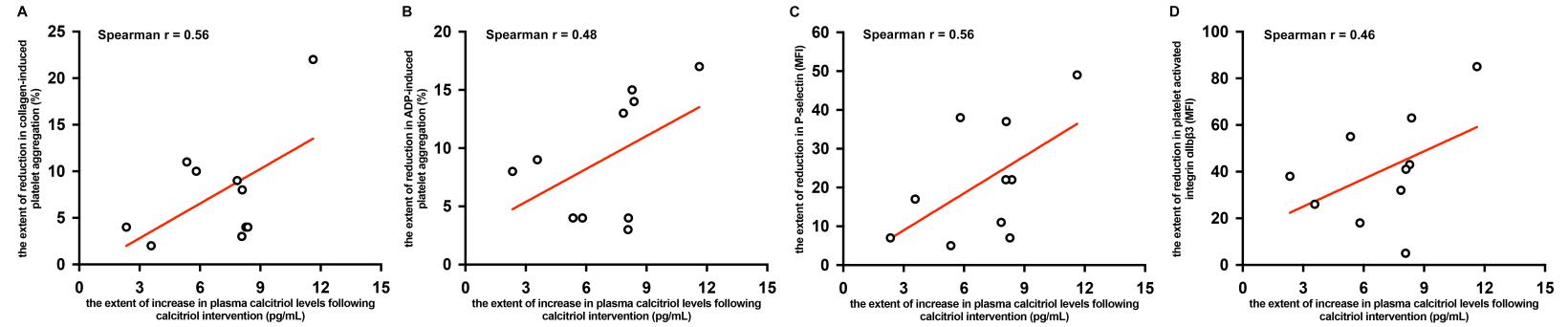


Supplementary Figure 14. **Correlation between the elevation of plasma calcitriol levels and the reduction of platelet reactivity in MASLD individuals.**

**(A)** Correlation between the extent of increase in plasma calcitriol levels and the extent of reduction in collagen-induced platelet aggregation. **(B)** Correlation between the extent of increase in plasma calcitriol levels and the extent of reduction in ADP-induced platelet aggregation. **(C)** Correlation between the extent of increase in plasma calcitriol levels and the extent of reduction in surface P-selectin expression. **(D)** Correlation between the extent of increase in plasma calcitriol levels and the extent of reduction in platelet activated integrin αIIbβ3 expression. Spearman r values are indicated on each plot. Data were analyzed by Spearman's rank correlation test. MASLD, metabolic dysfunction-associated steatotic liver disease; ADP, adenosine diphosphate; MFI, mean fluorescence intensity.

**
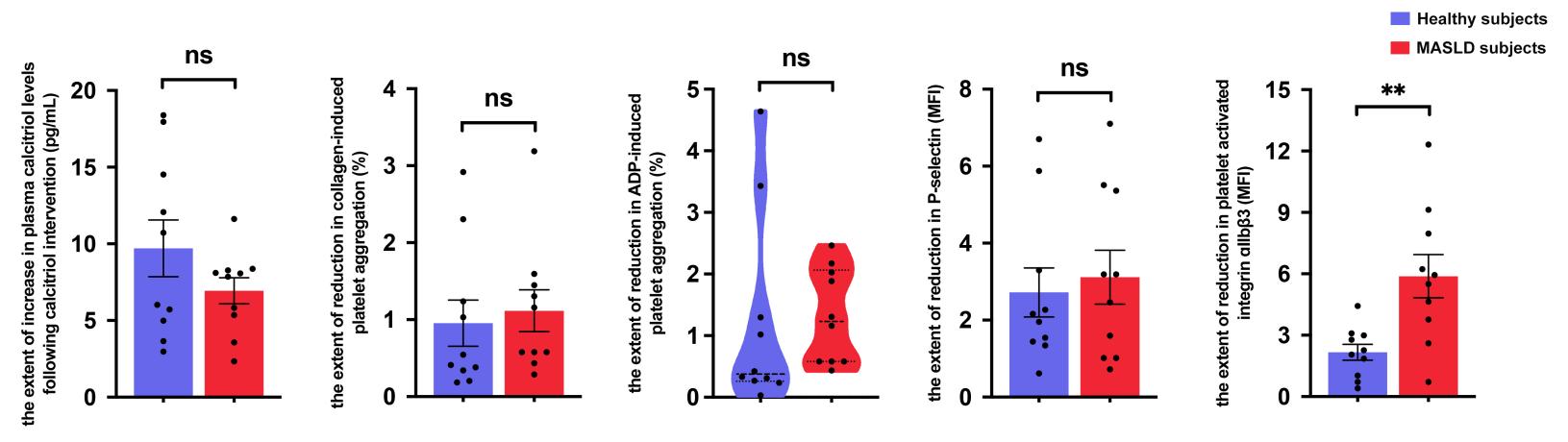
**

Supplementary Figure 15. Comparative analysis of platelet sensitivity to calcitriol in healthy and MASLD individuals.

The reduction in each platelet reactivity marker was normalized to the corresponding systemic increase in plasma calcitriol levels for each participant (Δplatelet marker/Δplasma calcitriol). The markers include the normalized changes in collagen-induced platelet aggregation, ADP-induced platelet aggregation, surface P-selectin expression, and activated integrin ɑIIbβ3 expression. Data for ADP-induced platelet aggregation were analyzed using the Mann-Whitney *U* test and are presented as median [25th, 75th percentiles]; other parameters were analyzed using the unpaired Student’s *t*-test and are presented as mean ± SEM. **P < 0.01; ns, not significant. MASLD, metabolic dysfunction-associated steatotic liver disease; ADP, adenosine diphosphate; MFI, mean fluorescence intensity.


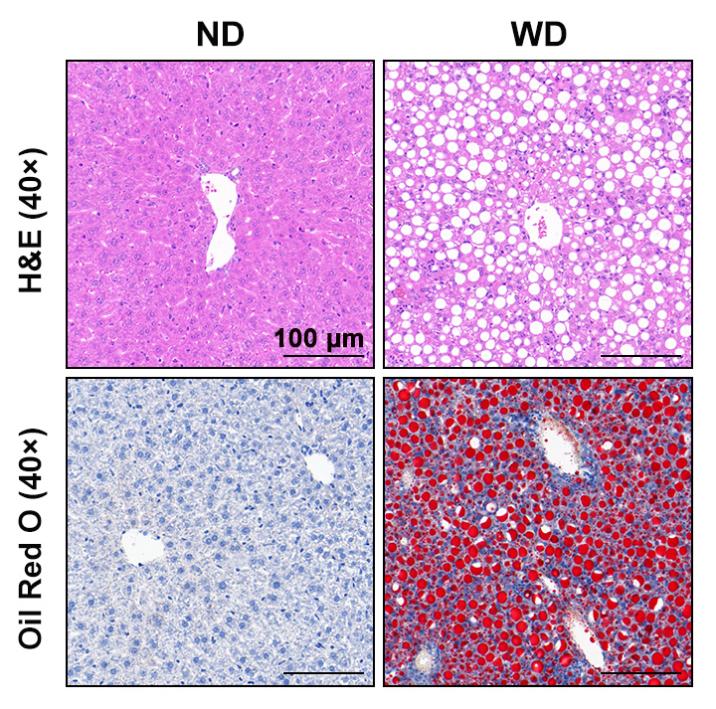


**Supplementary Figure 16. MASLD induced by a MCDD in WT mice.**

Representative images of ballooning and lipid accumulation were presented for WT mice fed a ND or a MCDD. MCDD, methionine- and choline-deficient diet; MASLD, metabolic dysfunction-associated steatotic liver disease; ND, normal diet; WT, wild-type.


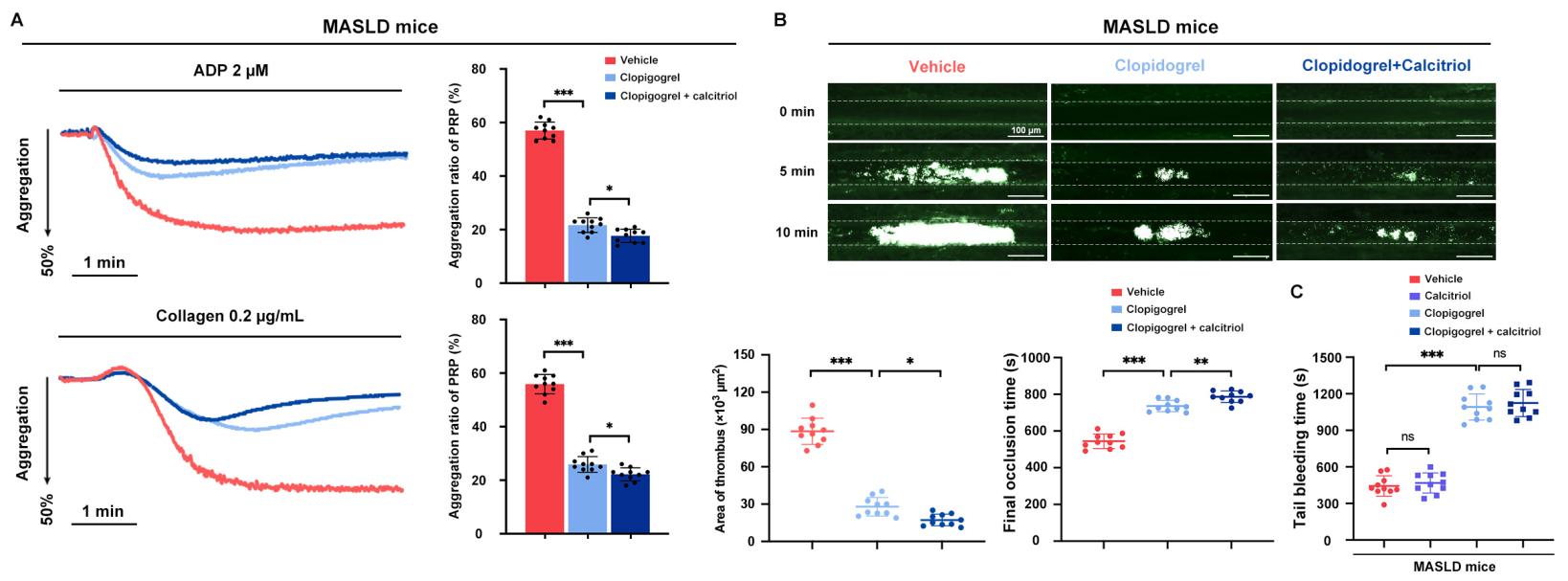


**Supplementary Figure 17. Calcitriol potentiates the antiplatelet effects of P2Y_12_ inhibitors in MASLD mice.**

**(A)** Representative traces and quantitative analysis of platelet aggregation induced by ADP or collagen in PRP from MASLD mice treated with vehicle, clopidogrel (10 mg/kg, p.o., single dose), or clopidogrel (10 mg/kg, p.o., single dose) + calcitriol (50 ng/kg, p.o., two weeks) (n=10). **(B)** Representative images and quantitative analysis of FeCl_3_-induced thrombus formation in MASLD mice treated with vehicle, clopidogrel, or clopidogrel + calcitriol (n=10). **(C)** Tail bleeding time in MASLD mice treated with vehicle, calcitriol (50 ng/kg, p.o., two weeks), clopidogrel, or clopidogrel + calcitriol (n=10). Data were analyzed by one-way ANOVA with Dunnett’s multiple comparisons in **(A)**-**(C)**. Data are presented as mean ± SD. *P<0.05; **P<0.01; ***P<0.001; ns, not significant. ADP, adenosine diphosphate; FeCl_3_, ferric trichloride; MASLD, metabolic dysfunction-associated steatotic liver disease; PRP, platelet-rich plasma.


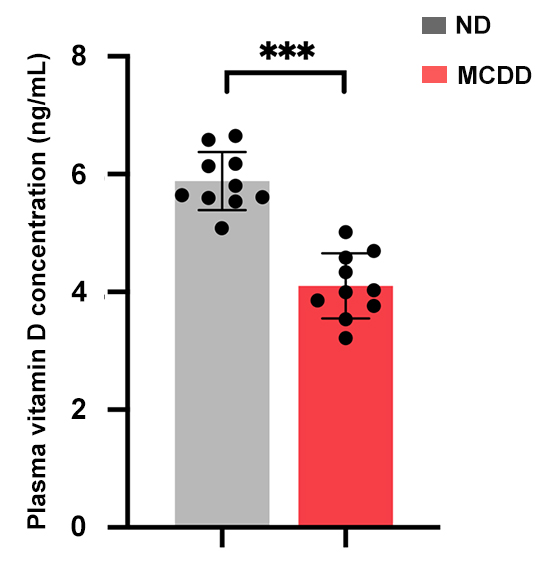


**Supplementary Figure 18. MCDD-induced MASLD is associated with a significant decrease in vitamin D levels.**

Plasma vitamin D levels were quantified in WT mice fed a normal diet or a MCDD. Summary data from 10 independent experiments are presented. Data were analyzed by unpaired Student’s *t*-test. Data are presented as mean ± SD. ***P<0.001. ND, normal diet; MASLD, metabolic dysfunction-associated steatotic liver disease; MCDD, methionine- and choline-deficient diet; WT, wild-type.

**
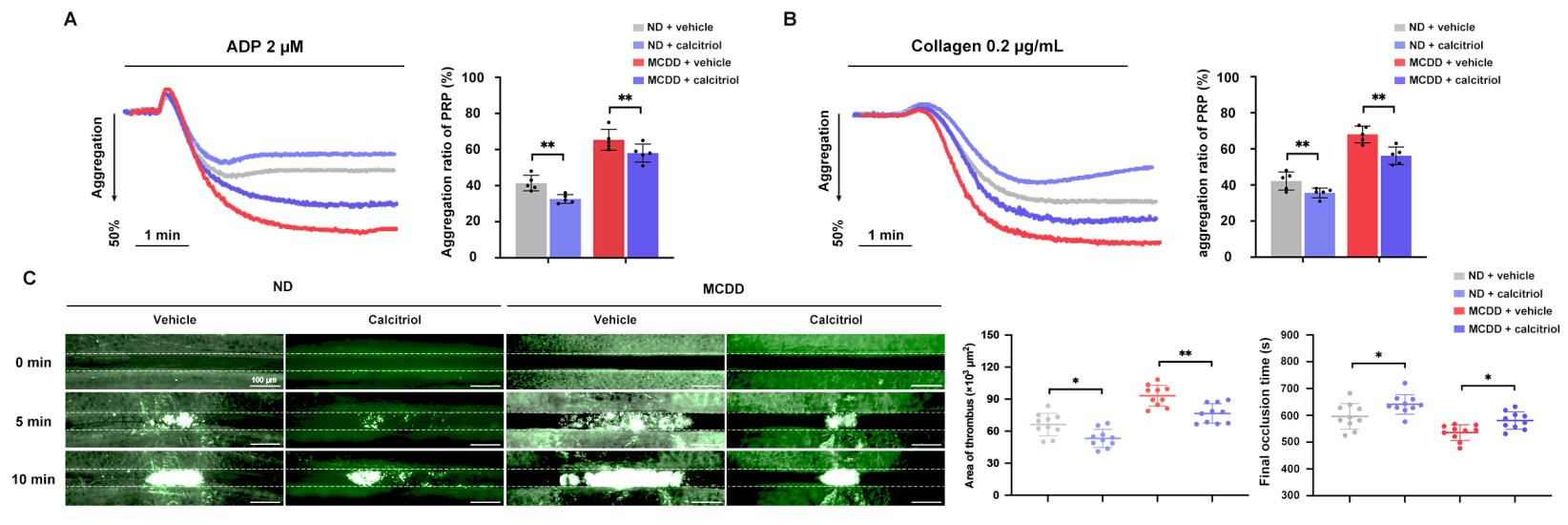
**

**Supplementary Figure 19. Calcitriol inhibits platelet function and thrombosis in mice with MCDD-induced MASLD.**

**(A-B)** Representative traces and quantitative analysis of platelet aggregation were performed in PRP from mice fed a ND or a MCDD, treated with vehicle or calcitriol, and stimulated with ADP **(A)** or collagen **(B)** (n=5). **(C)** Representative images and quantitative analysis of FeCl_3_-induced thrombus formation were performed in mice fed a ND (n=10). Platelet-deprived ND mice were subsequently infused with platelets from either calcitriol- or vehicle-treated ND or MCDD-fed mice before FeCl_3_-induced mesenteric artery injury. Data were analyzed by one-way ANOVA with Sidak’s multiple comparisons in **(A)**-**(C)**. Data are presented as mean ± SD. *P<0.05; **P<0.01. ADP, adenosine diphosphate; FeCl_3_, ferric trichloride; ND, normal diet; MASLD, metabolic dysfunction-associated steatotic liver disease; MCDD, methionine- and choline-deficient diet; PRP, platelet-rich plasma.


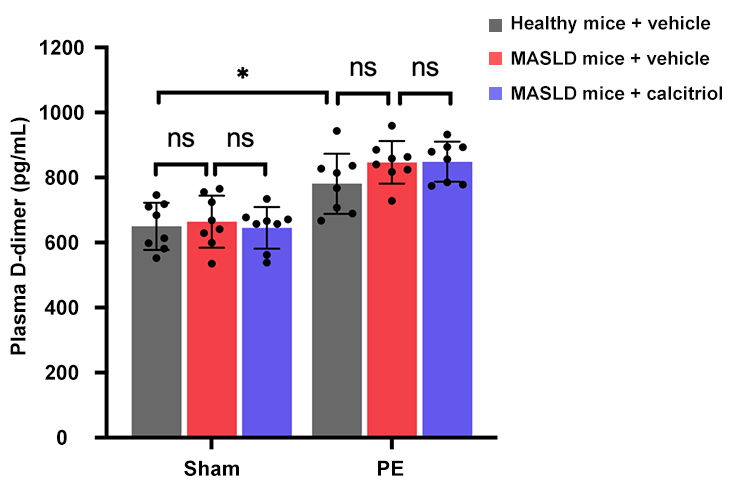


**Supplementary Figure 20. Calcitriol did not significantly affect fibrinolytic activity in MASLD mice injected with collagen and epinephrine.**

MASLD and healthy mice received daily calcitriol (50 ng/kg) or vehicle injections for two weeks, with the final dose administered 5 hours before collagen and epinephrine injection (n=8). Sham groups received saline injections of the equivalent volume. Plasma D-dimer levels were measured by ELISA 48 hours following collagen and epinephrine injection. Data were analyzed by two-way ANOVA with Tukey’s multiple comparisons test. Data are presented as mean ± SD. *P<0.05; ns, not significant. ELISA, enzyme-linked immunosorbent assay; MASLD, metabolic dysfunction-associated steatotic liver disease; PE, pulmonary embolism.

**
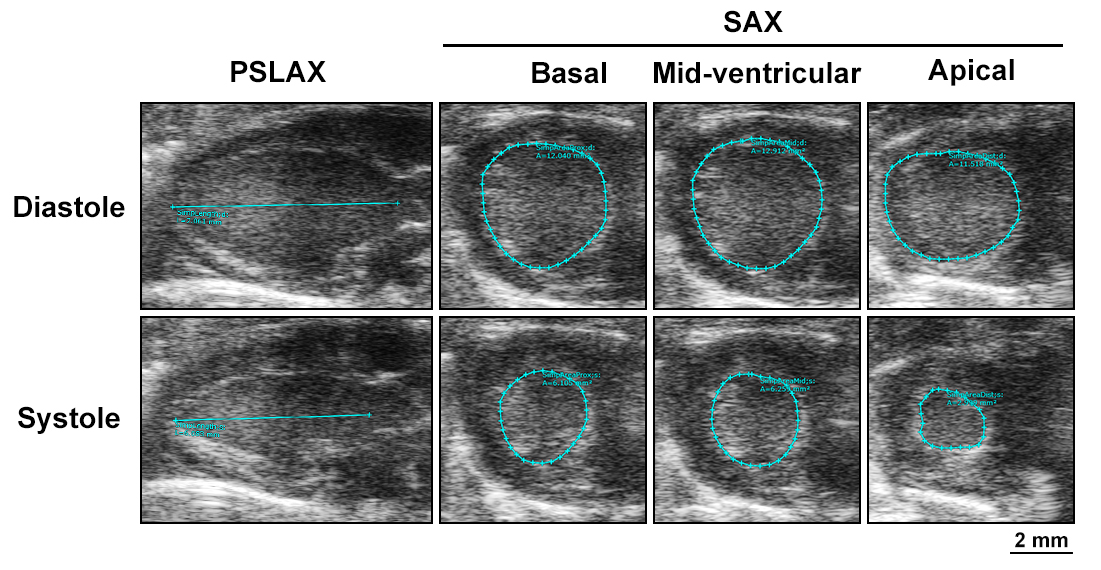
**

**Supplementary Figure 21. Determination of ventricular volume by the modified Simpson method.**

The biplane modified Simpson method calculates ventricular volume using the ventricular length from a PSLAX view (blue lines) and left ventricular areas from three orthogonal SAX views (midventricular, apical, and basal; blue circles). PSLAX, parasternal long-axis; SAX, short-axis.

**
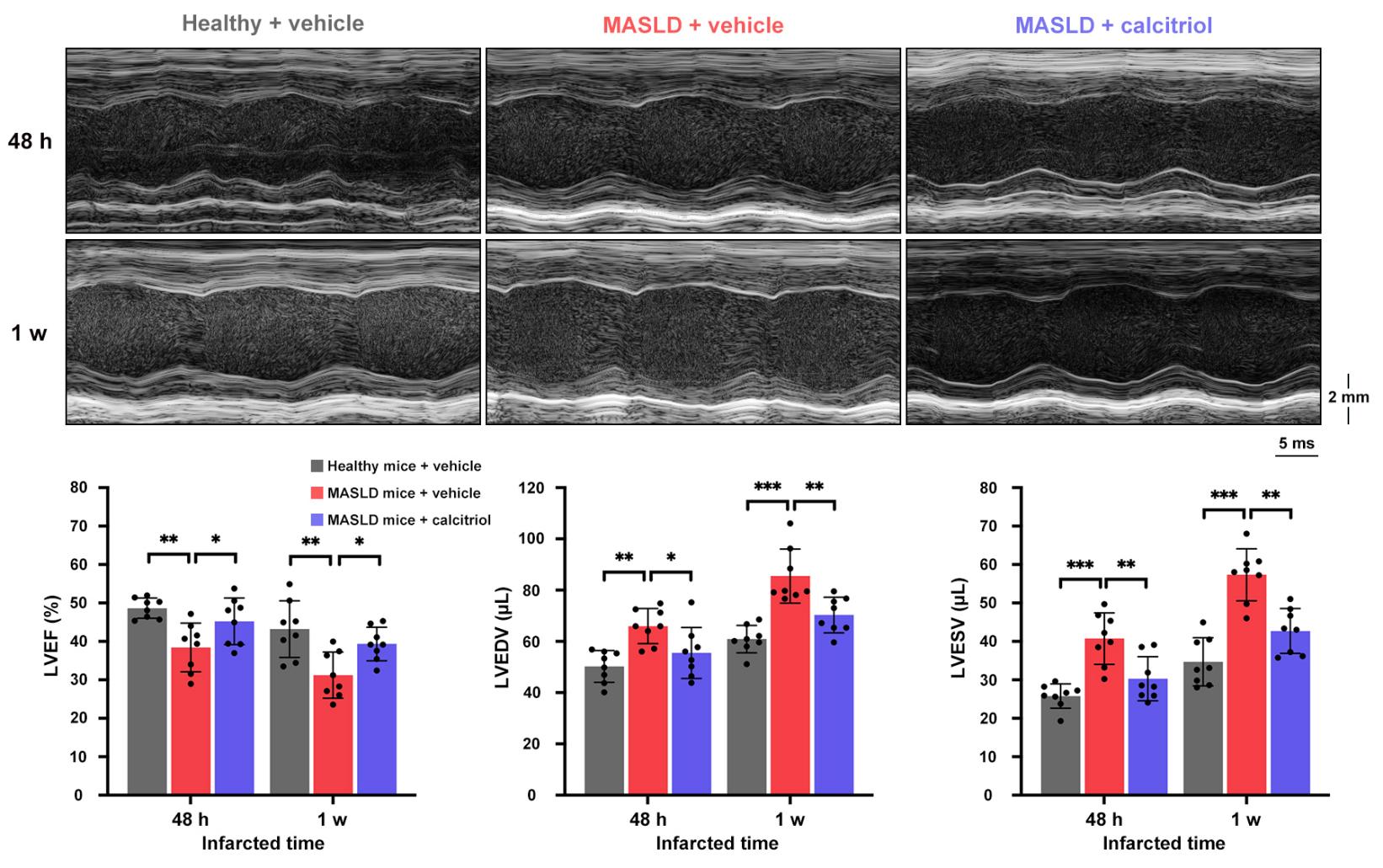
**

**Supplementary Figure 22. Calcitriol improved cardiac function in MASLD mice following myocardial I/R injury, evidenced by M-mode echocardiography.**

Representative M-mode echocardiograms from vehicle or calcitriol pretreated healthy and MASLD mice following myocardial I/R injury. LVEF, LVEDV, and LVESV were quantified at 48 hours and 1 week post-I/R. Representative images and summary data are presented (n=8). Data were analyzed by one-way ANOVA with Dunnett’s multiple comparisons. Data are presented as mean ± SD. *P<0.05; **P<0.01; ***P<0.001. I/R, ischemia-reperfusion; LVEF, left ventricular ejection fraction; LVEDV, left ventricular end-diastolic volume; LVESV, left ventricular end-systolic volume; MASLD, metabolic dysfunction-associated steatotic liver disease.

**
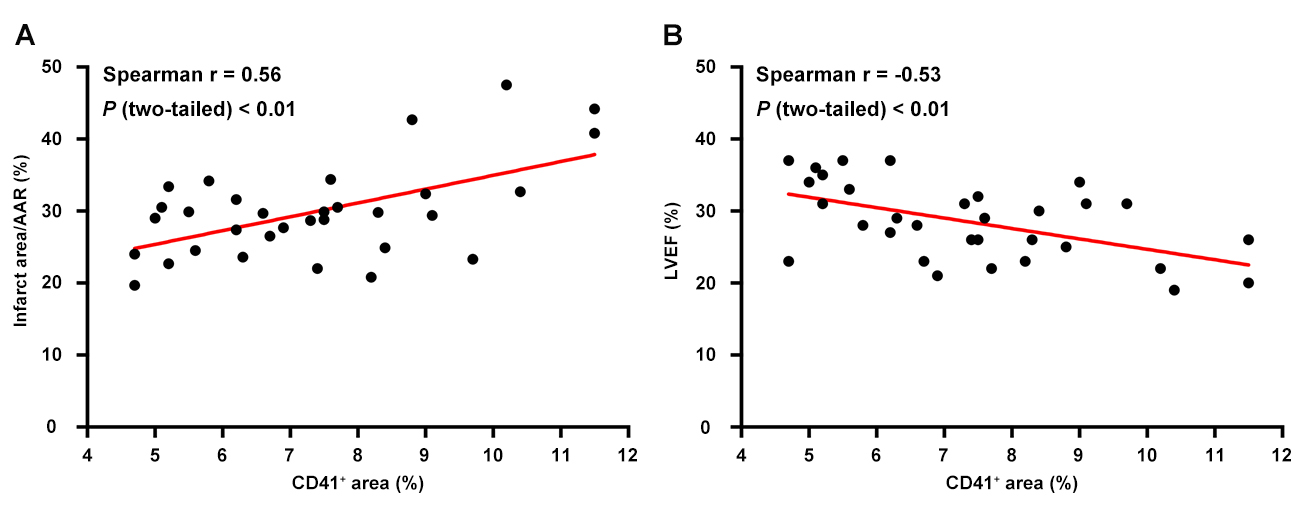
**

**Supplementary Figure 23. Microvascular thrombosis is closely correlated with myocardial infarct area and LVEF in MASLD mice.**

**(A-B)** In MASLD mice, microvascular thrombosis area (CD41^+^) showed a significant positive correlation with infarct size (Spearman r = 0.56, *P* < 0.01) **(A)** and a significant inverse correlation with LVEF (Spearman r = -0.53, *P* < 0.01) **(B)**. Each data point represents an individual mouse (n=32). LVEF, left ventricular ejection fraction; MASLD, metabolic dysfunction-associated steatotic liver disease.

**
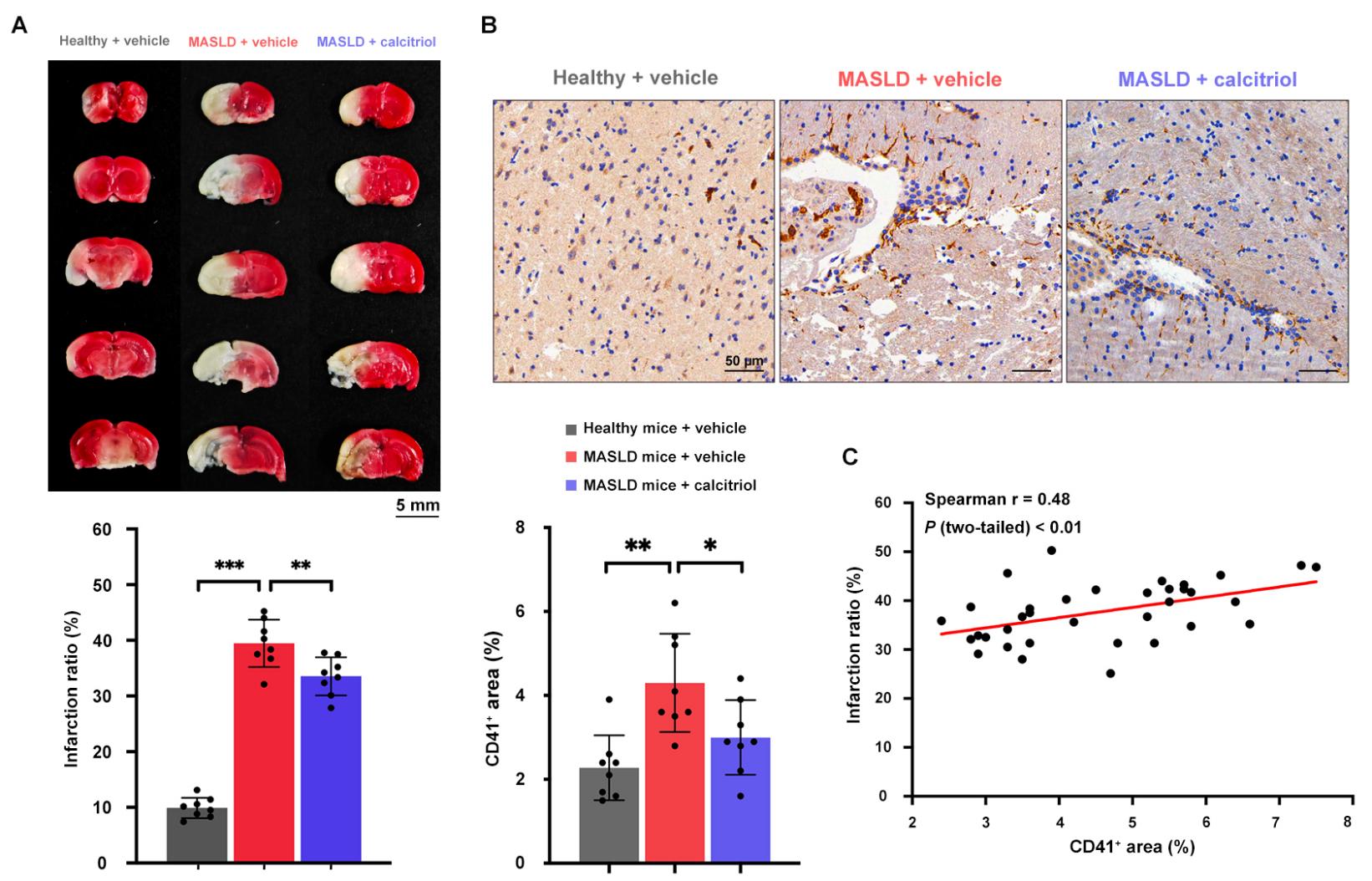
**

**Supplementary Figure 24. Calcitriol protects MASLD mice from severe brain infarction after MCAO.**

1. Representative TTC-stained brain tissue sections from healthy and MASLD mice, pretreated with vehicle or calcitriol, and then subjected to middle cerebral artery occlusion (n=8). Red areas indicate viable tissue, while white areas indicate infarcted regions. **(B)** Immunohistochemical images depict infarct brain tissue from healthy and MASLD mice subjected to MCAO, with vehicle or calcitriol pretreatment. Quantification of intracerebral platelet positivity is presented. Representative images and summary data are shown (n=8). **(C)** In MASLD mice, the intracerebral area of platelet positivity (CD41^+^) demonstrated a significant positive correlation with brain infarct size (Spearman r = 0.48, P < 0.01). Each data point represents an individual mouse (n=35). Data were analyzed by one-way ANOVA with Dunnett’s multiple comparisons in **(A)** and **(B)**. Data are presented as mean ± SD. *P<0.05; **P<0.01; ***P<0.001. MCAO, middle cerebral artery occlusion; MASLD, metabolic dysfunction-associated steatotic liver disease; TTC, triphenyl tetrazolium chloride.
